# Efficient Tandem Addition/Cyclization for Access to 2,4-Diarylquinazolines via Catalytic Carbopalladation of Nitriles

**DOI:** 10.3390/molecules24030463

**Published:** 2019-01-28

**Authors:** Julin Gong, Kun Hu, Yetong Zhang, Yinlin Shao, Tianxing Cheng, Maolin Hu, Jiuxi Chen

**Affiliations:** College of Chemistry & Materials Engineering, Wenzhou University, Wenzhou 325035, China; 16451282199@stu.wzu.edu.cn (J.G.); hukun15@163.com (K.H.); 16451282259@stu.wzu.edu.cn (Y.Z.); ctx@wzu.edu.cn (T.C.); jiuxichen@wzu.edu.cn (J.C.)

**Keywords:** palladium-catalyzed, tandem reaction, nitrile, carbopalladation, quinazoline

## Abstract

The first example of the palladium-catalyzed tandem addition/cyclization of 2-(benzylidenamino)benzonitriles with arylboronic acids has been developed. This transformation features good functional group tolerance and provides an alternative synthetic pathway to access 2,4-diarylquinazolines in moderate to good yields. A plausible mechanism for the formation of 2,4-diarylquinazolines involving sequential nucleophilic addition followed by an intramolecular cyclization is proposed.

## 1. Introduction

**Scheme 1:**
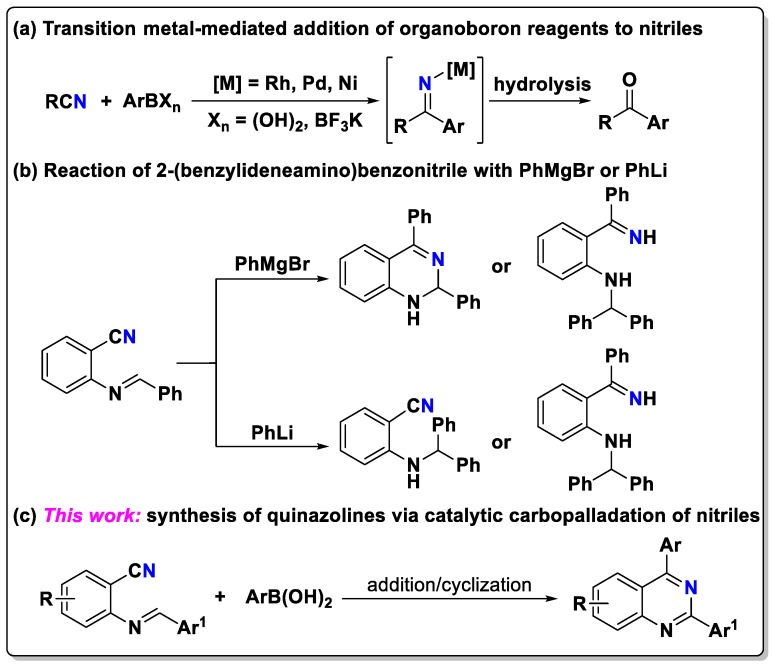
Design of new approach to 2,4-diarylquinazolines.

It is well-known that nitriles such as acetonitrile or benzonitrile are widely used as solvents or ligands in organometallic reactions, presumably due to the inherently inert nature of the cyano group [1,2,3]. The development of inert C≡N bond activation/carbon-carbon or carbon-heteroatom bond-forming reactions catalyzed by transition metals has attracted significant attention of organic chemists during the past several decades [4]. Since Larock’s pioneering work on the development of the catalytic carbopalladation of nitriles [5,6,7], remarkable progress in the transition metal catalyzed addition of organoboron reagents to nitriles has been documented during the past several decades by several other groups [8,9,10,11] and our group [12,13,14]. In recent years, the scope of this chemistry has been significantly expanded to other coupling partners, including sodium aryl sulfinates or arylsulfinic acids [15,16,17,18], aryl halides [19,20], benzoic acids [21], arylhydrazines [22], and arylsulfonyl hydrazides [23]. However, this transformation of nitriles exclusively provides aryl ketone products (Scheme 1a). Therefore, the development of an efficient method that can incorporate the nitrogen atom of nitriles into *N*-heterocycle products by intramolecular cyclization, rather than hydrolysis of ketamine intermediates, still remains a longstanding challenge. In 2017, we have successfully developed a tandem addition and cyclization strategy for the synthesis of isoquinolines and isoquinolones via catalytic carbopalladation of nitriles [24,25].

Quinazolines have attracted increasing attention in the past few years because of their broad applications in medicinal chemistry [26,27,28,29,30], material chemistry [31,32,33], and catalysis [34]. Hence, the design of effective methods for the construction of quinazolines has been an active area of research in organic chemistry [35,36,37]. Although the transformation of nitriles into various functional groups is well-established, only sporadic examples of the synthesis of quinazolines from nitriles have been reported to date. In 1988, Stxekowski and co-workers reported additions of Grignard reagents (or lithium reagents) to 2-(benzylideneaminoi)benzonitrile (Scheme 1b) [38]. For example, treatment of 2-(benzylideneamino)benzonitrile with phenylmagnesium bromide in THF delivered the cyclization product 2,4-diphenyl-1,2-dihydroquinazoline and the adduct *N*-benzhydryl-2-(imino-(phenyl)-methyl)aniline in a 3:1 ratio. However, the reaction of 2-(benzylideneamino)benzonitrile with phenyllithium gave the adducts 2-(benzhydrylamino)benzonitrile or *N*-benzhydryl-2-(imino- (phenyl)methyl)aniline. In recent years, Chen [39] and Liu [40] independently reported syntheses of quinazolines via a 2 + 2 + 2 cascade annulation of diaryliodonium salts (or aryldiazonium salts) with two nitriles. Replacing Grignard reagents, organolithium reagents, diaryliodonium salts or aryldiazonium salts with organoboron reagents such as arylboronic acids is more desirable due to their low toxicity, ease of handling, and good functional group tolerance. Very recently, we developed a palladium-catalyzed tandem reaction of functionalized nitriles (e.g., 2-(quinazolinone-3(4*H*)-yl)benzonitriles [41] or *N*-(2-cyanoaryl)benzamides [42]) with arylboronic acids for the synthesis of quinazolines.

As part of our efforts in our laboratory toward the development of the catalytic carbopalladation of nitriles, we herein report a palladium-catalyzed tandem addition/cyclization of 2-(benzylideneamino)benzonitriles with arylboronic acids to afford 2,4-diarylquinazolines (Scheme 1c). It is noteworthy that this protocol provides the same 2,4-diarylquinazoline products as our previous work [42], ultimately from the same starting materials (2-aminobenzonitrile and arylboronic acid).

## 2. Results and Discussion

We began our investigation by examining the reaction between readily available 2-(benzylideneamino)benzonitrile (**1a**) and phenylboronic acid (**2a**) to establish the optimal reaction conditions (Table 1). Trace amounts of the desired product 2,4-diphenylquinazoline (**3a**) was detected by GC/MS analysis when the combination of Pd(PPh_3_)_4_, trifluoromethanesulfonic acid (TfOH) and 2,2′-bipyridine (**L1**) was used in THF/H_2_O (entry 1). The yield of **3a** could be improved to 15% using PdCl_2_ as a catalyst (entry 2). Among the palladium catalysts used (entries 3–6), Pd(acac)_2_ exhibited the highest catalytic reactivity, giving a 27% yield (entry 6). Next, various bidentate ligands **L2**−**L7** were evaluated (entries 7-12) and 5,5′-dimethyl-2,2′-bipyridine (**L2**) afforded the best result (45% yield, entry 7). In contrast, little to no product **3a** was detected when sterically hindered ligands such as 6,6′-dimethyl-2,2′-bipyridine (**L4**), 2,2′-biquinoline (**L5**) and 2,9-dimethyl-1,10-phenanthroline (**L6**) were used (entries 9–11). An investigation of the effect of solvent revealed that the yield of **3a** was greatly increased to 57% in DMF (entry 17). Other solvents, including H_2_O, toluene, 1,4-dioxane, and dimethylacetamide (DMA), were less efficient (entries 13–17). Replacement of TfOH with other additives, including acetic acid (AcOH), trifluoroacetic acid (TFA), D-camphorsulfonic acid (CSA), resulted in lower yields (entries 18-20). However, *p*-toluenesulfonic acid monohydrate (TsOH·H_2_O) effectively promoted this reaction and exhibited the highest catalytic reactivity with 81% yield (entry 21). The reaction did not work using HCl as an addition (entry 22). The desired product **3a** was not detected if either palladium catalyst or additive was absent (entries 23–24).

**Scheme 2:**
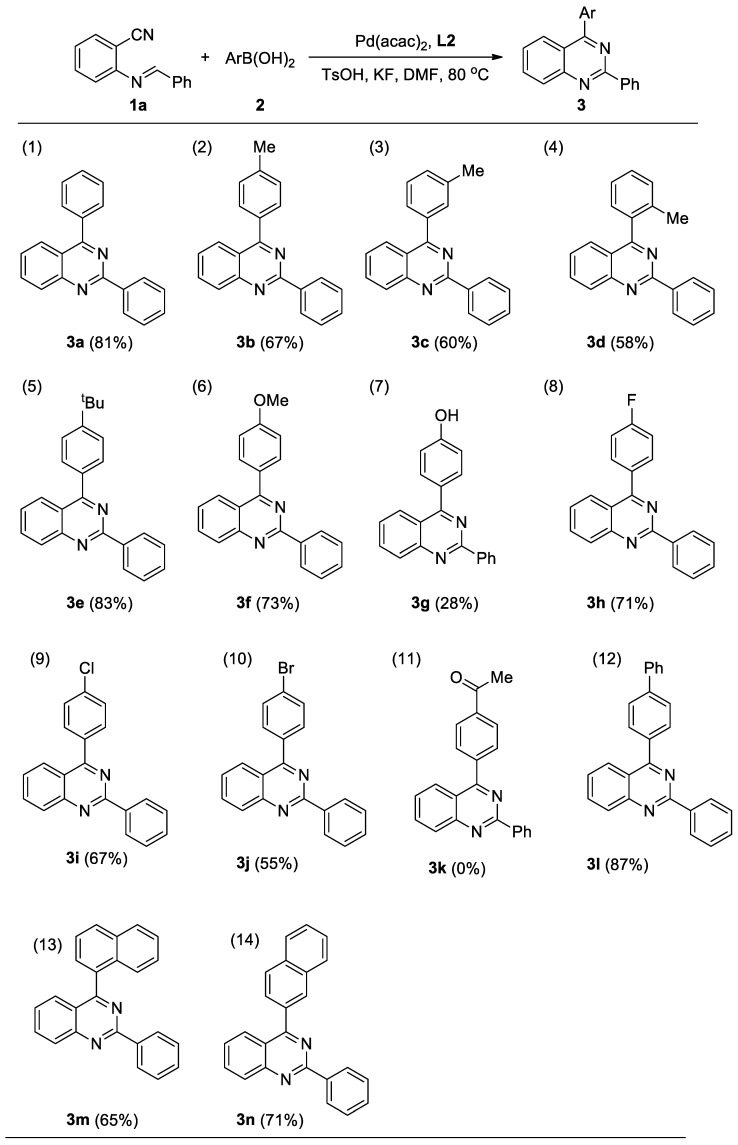
Tandem reaction of 2-(benzylideneamino)benzonitrile with arylboronic acid ^a^. ^a^ Reaction conditions: **1a** (0.3 mmol), **2** (0.6 mmol), Pd(acac)_2_ (10 mol %), L**2** (20 mol %), DMF (2 mL), TsOH·H_2_O (4 equiv.), KF (2 equiv.), 80 °C, 48 h, air. Isolated yield.

With the optimized reaction conditions in hand, we evaluated the substrates scope of the tandem reaction. First, the tandem reaction between 2-(benzylideneamino)benzonitrile (**1a**) and various arylboronic acids were investigated under standard conditions (Scheme 2). The influence of substituents at the phenyl ring of arylboronic acid was examined, and the results demonstrated that steric effects of substituents had only a small influence on the yield. 

For example, the tandem reaction of **1a** with *para*- and *meta*-tolylboronic acid gave yields of 67% and 60%, respectively (**3b**, **3c**), while the *ortho*-tolylboronic acid afforded a yield of 58% (**3d**) (entries 2–4).The electronic properties of the substituents of arylboronic acid affected the yield to some extent. In general, the aromatic amines bearing an electron-donating substituent (e.g., −*^t^*Bu, −OMe) (entries 5–6) delivered a slightly higher yield of the desired products than those analogues bearing a halo substituent (e.g., −F, −Cl, −Br) (entries 8–10). Gratifyingly, (4-hydroxyphenyl)boronic acid was treated with 2-(benzylideneamino)benzonitrile to afford the corresponding product **3g** in 28% yield (entry 7), in which hydroxyl group is hard to be compatible with Grignard reagent or our previous protocol [42]. The reaction did not work when (4-acetylphenyl)boronic acid was used as substrate (entry 11). Bicyclolboronic acids, such as biphenyl-4-ylboronic acid, naphthalen-1-ylboronic acid and naphthalen-2-ylboronic acid, were also good partners and reacted with **1a** efficiently, providing the corresponding products **3l**, **3m** and **3n** in 87%, 65% and 71% yields, respectively (entries 10–12).

**Scheme 3:**
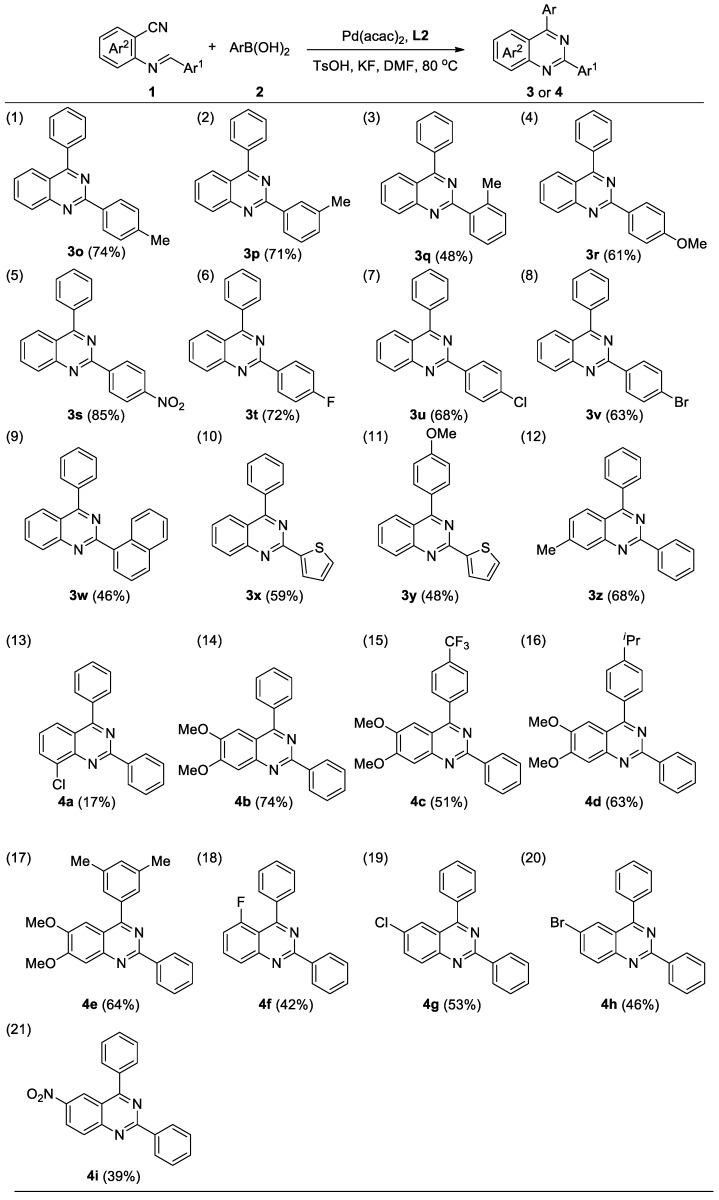
Tandem reaction of various 2-(benzylideneamino)benzonitriles with arylboronic acid ^a^. ^a^ Reaction conditions: **1a** (0.3 mmol), **2** (0.6 mmol), Pd(acac)_2_ (10 mol %), **L2** (20 mol %), DMF (3 mL), TsOH·H_2_O (4 equiv.), KF (2 equiv.), 80 °C, 48 h, air. Isolated yield.

We next turned our attention to the scope of this reaction with respect to the substituted 2-(benzylideneamino)benzonitriles substrate (Scheme 3). First, reaction of various 2-(benzylidene-amino)benzonitriles with phenylboronic acid was examined (entries 1–10). The influence of substitutions on the phenyl ring (Ar^1^) of the 2-(benzylideneamino)benzonitriles was first investigated. The steric effects of substituents had an obvious impact on the efficiency of this transformation. For example, when substrates bearing a *para*-, *meta*-, and *ortho*-methyl group were examined, **3o** and **3p** were obtained in 74% and 71% yield respectively, the yield of **3q** was decreased to 48% (entries 1–3). Both, substrates bearing a strong electron-donating (e.g., −OMe) (entry 4) or electron-withdrawing (e.g., −NO_2_) (entry 5) group were compatible with this reaction, affording the corresponding desired products **3r** and **3s** in 61% and 85% yields, respectively. Moreover, halogen-substituted (e.g., –F, –Cl, –Br) substrates were well tolerated and gave the desired products **3t**–**3v** in 63–72% yields (entries 6–8). The substrate bearing a naphthyl group, when treated with phenylboronic acid, delivered product **3w** in slightly lower yield (entry 9). 2-((Thiophen-2-yl-methylene)amino)benzonitriles bearing a thienyl group were also well tolerated, affording the corresponding products **3x** and **3y** in 59% and 48% yields, respectively (entries 10–11), which were hard to achieve cyclization products by our previous method [42]. Finally, we turned our attention to the effect of the various substituents on the aminobenzonitrile ring. The reaction of methyl-, methoxy-, NO_2_- and halogen-substituted (e.g., –F, –Cl, –Br) substrates with arylboronic acid also proceeded smoothly and the desired products **3z**–**4i** were isolated in moderate yields (entries 12–21). The low yield of these reactions mainly caused by the competing hydrolysis of imines to 2-aminobenzonitriles. It is worth noting that the presence of the halogen in the products (e.g., **3i**, **3v**, **4h**) is very useful for further synthetic elaborations thereby broadening the diversity of the products.

**Scheme 4:**
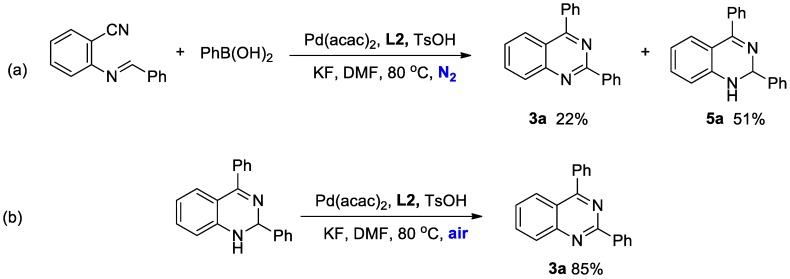
Control experiments.

To gain insight into the oxidative tandem carbopalladation cyclization reaction mechanism, further experiments were performed, as shown in Scheme 4. First, the reaction was carried under N_2_ atmosphere and the yield of the desired product **3a** decreased to 22%, accompany with unoxidized 2,4-diphenyl-1,2-dihydroquinazoline (**5a**) in 51% yield (Scheme 4a), indicating that this reaction is aerobic and the air would be the oxidizing agent. The desired product **3a** could also be obtained in 85% yield when the reaction of **5a** was performed under standard conditions without phenylboronic acid (Scheme 4b). These results implicate **5a** as possible intermediate for this transformation.

**Scheme 5:**
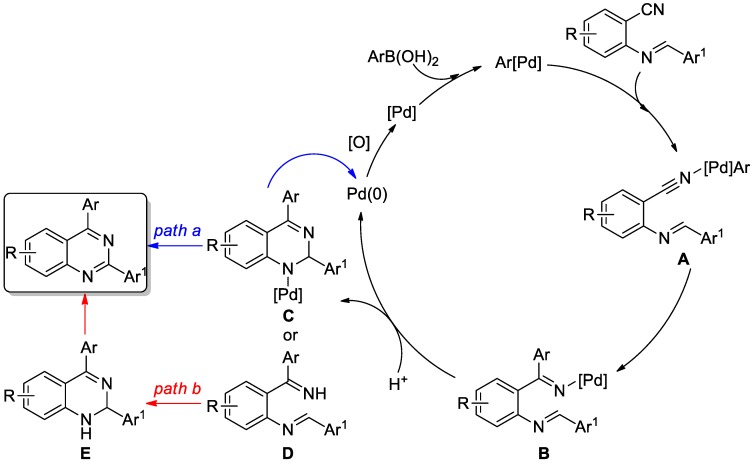
Plausible reaction mechanism for the formation of quinazolines.

On the basis of the above experimental results and relevant reports in the literature, a possible reaction mechanism for the formation of 2,4-diarylquinazolines is illustrated in Scheme 5. The first step may involve transmetalation between the palladium catalyst and arylboronic acid to form the palladium-aryl species, which is followed by the coordination of cyano group affording intermediate **A**. Intramolecular carbopalladation of nitrile gives the corresponding imine palladium intermediate **B**. Next, transformation of the intermediate **B** could proceed by two possible pathways. In path a, the intermediate **B** undergoes an intramolecular cyclization to palladium complex **C**. β-Hydride elimination of the intermediate **C** would yield 2,4-diarylquinazolines and Pd(0) species which could be further oxidized to Pd(II). In path b, protonation of the intermediate **B** by TsOH·H_2_O delivers the imine intermediate **D** and regenerates the palladium catalyst. Finally, intramolecular cyclization of intermediate **D** generates dihydroquinazolines **E,** which after oxidative dehydrogenation delivers 2,4-diarylquinazolines as the desired products.

## 3. Materials and Methods

### 3.1. General Information

Chemicals were received from commercial sources without further purification, or prepared by methods from the literature. ^1^H-NMR (500 MHz) and ^13^C-NMR (125 MHz) spectra were measured on a Bruker spectrometer (Billerica, MA, USA), using CDCl_3_ as the solvent with tetramethylsilane (TMS) as the internal standard at room temperature. Chemical shifts are given in *δ* relative to TMS; the coupling constants *J* are given in Hz. High-resolution mass spectra were recorded on ESI-Q-TOF mass spectrometer (Billerica, MA, USA). Melting points were uncorrected and recorded on a WRS-1B Digital Melting Point Apparatus (Jiapeng, Shanghai, China). All reactions were conducted under air atmosphere. Column chromatography was performed using EM Silica gel 60 (300–400 mesh). The structures of all the title compounds were characterized by ^1^H-NMR and ^13^C-NMR spectra (Supplementary Materials).

### 3.2. General Procedure for the Synthesis of 2,4-Diarylquinazolines

Under air atmosphere, a Teflon-valve-sealed Schlenk tube was charged with arylboronic acid, 2-(benzylideneamino)benzonitriles, Pd(acac)_2_, 5,5′-dimethyl-2,2′-bipyridine (**L2**), TsOH·H_2_O and DMF at room temperature. The reaction mixture was stirred for 10 min at room temperature for proper mixing of the reactants, and then heated at 80 °C with vigorous stirring for 48 h. Afterwards, the mixture was poured into ethyl acetate, which was washed with saturated NaHCO_3_ (2 × 10 mL) and then brine (10 mL). The aqueous layer was extracted with ethyl acetate, the combined organic layers were dried over anhydrous Na_2_SO_4_ and evaporated under vacuum. The residue was purified by flash column chromatography (hexane/ethyl acetate) to afford 2,4-diarylquinazolines.

*2,4-Diphenylquinazoline* (**3a**). Yellow solid; mp 116–117 °C. ^1^H-NMR: δ 8.71 (d, *J* = 8.0 Hz, 2H), 8.20 (d, *J* = 8.0 Hz, 1H), 8.14 (d, *J* = 8.0 Hz, 1H), 7.92–7.88 (m, 3H), 7.64–7.59 (m, 3H), 7.58–7.48 (m, 4H); ^13^C-NMR: δ 168.4, 160.2, 151.9, 138.2, 137.7, 133.6, 130.5, 130.2, 129.9, 129.1, 128.7, 128.5, 127.0, 121.7.

*2-Phenyl-4-(p-tolyl)quinazolines* (**3b**). Yellow solid; mp 125–127 °C. ^1^H-NMR: δ 8.71 (d, *J* = 7.0 Hz, 2H), 8.17 (t, *J* = 9.0 Hz, 2H), 7.88 (m, 1H), 7.81 (d, *J* = 8.0 Hz, 2H), 7.58–7.48 (m, 4H), 7.41 (d, *J* = 7.5 Hz, 2H), 2.51 (s, 3H); ^13^C-NMR: δ 168.4, 160.2, 151.9, 140.2, 138.2, 134.9, 133.5, 130.5, 130.2, 129.3, 129.1, 128.7, 128.5, 127.1, 126.9, 121.7, 21.5.

*2-Phenyl-4-(m-tolyl)quinazolines* (**3c**). Yellow solid; mp 81-83 °C. ^1^H-NMR: δ 8.71 (d, *J* = 7.0 Hz, 2H), 8.18 (d, *J* = 7.5 Hz, 1H), 8.13 (d, *J* = 7.5 Hz, 1H), 7.89 (t, *J* = 7.5 Hz, 1H), 7.71 (s, 1H), 7.66 (d, *J* = 7.5 Hz, 1H), 7.58–7.46 (m, 5H), 7.41 (d, *J* = 7.5 Hz, 1H), 2.52 (s, 3H); ^13^C-NMR: δ 168.7, 160.3, 151.9, 138.4, 138.3, 137.7, 133.5, 130.7, 130.7, 130.5, 129.1, 128.8, 128.5, 128.4, 127.4, 127.1, 126.9, 121.8, 21.5. HRMS (ESI) calcd for C_21_H_17_N_2_ [M + H]^+^: 297.1386, found 297.1392.

*2-Phenyl-4-(o-tolyl)quinazolines* (**3d**). Yellow solid, mp 140–141 °C. ^1^H-NMR: δ 8.68 (d, *J* = 7.0 Hz, 2H), 8.21 (d, *J* = 8.0 Hz, 1H), 7.90 (t, *J* = 7.5 Hz, 1H), 7.70 (d, *J* = 8.5 Hz, 1H), 7.55–7.49 (m, 4H), 7.46 (t, *J* = 7.5 Hz, 1H), 7.41 (d, *J* = 7.5 Hz, 2H), 7.38 (d, *J* = 8.5 Hz, 1H), 2.24 (s, 3H); ^13^C-NMR: δ 170.0, 160.3, 151.3, 138.1, 136.9, 136.5, 133.8, 130.8, 130.6, 129.7, 129.3, 128.9, 128.8, 128.6, 127.1, 127.1, 125.6, 122.7, 20.0. HRMS (ESI) calcd for C_21_H_17_N_2_ [M + H]^+^: 297.1386, found 297.1392.

*4-(4-(tert-Butyl)phenyl)-2-phenylquinazoline* (**3e**). Yellow oil. ^1^H-NMR: δ 8.74 (d, *J* = 7.0 Hz, 2H), 8.18 (m, 2H), 7.90–7.85 (m, 3H), 7.64 (d, *J* = 8.0 Hz, 2H), 7.58–7.50 (m, 4H), 1.45 (s, 9H); ^13^C-NMR: δ 168.3, 160.3, 153.3, 125.0, 138.3, 134.9, 133.4, 130.5, 130.1, 129.1, 128.7, 128.5, 127.2, 126.9, 125.6, 121.8, 34.9, 31.4. HRMS (ESI) calcd for C_24_H_23_N_2_ [M + H]^+^: 339.1856, found 339.1857.

*4-(4-Methoxyphenyl)-2-phenylquinazoline* (**3f**). Yellow solid; mp 120–121 °C. ^1^H-NMR: δ 8.71 (d, *J* = 7.0 Hz, 2H), 8.16 (t, *J* = 9.5 Hz, 2H), 7.90 (d, *J* = 8.5 Hz, 2H), 7.86 (d, *J* = 8.0 Hz, 1H), 7.55–7.50 (m, 4H), 7.12 (d, *J* = 8.5 Hz, 2H), 3.93 (s, 3H); ^13^C-NMR: δ 167.8, 161.3, 160.1, 152.0, 138.3, 133.4, 131.9, 130.5, 130.2, 129.1, 128.7, 128.5, 127.1, 126.9, 121.7, 114.1, 55.5.

*4-(2-Phenylquinazolin-4-yl)phenol* (**3g**). Yellow solid; mp 190–192 °C. ^1^H-NMR: δ 8.69-8.67 (m, 2H), 8.22-8.16 (m, 2H), 7.89 (t, *J* = 7.2 Hz, 1H), 7.83 (d, *J* = 8.5 Hz, 2H), 7.58–7.51 (m, 4H), 7.04 (d, *J* = 8.5 Hz, 2H); ^13^C-NMR: δ 167.9, 160.2, 157.6, 138.1, 133.6, 132.1, 130.6, 130.2, 128.9, 128.7, 128.6, 127.1, 127.0, 121.6, 115.6. HRMS (ESI) calcd for C_20_H_15_N_2_O [M+H]^+^: 299.1179, found 299.1181.

*4-(4-Fluorophenyl)-2-phenylquinazoline* (**3h**). Yellow solid; mp 132-134 °C. ^1^H-NMR: δ 8.69 (d, *J* = 7.5 Hz, 2H), 8.19 (d, *J* = 8.5 Hz, 2H), 8.11 (d, *J* = 8.5 Hz, 1H), 7.95–7.88 (m, 3H), 7.60–7.49 (m, 4H), 7.30 (t, *J* = 8.5 Hz, 2H); ^13^C-NMR: δ 167.2, 165.0, 163.0, 160.2, 152.0, 138.1, 133.8, 133.8, 133.7, 133.2, 133.2, 130.6, 129.3, 128.7, 128.6, 127.2, 126.7, 121.6, 115.8, 115.6.

*4-(4-Chlorophenyl)-2-phenylquinazoline* (**3i**). Yellow solid; mp 136-137 °C. ^1^H-NMR: δ 8.70 (d, *J* = 7.0 Hz, 2H), 8.24 (d, *J* = 8.0 Hz, 1H), 8.09 (d, *J* = 8.0 Hz, 1H), 7.92 (t, *J* = 7.5 Hz, 1H), 7.85 (d, *J* = 8.0 Hz, 2H), 7.58 (t, *J* = 7.5 Hz, 3H), 7.56–7.50 (m, 3H); ^13^C-NMR: δ 167.2, 160.2, 152.0, 137.9, 136.4, 136.1, 133.8, 131.5, 130.7, 129.3, 128.9, 128.7, 128.6, 127.3, 126.6, 121.5. HRMS (ESI) calcd for C_20_H_14_ClN_2_ [M + H]^+^: 317.0840, found 317.0844.

*4-(4-Bromophenyl)-2-phenylquinazoline* (**3j**). Yellow solid; mp 146–148 °C. ^1^H-NMR: δ 8.68 (d, *J* = 7.0 Hz, 2H), 8.17 (d, *J* = 9.0 Hz, 1H), 8.07 (d, *J* = 8.5 Hz, 1H), 7.90 (t, *J* = 7.5 Hz, 1H), 7.80 (d, *J* = 8.0 Hz, 2H), 7.54 (d, *J* = 8.0 Hz, 2H), 7.59–7.50 (m, 4H); ^13^C-NMR: δ 166.2, 159.2, 150.9, 136.9, 135.5, 132.8, 130.8, 130.7, 129.7, 128.2, 127.7, 127.6, 126.2, 125.5, 123.7, 120.4.

*4-([1,1′-Biphenyl]-4-yl)-2-phenylquinazoline* (**3l**). Yellow solid; mp 217–218 °C. ^1^H-NMR: δ 8.73 (d, *J* = 7.5 Hz, 2H), 8.22 (d, *J* = 8.0 Hz, 2H), 8.00 (d, *J* = 7.5 Hz, 2H), 7.91 (t, *J* = 7.5 Hz, 1H), 7.84 (d, *J* = 7.5 Hz, 2H), 7.72 (d, *J* = 7.5 Hz, 2H), 7.60–7.50 (m, 6H), 7.43 (t, *J* = 7.5 Hz, 1H); ^13^C-NMR: δ 168.0, 160.3, 152.0, 142.9, 140.4, 138.1, 136.6, 133.6, 130.7, 130.6, 129.2, 129.0, 128.8, 128.6, 127.9, 127.4, 127.3, 127.1, 127.0, 121.7. HRMS (ESI) calcd for C_26_H_19_N_2_ [M + H]^+^: 359.1543, found 359.1541.

*4-(Naphthalen-1-yl)-2-phenylquinazoline* (**3m**). Yellow solid; mp 178–180 °C. ^1^H-NMR: δ 8.72-8.70 (m, 2H), 8.23 (d, *J* = 8.5 Hz, 1H), 8.08–8.06 (m, 1H), 8.00 (d, *J* = 8.5 Hz, 1H), 7.91–7.88 (m, 1H), 7.69–7.64 (m, 4H), 7.54–7.51 (m, 4H), 7.44–7.40 (m, 2H); ^13^C-NMR: δ 169.0, 160.5, 151.7, 138.3, 135.0, 133.9, 133.8, 131.8, 130.6, 129.8, 129.1, 128.9, 128.6, 128.4, 128.0, 127.3, 127.0, 126.7, 126.3, 125.8, 125.1, 123.4.

*4-(Naphthalen-2-yl)-2-phenylquinazoline* (**3n**). Yellow solid; mp 161–162 °C. ^1^H-NMR: δ 8.75 (d, *J* = 7.0 Hz, 2H), 8.37 (s, 1H), 8.20 (t, *J* = 11.0 Hz, 2H), 8.08 (d, *J* = 8.0 Hz, 1H), 8.05–7.95 (m, 3H), 7.91 (t, *J* = 7.0 Hz, 1H), 7.65–7.60 (m, 2H), 7.59–7.49 (m, 4H); ^13^C-NMR: δ 168.3, 160.3, 152.0, 138.2, 135.1, 134.0, 133.6, 133.0, 130.6, 130.3, 129.2, 128.7, 128.6, 128.4, 127.8, 127.3, 127.3, 127.1, 127.1, 126.7, 121.9. HRMS (ESI) calcd for C_24_H_17_N_2_ [M + H]^+^: 333.1386, found 333.1387.

*4-Phenyl-2-(p-tolyl)quinazoline* (**3o**). Yellow solid; mp 162–165 °C. ^1^H-NMR: δ 8.60 (d, *J* = 8.0 Hz, 2H), 8.20 (s, 1H), 8.12 (d, *J* = 8.0 Hz, 1H), 7.90–7.87 (m, 3H), 7.61–7.59 (m, 3H), 7.54 (t, *J* = 8.0 Hz, 1H),7.34 (t, *J* = 7.5 Hz, 2H), 2.44 (s, 3H); ^13^C-NMR (125 MHz, CDCl_3_): δ 168.4, 160.3, 151.8, 140.8, 137.8, 135.4, 133.5, 130.2, 129.5, 129.3, 129.0, 128.7, 128.5, 127.0, 126.8, 121.6, 21.5.

*4-Phenyl-2-(m-tolyl)quinazoline* (**3p**). Yellow solid; mp 112–114 °C. ^1^H-NMR: δ 8.50 (d, *J* = 8.5 Hz, 2H), 8.17 (d, *J* = 8.5 Hz, 1H), 8.13 (d, *J* = 8.5 Hz, 1H), 7.90–7.87 (m, 3H), 7.63–7.58 (m, 3H), 7.55 (t, *J* = 8.5 Hz, 1H), 7.43 (t, *J* = 7.5 Hz, 1H), 7.32 (d, *J* = 7.5 Hz, 1H), 2.50 (s, 3H); ^13^C-NMR: δ 168.3, 160.4, 152.0, 138.1, 137.7, 133.5, 131.4, 130.2, 129.9, 129.2, 129.1, 128.6, 128.5, 127.0, 126.9, 126.0, 121.7, 22.6.

*4-Phenyl-2-(o-tolyl)quinazoline* (**3q**). Yellow solid; mp 73–75 °C. ^1^H-NMR: δ 8.18 (t, *J* = 8.0 Hz, 2H), 7.98 (d, *J* = 7.5 Hz, 1H), 7.92 (t, *J* = 8.0 Hz, 1H), 7.87 (d, *J* = 7.5 Hz, 2H), 7.63–7.55 (m, 4H), 7.39–7.31 (m, 3H), 2.67 (s, 3H); ^13^C-NMR: δ 168.1, 163.4, 151.6, 138.7, 137.5, 137.5, 133.6, 131.3, 130.8, 130.2, 129.9, 129.3, 129.0, 128.6, 127.3, 127.0, 126.0, 121.0, 21.3.

*2-(4-Methoxyphenyl)-4-phenylquinazoline* (**3r**). Yellow solid; mp 159–160 °C. ^1^H-NMR: δ 8.67 (d, *J* = 8.5 Hz, 2H), 8.14 (d, *J* = 8.0 Hz, 1H), 8.10 (d, *J* = 8.5 Hz, 1H), 7.89–7.85 (m, 3H), 7.60 (t, *J* = 9.0 Hz, 3H), 7.51 (t, *J* = 8.0 Hz, 1H), 7.04 (d, *J* = 9.0 Hz, 2H), 3.90 (s, 3H); ^13^C-NMR: δ 168.3, 161.9, 160.0, 151.9, 147.2, 137.8, 133.6, 130.4, 130.2, 129.9, 128.8, 128.5, 127.0, 126.6, 121.4, 113.96, 55.4.

*2-(4-Nitrophenyl)-4-phenylquinazoline* (**3s**). Yellow solid; mp 207–209 °C. ^1^H-NMR: δ 8.89 (d, *J* = 9.0 Hz, 2H), 8.36 (d, *J* = 8.5 Hz, 2H), 8.22 (d, *J* = 8.5 Hz, 1H), 8.18 (d, *J* = 8.5 Hz, 1H), 7.96 (t, *J* = 7.5 Hz, 1H), 7.92–7.86 (m, 2H), 7.67–7.60 (m, 4H); ^13^C-NMR: δ 168.9, 158.0, 151.7, 149.3, 144.0, 137.2, 134.1, 130.3, 130.2, 129.6, 129.3, 128.7, 128.1, 127.2, 123.7, 122.0.

*2-(4-Fluorophenyl)-4-phenylquinazoline* (**3t**). Yellow solid; mp 144–145 °C. ^1^H-NMR: δ 8.71 (t, *J* = 8.0 Hz, 2H), 8.17–8.10 (m, 2H), 7.92–7.85 (m, 3H), 7.60 (s, 3H), 7.55 (t, *J* = 7.5 Hz, 1H), 7.20 (t, *J* = 8.0 Hz, 2H); ^13^C-NMR: δ 168.5, 165.7, 163.7, 159.3, 151.9, 137.6, 134.3, 133.7, 130.9, 130.8, 130.2, 130.0, 129.0, 128.6, 127.1, 121.6, 115.5, 115.4.

*2-(4-Chlorophenyl)-4-phenylquinazoline* (**3u**). Yellow solid; mp 180–181 °C. ^1^H-NMR: δ 8.66 (d, *J* = 8.0 Hz, 2H), 8.15 (t, *J* = 8.5 Hz, 2H), 7.92–7.87 (m, 3H), 7.62–7.56 (m, 4H), 7.49 (d, *J* = 8.5 Hz, 2H); ^13^C-NMR: δ 168.8, 159.1, 151.5, 137.5, 137.0, 136.3, 133.9, 130.3, 130.2, 128.8, 128.6, 127.4, 127.1, 121.7.

*2-(4-Bromophenyl)-4-phenylquinazoline* (**3v**). Yellow solid; mp 185–186 °C. ^1^H-NMR: δ 8.59 (d, *J* = 8.5 Hz, 2H), 8.16 (d, *J* = 8.0 Hz, 1H), 8.14 (d, *J* = 8.5 Hz, 1H), 7.93–7.85 (m, 3H), 7.65 (d, *J* = 8.5 Hz, 2H), 7.60 (t, *J* = 8.0 Hz, 3H), 7.57 (t, *J* = 8.0 Hz, 1H); ^13^C-NMR: δ 168.5, 159.3, 151.8, 137.5, 137.1, 133.8, 131.7, 130.3, 130.2, 130.1, 129.1, 128.6, 127.3, 127.1, 125.4, 121.8.

*2-(Naphthalen-1-yl)-4-phenylquinazoline* (**3w**). Yellow solid; mp 169–170 °C. ^1^H-NMR: δ 8.22 (d, *J* = 8.5 Hz, 1H), 8.27–8.21 (m, 3H), 8.00–8.79 (m, 5H), 7.66–7.52 (m, 7H); ^13^C-NMR: δ 168.5, 162.8, 151.7, 137.5, 136.5, 134.3, 133.8, 131.4, 130.3, 130.2, 130.0, 129.7, 129.1, 128.6, 128.5, 127.5, 127.1, 126.7, 126.1, 125.8, 125.3, 121.3.

*4-Phenyl-2-(thiophen-2-yl)quinazoline* (**3x**). Yellow solid; mp 136–137 °C. ^1^H-NMR: δ 8.22 (d, *J* = 3.0 Hz, 1H), 8.10–8.07 (m, 2H), 7.88–7.83 (m, 3H), 7.60–7.59 (m, 3H), 7.52–7.48 (m, 2H), 7.20–7.18 (m, 1H); ^13^C-NMR: δ 168.5, 157.2, 151.8, 144.1, 137.3, 133.8, 130.2, 130.1, 130.0, 129.9, 129.4, 128.6, 128.3, 127.2, 126.8, 121.5.

*4-(4-Methoxyphenyl)-2-(thiophen-2-yl)quinazoline* (**3y**). Yellow solid; mp 143–144 °C. ^1^H-NMR: δ 8.22 (s, 1H), 8.14-8.08 (m, 2H), 7.88–7.83 (m, 3H), 7.52–7.49 (m, 2H), 7.19–7.18 (m, 1H), 7.11 (d, *J* = 8.5 Hz, 2H), 3.93 (s, 3H); ^13^C-NMR: δ 167.9, 164.1, 157.1, 151.8, 144.2, 133.6, 131.9, 129.9, 129.8, 129.4, 128.6, 128.2, 127.2, 126.6, 121.5, 114.1, 55.5. HRMS (ESI) calcd for C_19_H_15_N_2_OS [M + H]^+^: 319.0900, found 319.0905.

*7-Methyl-2,4-diphenylquinazoline* (**3z**). Yellow solid; mp 150–152 °C. ^1^H-NMR: δ 8.69 (d, *J* = 6.5 Hz, 2H), 8.13 (d, *J* = 6.5 Hz, 1H), 7.90–7.88 (m, 3H), 7.74 (d, *J* = 7.5 Hz, 1H), 7.63–7.60 (m, 3H), 7.55–7.50 (m, 3H), 2.52 (s, 3H); ^13^C-NMR: δ 168.0, 159.4, 150.0, 137.8, 137.4, 136.0, 130.6, 130.2, 129.9, 128.8, 128.7, 128.5, 128.4, 125.7, 121.7, 21.9.

*8-Chloro-2,4-diphenylquinazoline* (**4a**). White solid; mp 144–146 °C. ^1^H-NMR: δ 8.79–8.77 (m, 2H), 8.06-8.04 (m, 1H), 7.99 (d, *J* = 7.5 Hz, 1H), 7.89–7.86 (m, 2H), 7.62–7.60 (m, 3H), 7.55–7.53 (m, 2H), 7.46 (t, *J* = 8.2 Hz, 1H), 7.37–7.35 (m, 1H); ^13^C-NMR: δ 168.9, 148.6, 137.9, 137.4, 133.8, 133.4, 130.9, 130.2, 130.1, 129.0, 128.6, 127.2, 126.5, 126.0, 123.0. HRMS (ESI) calcd for C_20_H_14_ClN_2_ [M + H]^+^: 317.0840, found 317.0844.

*6,7-Dimethoxy-2,4-diphenylquinazoline* (**4b**). Yellow solid; mp 176–178 °C. ^1^H-NMR: δ 8.64 (d, *J* = 7.0 Hz, 2H), 7.88 (d, *J* = 7.0 Hz, 2H), 7.60–7.55 (m, 3H), 7.53–7.47 (m, 3H), 7.44 (s, 1H), 7.33 (s, 1H), 4.07 (s, 3H), 3.88 (s, 3H); ^13^C-NMR: δ 165.1, 159.3, 155.7, 150.0, 149.9, 138.5, 138.3, 130.0, 129.8, 129.6, 128.6, 128.5, 128.3, 117.1, 107.4, 104.2, 56.4, 56.1.

*6,7-Dimethoxy-2-phenyl-4-(4-(trifluoromethyl)phenyl)quinazoline* (**4c**). Yellow solid; mp 179–180 °C. ^1^H-NMR: δ 8.60 (d, *J* = 7.0 Hz, 2H), 7.99 (d, *J* = 8.0 Hz, 2H), 7.86 (d, *J* = 8.0 Hz, 2H), 7.53–7.47 (m, 4H), 7.24 (s, 1H), 4.12 (s, 3H), 3.92 (s, 3H); ^13^C-NMR: δ 163.6, 159.4, 156.0, 150.5, 150.1, 141.8, 138.2, 130.2, 130.1, 128.5, 128.3, 125.7, 125.6, 117.0, 107.6, 103.4, 56.5, 56.2. HRMS (ESI) calcd for C_23_H_17_F_3_N_2_O_2_Na [M + Na]^+^: 433.1140, found 433.1144.

*4-(4-Isopropylphenyl)-6,7-dimethoxy-2-phenylquinazoline* (**4d**): Yellow solid; mp 169-170 °C. ^1^H-NMR: δ 8.63 (d, *J* = 7.0 Hz, 2H), 7.85 (d, *J* = 8.5 Hz, 2H), 7.52–7.43 (m, 7H), 4.11 (s, 3H), 3.94 (s, 3H), 3.06–3.02 (m, 1H), 1.35 (d, *J* = 6.5 Hz, 6H); ^13^C-NMR: δ 165.2, 159.3, 155.7, 150.7, 150.0, 149.8, 138.5, 135.8, 130.0, 129.9, 128.4, 128.3, 126.8, 117.1, 107.4, 104.5, 56.4, 56.2, 34.1, 23.9. HRMS (ESI) calcd for C_25_H_25_N_2_O_2_ [M + H]^+^: 385.1911, found 385.1917.

*4-(3,5-Dimethylphenyl)-6,7-dimethoxy-2-phenylquinazoline* (**4e**). Yellow solid; mp 176–177 °C. ^1^H-NMR: 8.63 (d, *J* = 7.0 Hz, 2H), 7.53–7.45 (m, 6H), 7.31 (s, 1H), 7.20 (s, 1H), 4.10 (s, 3H), 3.91 (s, 3H) 2.46 (s, 6H); ^13^C-NMR: δ 165.7, 159.4, 155.7, 150.0, 149.9, 138.7, 138.3, 138.2, 131.3, 130.0, 128.4, 128.3, 127.6, 117.3, 107.4, 104.6, 56.4, 56.1, 21.4. HRMS (ESI) calcd for C_24_H_23_N_2_O_2_ [M+H]^+^: 371.1754, found 371.1755.

*5-Fluoro-2,4-diphenylquinazoline* (**4f**). Yellow solid; mp 177–178 °C. ^1^H-NMR: δ 8.69–8.68 (m, 2H), 8.13–8.01 (m, 2H), 7.88–7.82 (m, 3H), 7.63–7.62 (m, 3H), 7.54–7.52 (m, 3H); ^13^C-NMR: δ 167.6, 160.5, 150.5, 137.8, 137.2, 134.5, 132.7, 130.9, 130.8, 130.3, 130.1, 128.9, 128.8, 128.6, 125.8, 122.2.

*6-Chloro-2,4-diphenylquinazoline* (**4g**). Yellow solid; mp 194-195 °C. ^1^H-NMR: δ 8.69 (d, *J* = 6.5 Hz, 2H), 8.10 (d, *J* = 10.0 Hz, 2H), 7.87-7.86 (m, 2H), 7.81 (d, *J* = 8.5 Hz, 1H), 7.62 (s, 3H), 7.53 (d, *J* = 6.0 Hz, 3H); ^13^C-NMR: δ 167.6, 160.5, 150.5, 137.8, 137.2, 134.5, 132.6, 130.9, 130.8, 130.2, 130.1, 128.7, 128.6, 125.8, 122.2.

*6-Bromo-2,4-diphenylquinazoline* (**4h**). Yellow solid; mp 204–205 °C. ^1^H-NMR: δ 8.68 (d, *J* = 7.5 Hz, 2H), 8.27 (d, *J* = 2.0 Hz, 1H), 8.07 (d, *J* = 9.0 Hz, 1H), 7.96 (d, *J* = 8.5 Hz, 1H), 7.88–7.86 (m, 2H), 7.63–7.62 (m, 3H), 7.53 (d, *J* = 6.0 Hz, 3H); ^13^C-NMR: δ 167.6, 160.5, 150.6, 137.7, 137.1, 137.0, 130.9, 130.8, 130.3, 130.1, 129.1, 128.8, 128.7, 128.6, 122.7, 120.7.

*6-Nitro-2,4-diphenylquinazoline* (**4i**). Yellow solid; mp 211–213 °C. ^1^H-NMR: δ 9.07 (d, *J* = 2.3 Hz, 1H), 8.75–8.73 (m, 2H), 8.67–8.64 (m, 1H), 8.27 (d, *J* = 9.3 Hz, 1H), 7.93–7.91 (m, 2H), 7.69–7.68 (m, 3H), 7.57–7.56 (m, 3H); ^13^C-NMR: δ 170.5, 162.9, 154.5, 145.5, 137.1, 136.4, 131.7, 131.0, 130.9, 130.3, 129.2, 129.1, 128.7, 127.0, 124.3, 120.5. 

*2,4-Diphenyl-1,2-dihydroquinazoline* (**5a**). Yellow solid; mp 57-59 °C. ^1^H-NMR: δ7.66-7.62 (m, 4H), 7.49–7.37 (m, 6H), 7.32 (d, *J* = 8.3 Hz, 1H), 7.23 (d, *J* = 7.7 Hz, 1H), 6.78 (t, *J* = 7.5 Hz, 1H), 6.72 (d, *J* = 8.0 Hz, 1H), 5.99 (s, 1H), 4.38 (s, 1H); ^13^C-NMR: δ 165.7, 146.9, 142.6, 138.1, 132.8, 129.4, 129.2, 128.9, 128.7, 128.4, 128.1, 127.3, 118.2, 117.9, 114.3, 72.6.

## 4. Conclusions

In summary, we have developed a new strategy for the synthesis of 2,4-diarylquinazolines in moderate to good yields via the palladium-catalyzed tandem addition/cyclization of 2-(benzylidene-amino)bnenzonitriles with arylboronic acids. This catalytic system tolerates a broad range of substrates and functional groups. Further efforts to extend this chemistry to the preparation of other useful heterocyclic compounds are currently underway in our laboratories.

## Figures and Tables

**Table 1:**
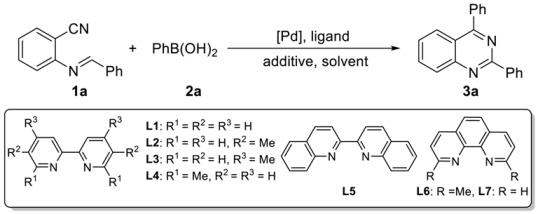
Optimization of the reaction conditions ^a^.

Entry	Pd Catalyst	Ligand	Additive	Solvent	Yield ^b^ (%)
1	Pd(PPh_3_)_4_	**L1**	TfOH	THF/H_2_O ^c^	trace
2	PdCl_2_	**L1**	TfOH	THF/H_2_O	15
3	Pd(OAc)_2_	**L1**	TfOH	THF/H_2_O	17
4	Pd_2_(dba)_3_	**L1**	TfOH	THF/H_2_O	14
5	Pd(CF_3_CO_2_)_2_	**L1**	TfOH	THF/H_2_O	19
6	Pd(acac)_2_	**L1**	TfOH	THF/H_2_O	27
7	Pd(acac)_2_	**L2**	TfOH	THF/H_2_O	45
8	Pd(acac)_2_	**L3**	TfOH	THF/H_2_O	22
9	Pd(acac)_2_	**L4**	TfOH	THF/H_2_O	trace
10	Pd(acac)_2_	**L5**	TfOH	THF/H_2_O	trace
11	Pd(acac)_2_	**L6**	TfOH	THF/H_2_O	trace
12	Pd(acac)_2_	**L7**	TfOH	THF/H_2_O	18
13	Pd(acac)_2_	**L2**	TfOH	H_2_O	29
14	Pd(acac)_2_	**L2**	TfOH	1,4-dioxane	11
15	Pd(acac)_2_	**L2**	TfOH	toluene	13
16	Pd(acac)_2_	**L2**	TfOH	DMA	31
17	Pd(acac)_2_	**L2**	TfOH	DMF	57
18	Pd(acac)_2_	**L2**	AcOH	DMF	trace
19	Pd(acac)_2_	**L2**	TFA	DMF	41
20	Pd(acac)_2_	**L2**	CSA	DMF	47
21	Pd(acac)_2_	**L2**	TsOH·H_2_O	DMF	81
22	Pd(acac)_2_	**L2**	HCl	DMF	0
23		**L2**	TsOH·H_2_O	DMF	0
24	Pd(acac)_2_	**L2**		DMF	0

^a^ Reaction conditions: **1a** (0.3 mmol), **2a** (0.6 mmol), Pd catalyst (10 mol%), ligand (20 mol%), additive (4 equiv.), KF (2 equiv.), solvent (2 mL), 80 °C, 48 h, air. ^b^ Isolated yield. ^c^ THF/H_2_O (1 mL/1 mL).

## Supplementary Materials

The following are available online. Figures S1–S35.

## References

[1] Fleming F.F., Wang Q. (2003). Unsaturated nitriles:  conjugate additions of carbon nucleophiles to a recalcitrant class of acceptors. Chem. Rev..

[2] Kukushkin V.Y., Pombeiro A.J.L. (2002). Additions to metal-activated organonitriles. Chem. Rev..

[3] Enders D., Shilvock J.P. (2000). Some recent applications of α-amino nitrile chemistry. Chem. Soc. Rev..

[4] Rach S.F., Kühn F.E. (2009). Nitrile ligated transition metal complexes with weakly coordinating counteranions and their catalytic applications. Chem. Rev..

[5] Larock R.C., Tian Q.P., Pletnev A.A. (1999). Carbocycle synthesis via carbopalladation of nitriles. J. Am. Chem. Soc..

[6] Zhou C., Larock R.C. (2004). Synthesis of aryl ketones by the Pd-catalyzed C−H activation of arenes and intermolecular carbopalladation of nitriles. J. Am. Chem. Soc..

[7] Zhou C., Larock R.C. (2006). Synthesis of aryl ketones or ketimines by palladium-catalyzed arene C−H addition to nitriles. J. Org. Chem..

[8] Zhao B., Lu X. (2006). Palladium(II)-catalyzed addition of arylboronic acid to nitriles. Tetrahedron Lett..

[9] Wong Y.C., Parthasarathy K., Cheng C.H. (2010). Direct synthesis of arylketones by nickel-catalyzed addition of arylboronic acids to nitriles. Org. Lett..

[10] Demir S., Yiğit M., Özdemir I. (2013). Synthesis of rhodium complexes derived from benzimidazolin-2-ylidene ligands and first used for the addition of arylboron to benzonitriles. J. Organomet. Chem..

[11] Yousuf M., Das T., Adhikari S. (2015). Palladium catalyzed decarboxylative acylation of arylboronic acid with ethyl cyanoacetate as a new acylating agent: Synthesis of alkyl aryl ketones. New J. Chem..

[12] Wang X., Liu M., Xu L., Wang Q., Chen J., Ding J., Wu H. (2013). Palladium-catalyzed addition of potassium aryltrifluoroborates to aliphatic nitriles: Synthesis of alkyl aryl ketones, diketone compounds, and 2-arylbenzo[*b*]furans. J. Org. Chem..

[13] Chen J., Ye L., Su W. (2014). Palladium-catalyzed direct addition of arylboronic acids to 2-aminobenzonitrile derivatives: Synthesis, biological evaluation and in silico analysis of 2-aminobenzophenones, 7-benzoyl-2-oxoindolines, and 7-benzoylindoles. Org. Biomol. Chem..

[14] Yu S., Qi L., Hu K., Gong J., Cheng T., Wang Q., Chen J., Wu H. (2017). The Development of a palladium-catalyzed tandem addition/cyclization for the construction of indole skeletons. J. Org. Chem..

[15] Skillinghaug B., Skçld C., Rydfjord J., Svensson F., Behrends M., Sävmarker J.S., Sjçberg P.J.R., Larhed M. (2014). Palladium(II)-catalyzed desulfitative synthesis of aryl ketones from sodium arylsulfinates and nitriles: Scope, limitations, and mechanistic studies. J. Org. Chem..

[16] Skillinghaug B., Rydfjord J., Sävmarker J.S., Larhed M. (2016). Microwave heated continuous flow palladium(II)-catalyzed desulfitative synthesis of aryl ketones. Org. Process Res. Dev..

[17] Behrends M., Sävmarker J., Sjöberg P.J.R., Larhed M. (2011). Microwave-assisted palladium(II)-catalyzed synthesis of aryl ketones from aryl sulfinates and direct ESI-MS studies thereof. ACS Catal..

[18] Miao T., Wang G. (2011). Synthesis of ketones by palladium-catalysed desulfitative reaction of arylsulfinic acids with nitriles. Chem. Commun..

[19] Hsieh J.-C., Chen Y.-C., Cheng A.-Y., Tseng H.C. (2012). Nickel-catalyzed intermolecular insertion of aryl iodides to nitriles: A novel method to synthesize arylketones. Org. Lett..

[20] Wan J.-C., Huang J.-M., Jhan Y.-H., Hsieh J.-C. (2013). Novel syntheses of fluorenones via nitrile-directed palladium-catalyzed C–H and dual C–H bond activation. Org. Lett..

[21] Lindh J., Sjçerg P., Larhed M. (2010). Synthesis of aryl ketones by palladium(II)-catalyzed decarboxylative addition of benzoic acids to nitriles. Angew. Chem. Int. Ed..

[22] Cheng K., Wang G., Meng M., Qi C. (2017). Acid-promoted denitrogenative Pd-catalyzed addition of arylhydrazines with nitriles at room temperature. Org. Chem. Front..

[23] Meng M., Yang L., Cheng K., Qi C. (2018). Pd(II)-catalyzed denitrogenative and desulfinative addition of arylsulfonyl hydrazides with nitriles. J. Org. Chem..

[24] Qi L., Hu k., Yu s., Zhu J., Cheng T., Wang X., Chen J., Wu H. (2017). Tandem addition/cyclization for access to isoquinolines and isoquinolones via catalytic carbopalladation of nitriles. Org. Lett..

[25] Hu K., Qi L., Yu S., Cheng T., Wang X., Li Z., Xia Y., Chen J., Wu H. (2017). Efficient synthesis of isoquinolines in water by a Pd-catalyzed tandem reaction of functionalized alkylnitriles with arylboronic acids. Green Chem..

[26] Foster B.A., Coffey H.A., Mornin M.J., Rastinejad F. (1999). Pharmacological rescue of mutant p53 conformation and function. Science.

[27] Bghrmann M., Hardick J., Weisner J., Quambusch L., Rauh D. (2017). Covalent lipid pocket ligands targeting p38α MAPK mutants. Angew. Chem. Int. Ed..

[28] Smutny T., Nova A., Drechslerová M., Carazo A., Hyrsova L., Hrušková Z.R., Kuneš J., Pour M., Špulák M., Pavek P. (2016). 2-(3-Methoxyphenyl)quinazoline derivatives: A new class of direct constitutive androstane receptor (CAR) agonists. J. Med. Chem..

[29] Rosse G. (2016). Quinazoline carboxamides as selective antagonists of adenosine 2A receptor. ACS Med. Chem. Lett..

[30] Shagufta, Ahmad I. (2017). An insight into the therapeutic potential of quinazoline derivatives as anticancer agents. Med. Chem. Commun..

[31] Zhang Y.L., Sheets M.R., Raja E.K., Boblak K.N., Klumpp D.A. (2011). Superacid-promoted additions involving vinyl-substituted pyrimidines, quinoxalines, and quinazolines: Mechanisms correlated to charge distributions. J. Am. Chem. Soc..

[32] Achelle S., Rodriguez-Lopez J., Robin-le Guen F. (2014). Synthesis and photophysical studies of a series of quinazoline chromophores. J. Org. Chem..

[33] Liu D., Zhang Z., Zhang H., Wang Y. (2013). A novel approach towards white photoluminescence and electroluminescence by controlled protonation of a blue fluorophore. Chem. Commun..

[34] Connolly D.J., Lacey P.M., McCarthy M., Saunders C.P., Carroll A.M., Goddard R., Guiry P.J. (2004). Preparation and resolution of a modular class of axially chiral quinazoline-containing ligands and their application in asymmetric rhodium-catalyzed olefin hydroboration. J. Org. Chem..

[35] Kikelj D. (2014). Product class 13: Quinazolines. Science of Synthesis.

[36] Connolly D.J., Cusack D., O’Sullivan T.P., Guiry P.J. (2015). Synthesis of quinazolinones and quinazolines. Tetrahedron.

[37] Khan I., Ibrar A., Abbas N., Saeed A. (2014). Recent advances in the structural library of functionalized quinazoline and quinazolinone scaffolds: Synthetic approaches and multifarious applications. Eur. J. Med. Chem..

[38] Strekowski L., Cegla M.T., Harden D.B., Mokrosz J.L., Mokrosz M.J. (1988). Regioselective additions of grignard and lithium reagents to 2-[(benzylidene)aminoi]benzonitrile and 2-[(diphenylmethylene)amino]benzonitrile. Tetrahedron Lett..

[39] Su X., Chen C., Wang Y., Chen J., Lou Z., Li M. (2013). One-pot synthesis of quinazoline derivatives via [2+2+2] cascade annulation of diaryliodonium salts and two nitriles. Chem. Commun..

[40] Ramanathan M., Liu S.-T. (2017). Preparation of quinazolines via a 2+2+2 annulation from aryldiazonium salts and nitriles. J. Org. Chem..

[41] Zhang Y., Shao Y., Gong J., Hu K., Cheng T., Chen J. (2018). Palladium-catalyzed tandem reaction of quinazolinone-based nitriles with arylboronic acids: Synthesis of 2-(4-arylquinazolin-2-yl)anilines. Adv. Synth. Catal..

[42] Zhu J., Shao Y., Hu K., Qi L., Cheng T., Chen J. (2018). Pd-Catalyzed tandem reaction of *N*-(2-cyanoaryl)benzamides with arylboronic acids: Synthesis of quinazolines. Org. Biomol. Chem..

