# Supplementary material for: Efficient Tandem Addition/Cyclization for Access to 2,4-Diarylquinazolines via Catalytic Carbopalladation of Nitriles
Source: Molecules. 2019 Jan 28;24(3):463. doi: 10.3390/molecules24030463 (PMC6384746; doi:10.3390/molecules24030463)
Supplement: Supplementary file 1 [file molecules-24-00463-s001.pdf]

Supporting Information

Efficient Tandem Addition/Cyclization for Access to 2,4-Diarylquinazolines via Catalytic Carbopalladation of Nitriles

Julin Gong, Kun Hu, Yetong Zhang, Yinlin Shao*, Tianxing Cheng, Maolin Hu* and Jiuxi Chen

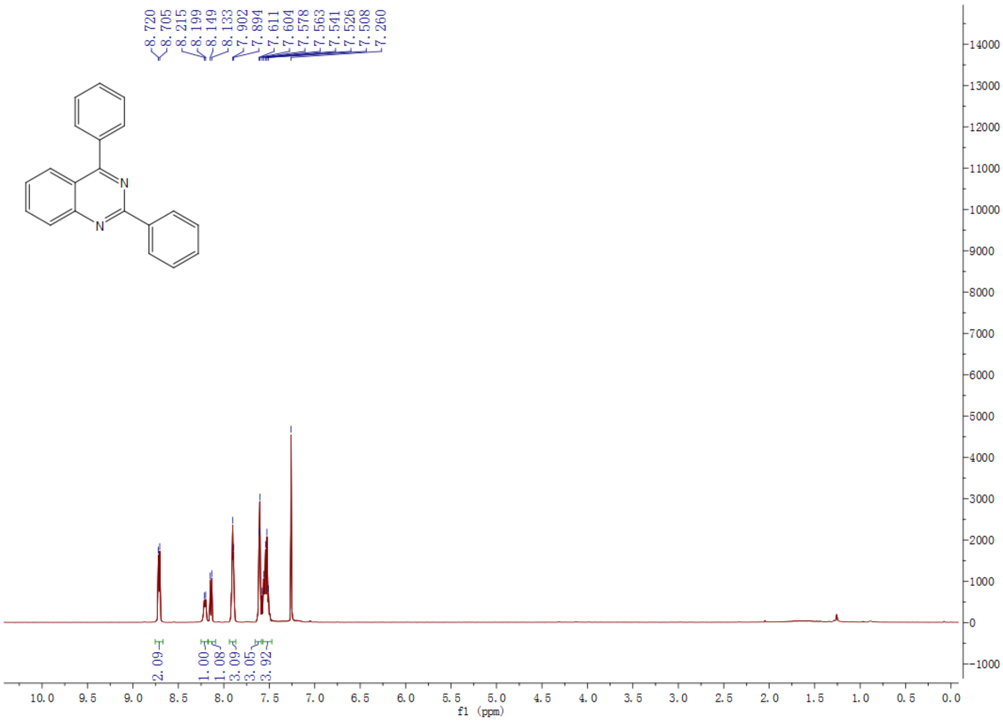

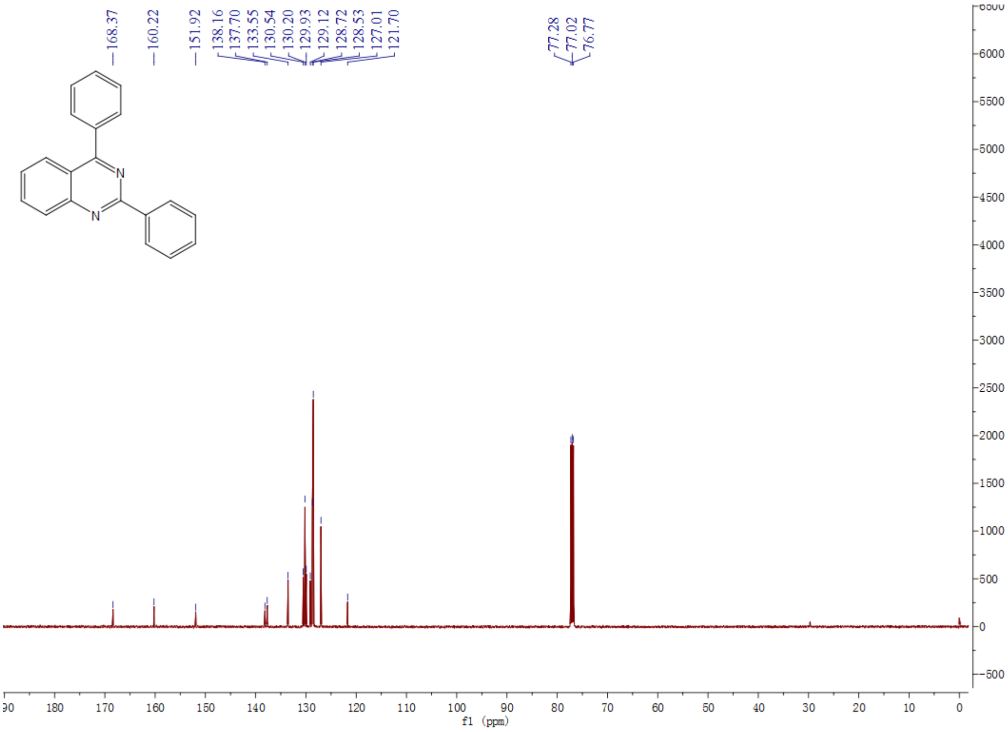

Figure S1. ^{1}H NMR of **3a** (500 MHz, $CDCl_3$) and ^{13}C NMR of **3a** (125 MHz, $CDCl_3$).

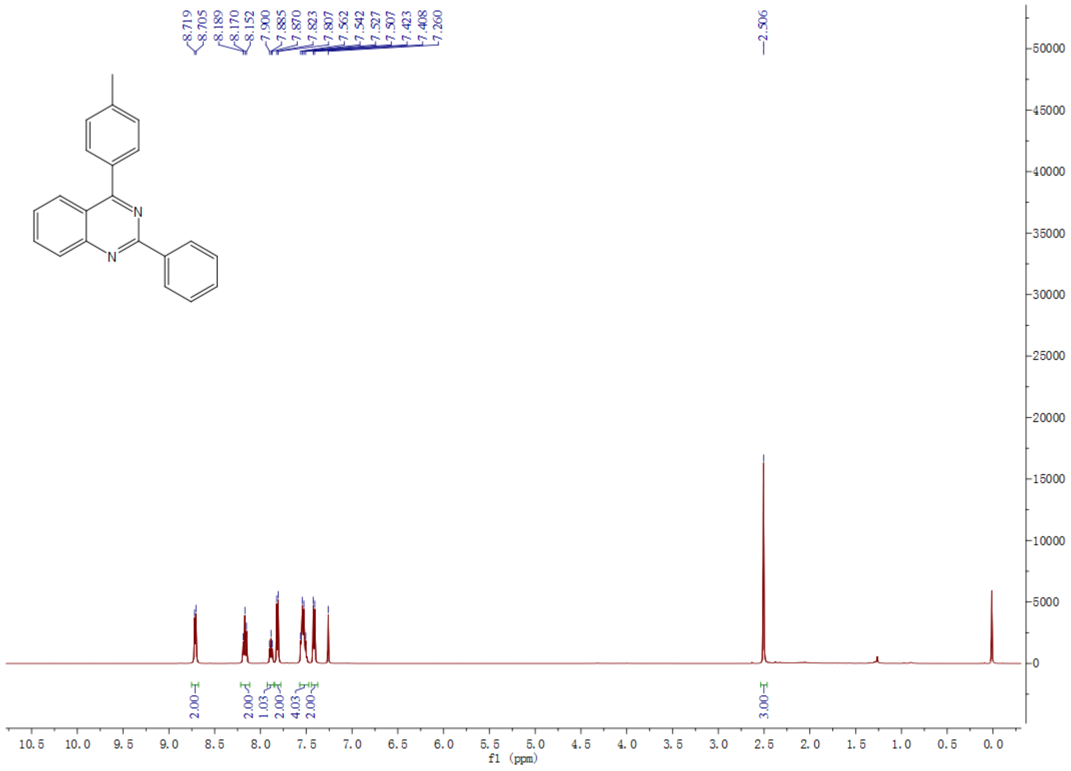

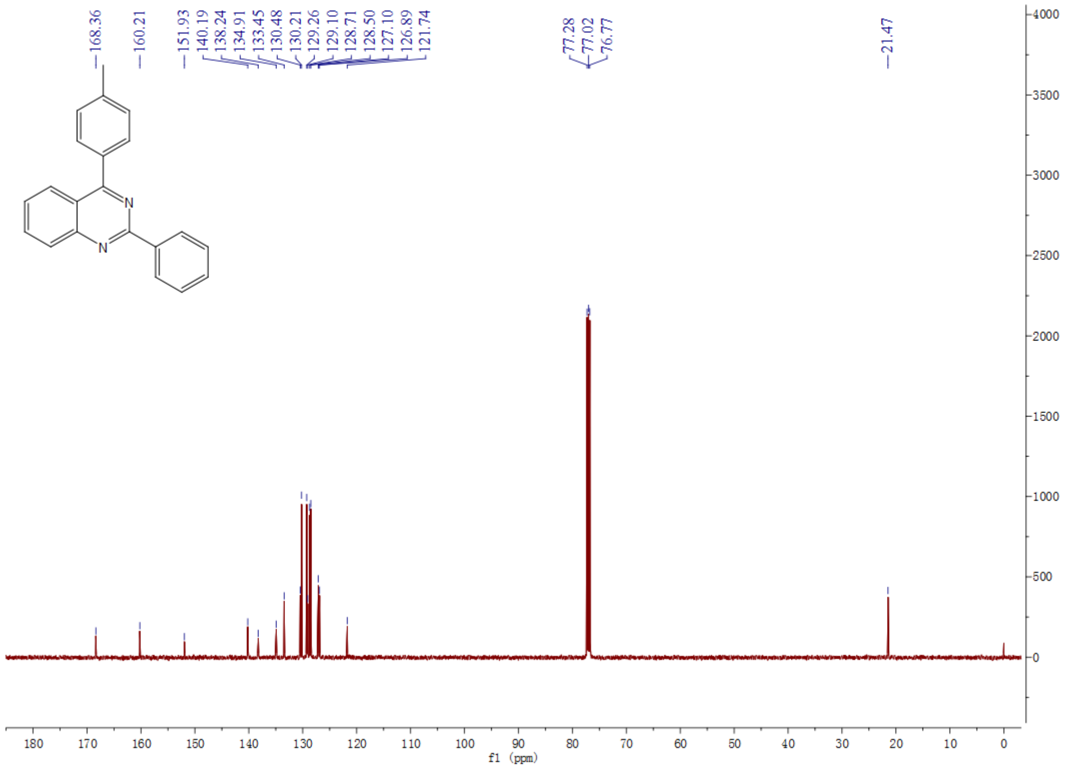

Figure S2. ^{1}H NMR of **3b** (500 MHz, $CDCl_3$) and ^{13}C NMR of **3b** (125 MHz, $CDCl_3$).

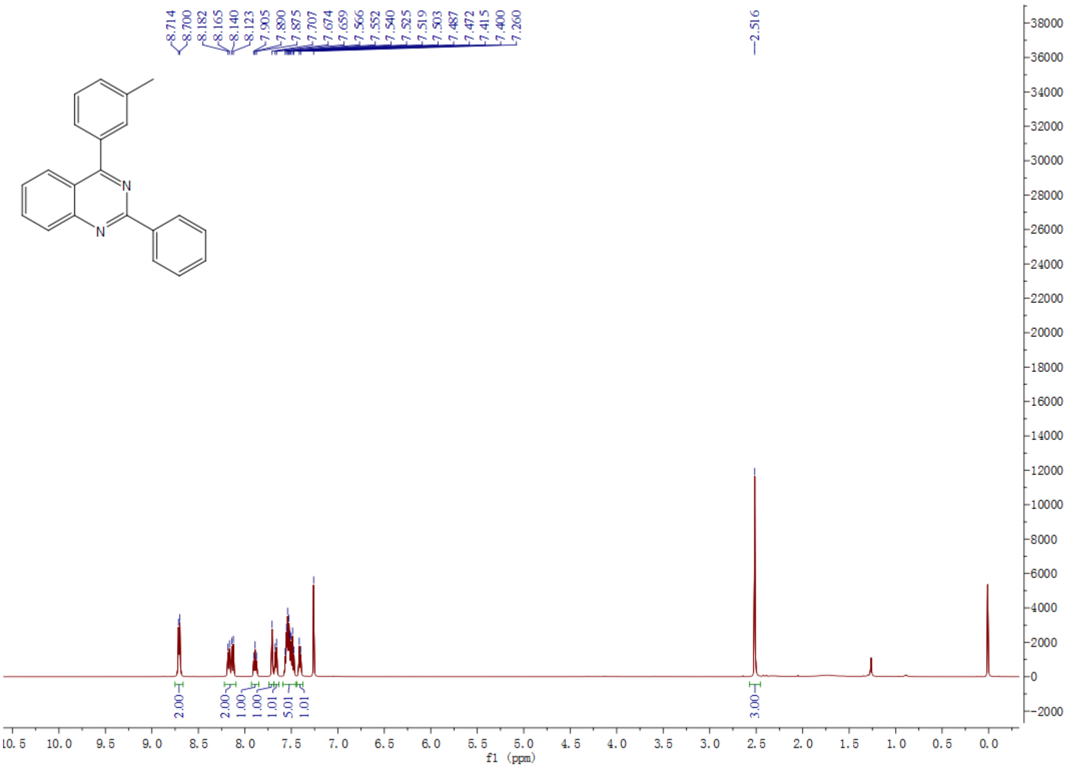

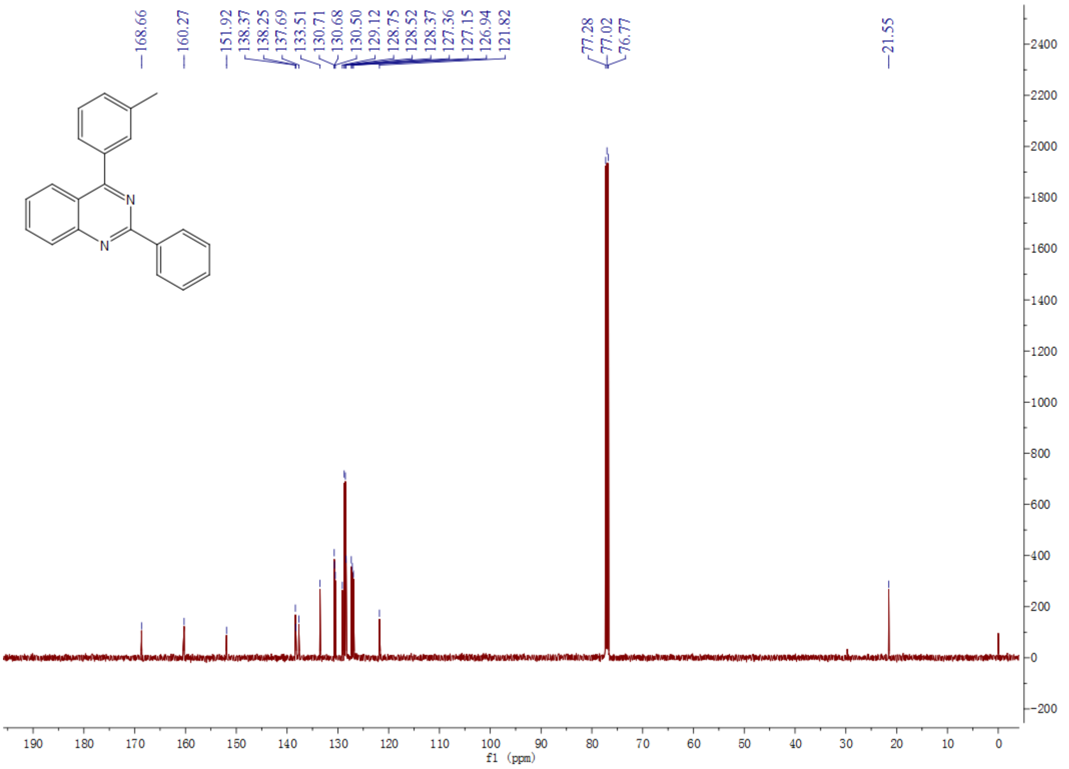

Figure S3. ^{1}H NMR of **3c** (500 MHz, $CDCl_3$) and ^{13}C NMR of **3c** (125 MHz, $CDCl_3$).

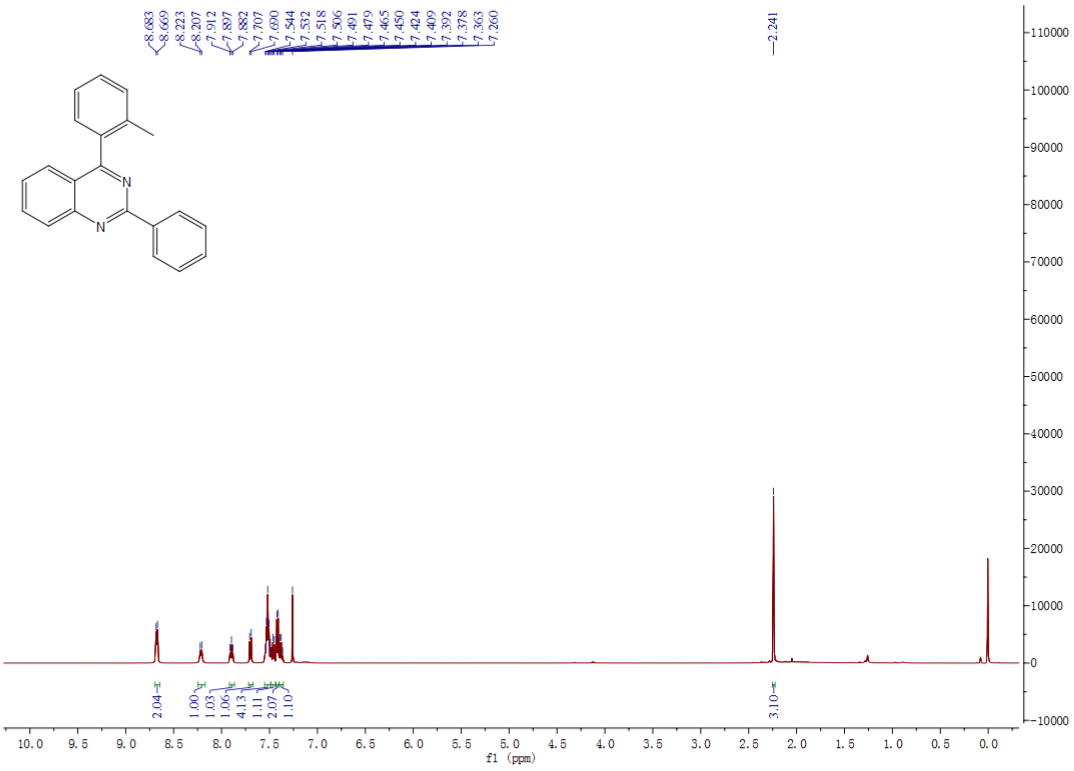

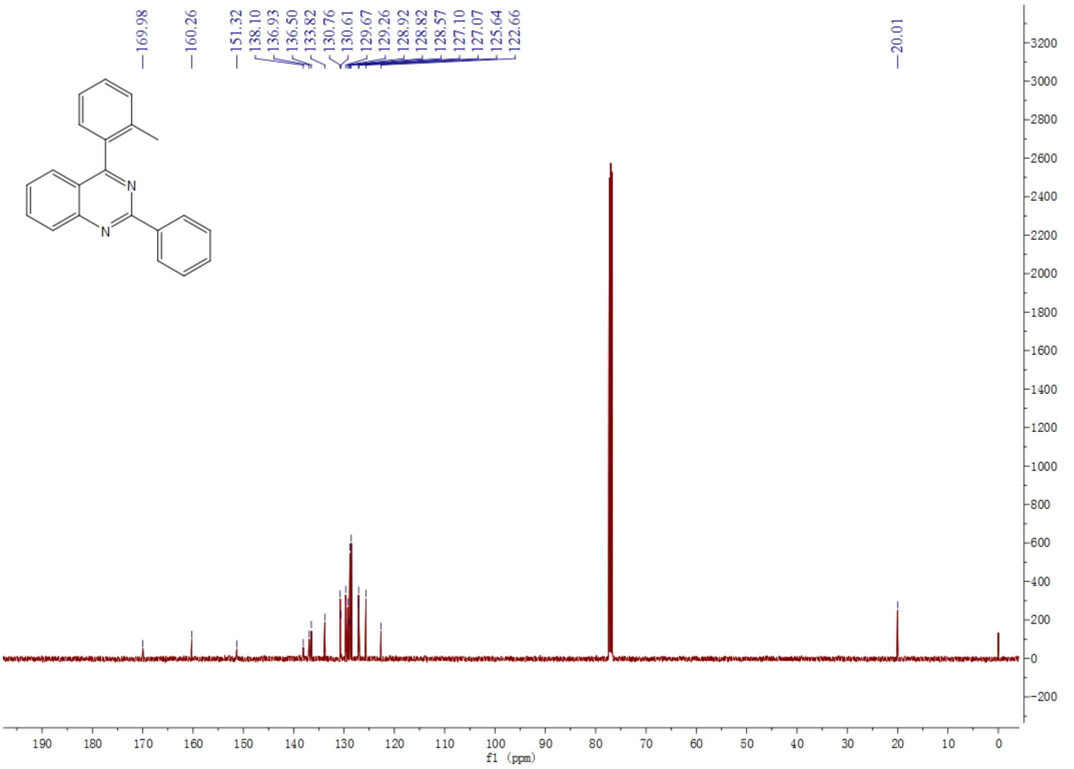

Figure S4. ^{1}H NMR of **3d** (500 MHz, $CDCl_3$) and ^{13}C NMR of **3d** (125 MHz, $CDCl_3$).

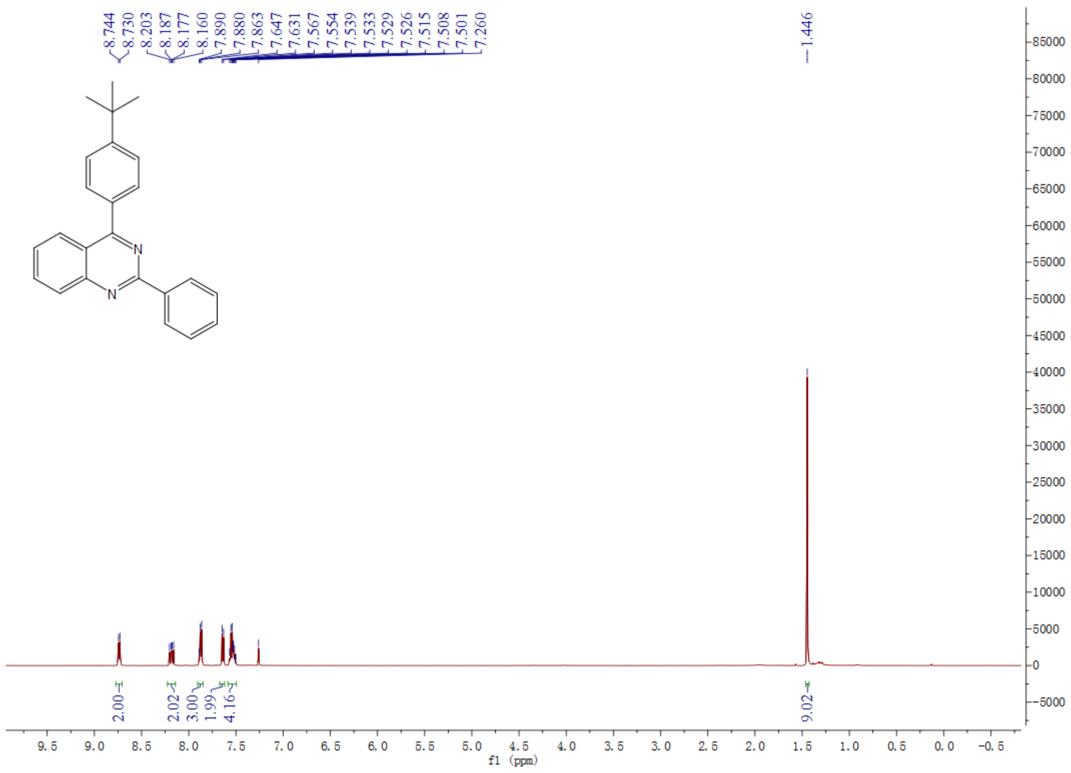

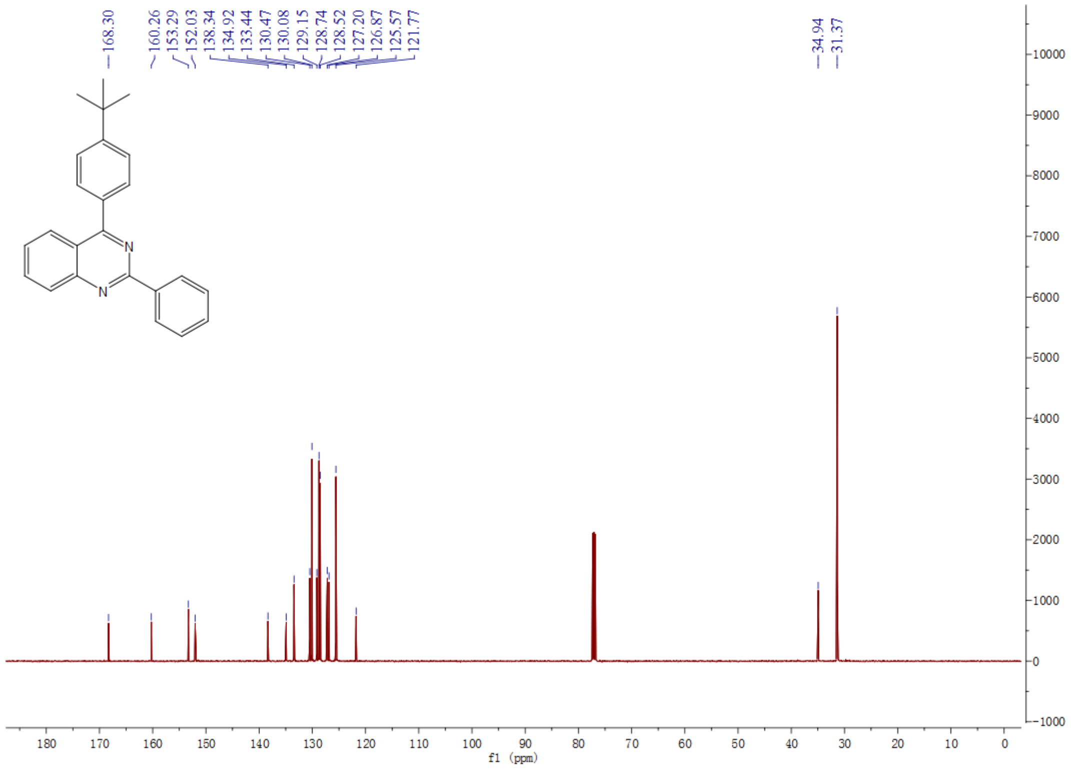

Figure S5. ^{1}H NMR of **3e** (500 MHz, $CDCl_3$) and ^{13}C NMR of **3e** (125 MHz, $CDCl_3$).

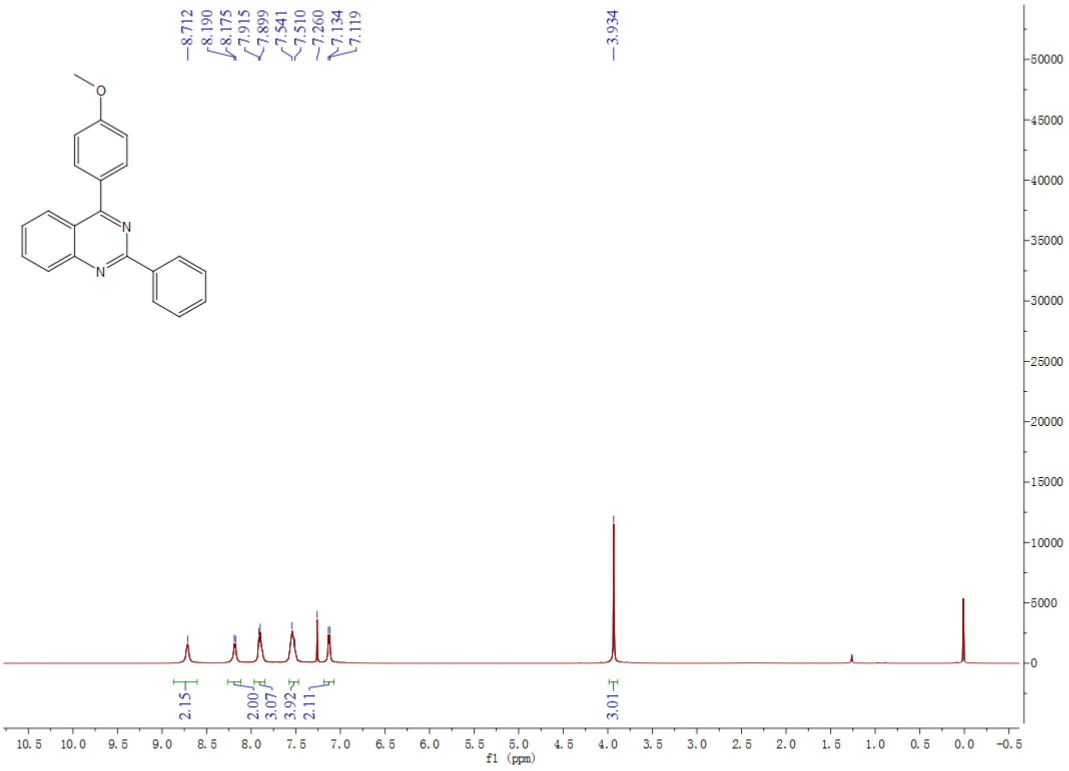

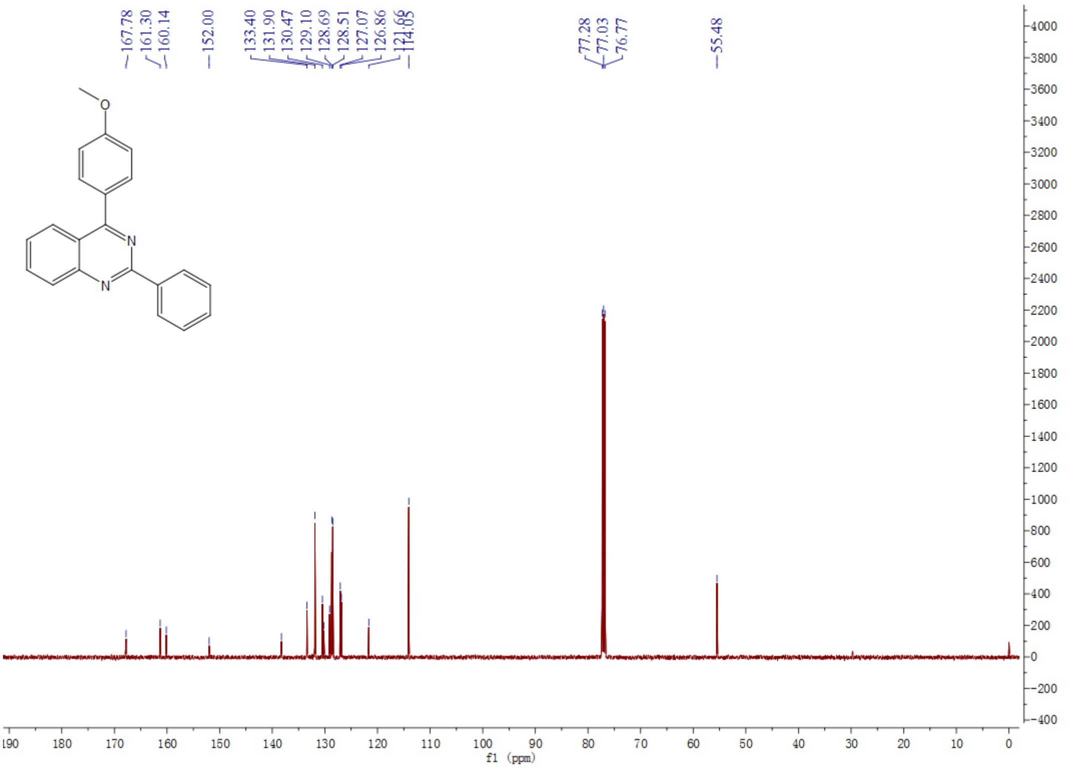

Figure S6. ^{1}H NMR of **3f** (500 MHz, $CDCl_3$) and ^{13}C NMR of **3f** (125 MHz, $CDCl_3$).

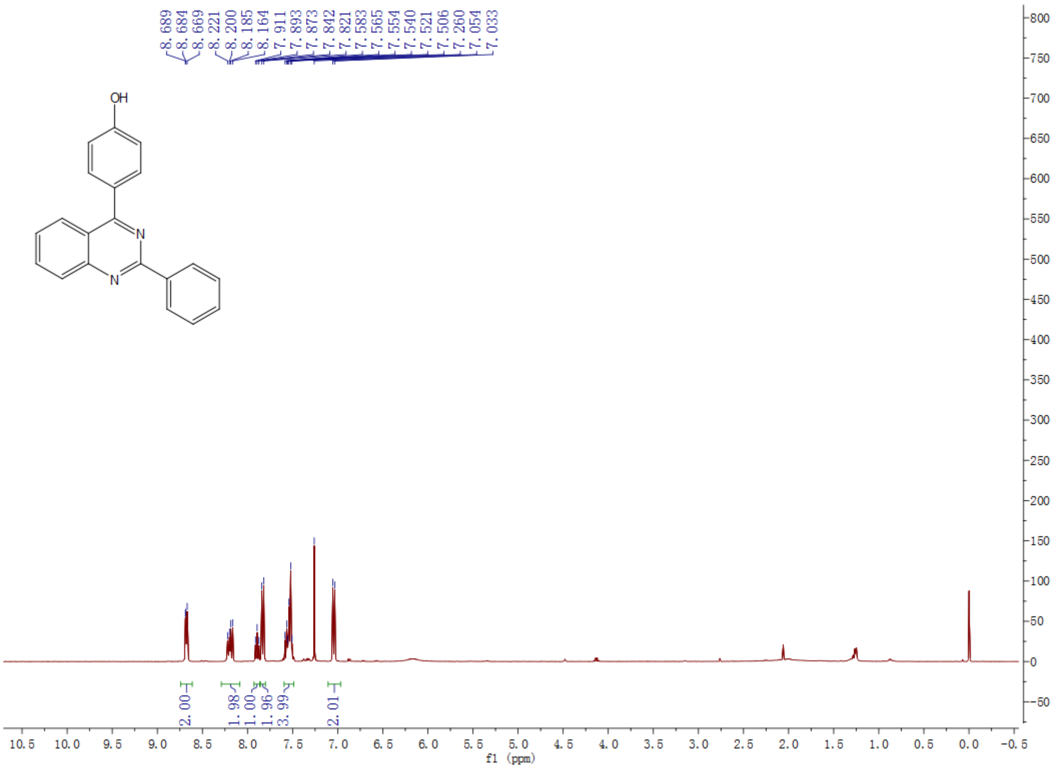

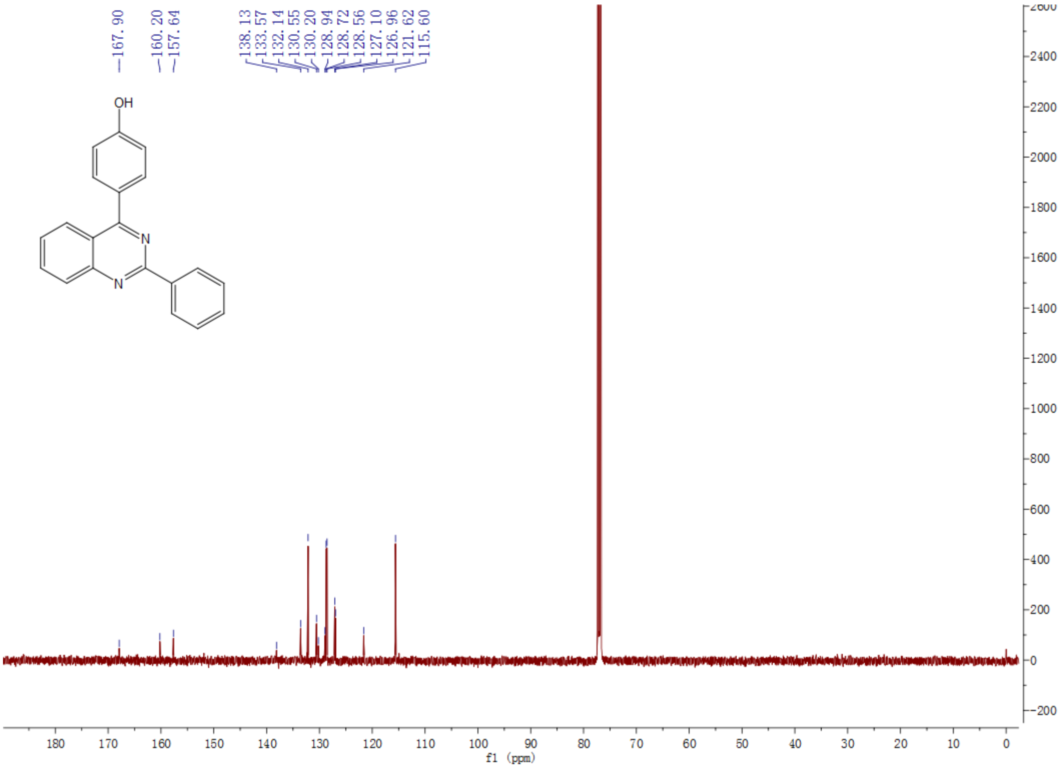

Figure S7. ^{1}H NMR of **3g** (500 MHz, $CDCl_3$) and ^{13}C NMR of **3g** (125 MHz, $CDCl_3$).

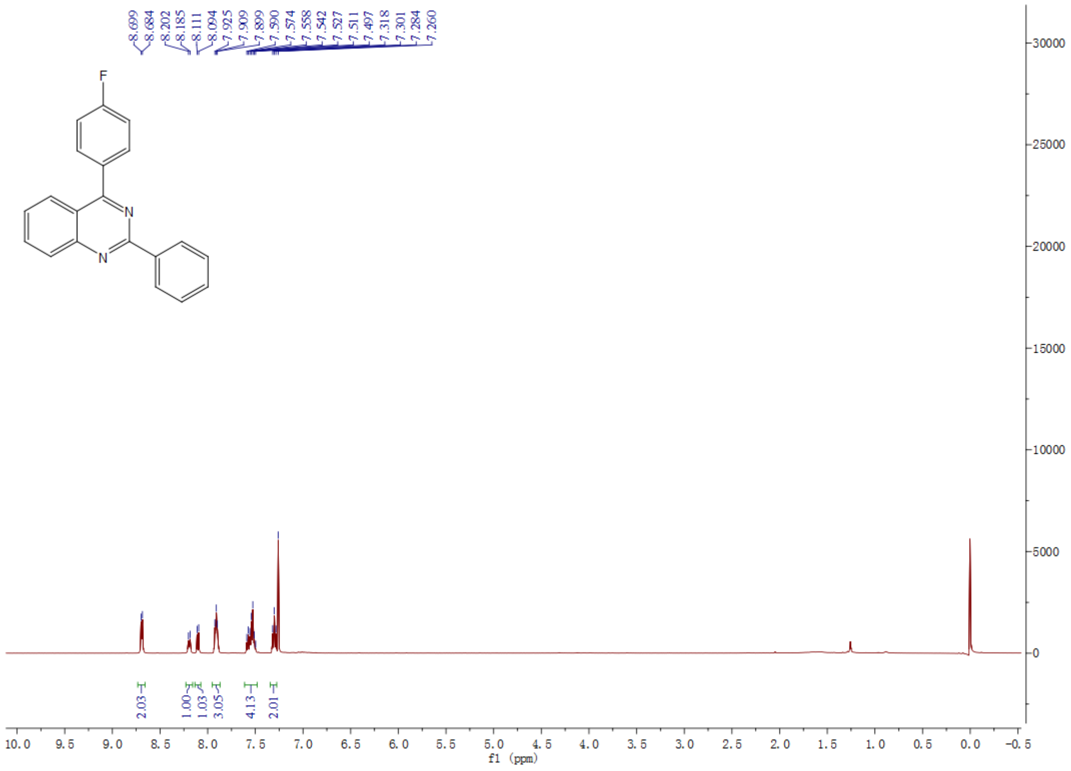

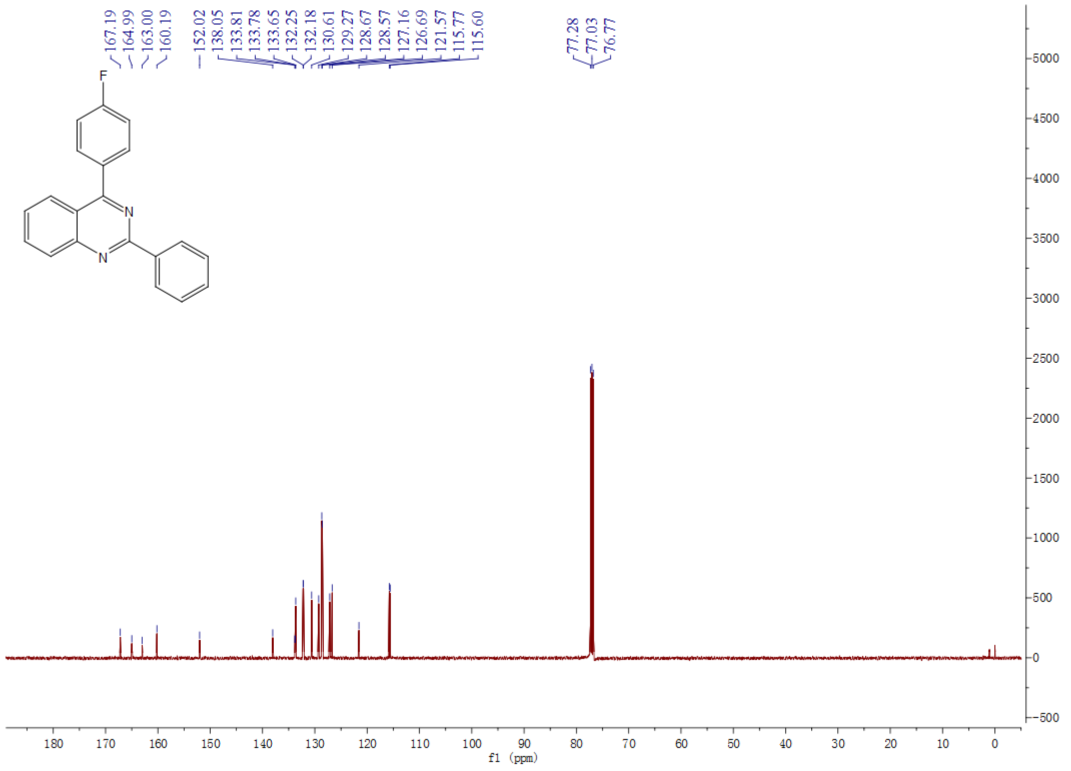

Figure S8. ^{1}H NMR of **3h** (500 MHz, $CDCl_3$) and ^{13}C NMR of **3h** (125 MHz, $CDCl_3$).

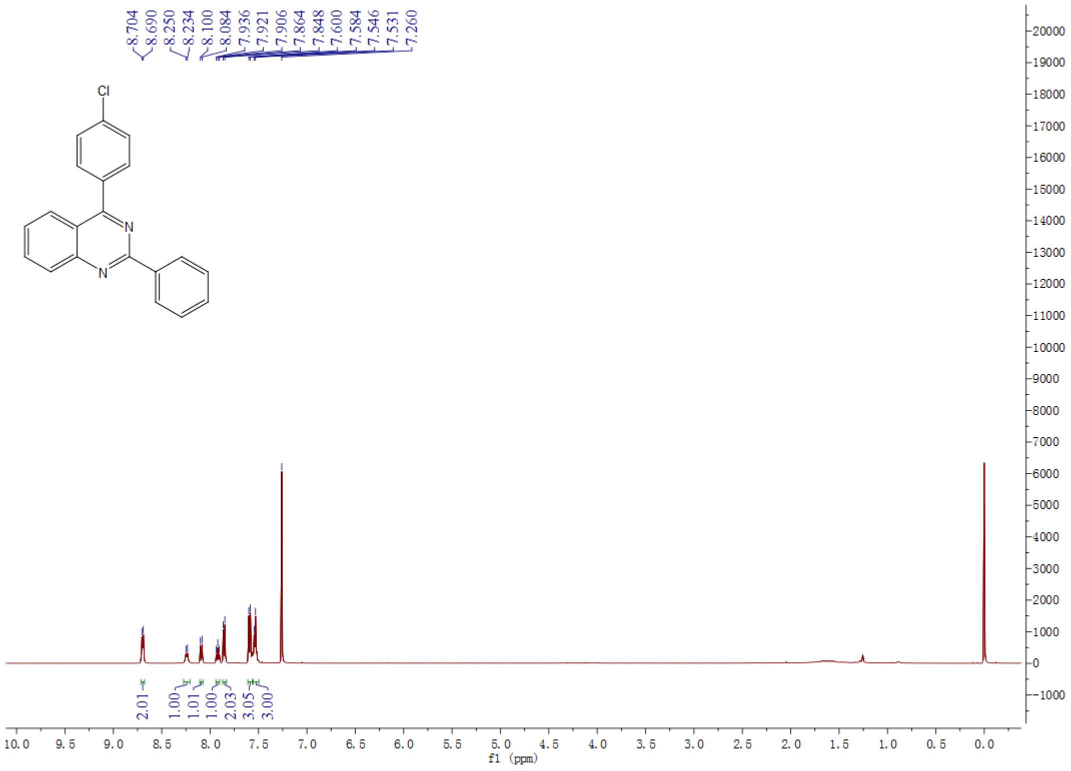

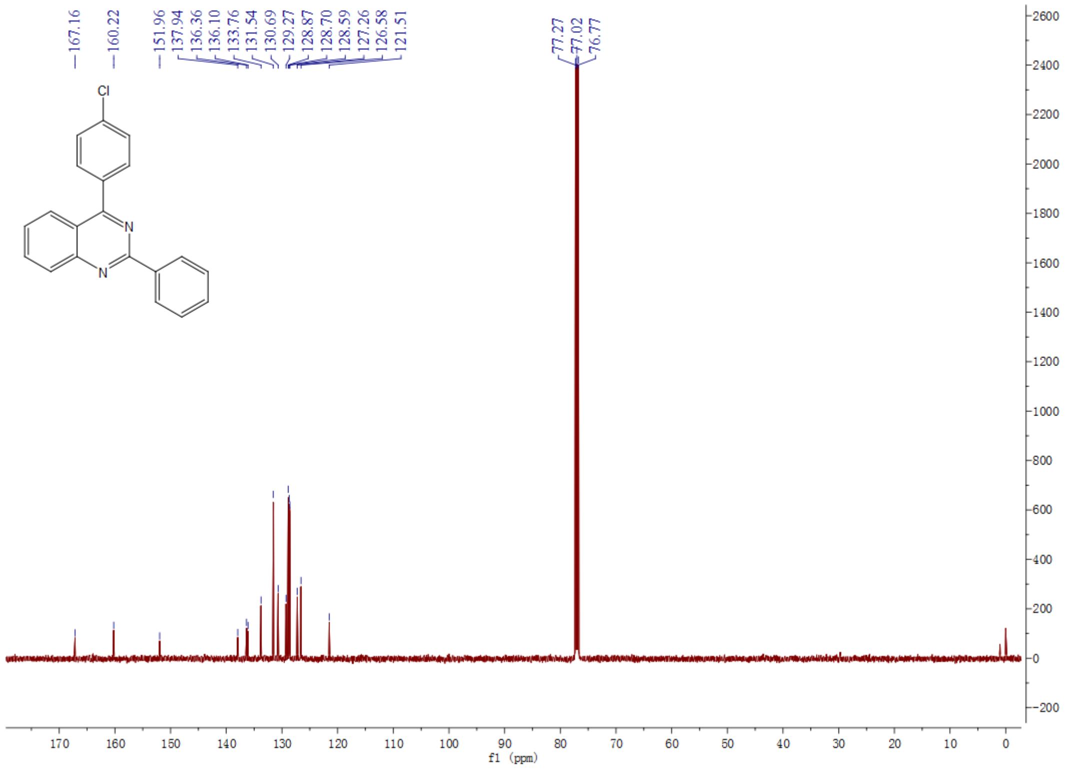

Figure S9. ^{1}H NMR of **3i** (500 MHz, $CDCl_3$) and ^{13}C NMR of **3i** (125 MHz, $CDCl_3$).

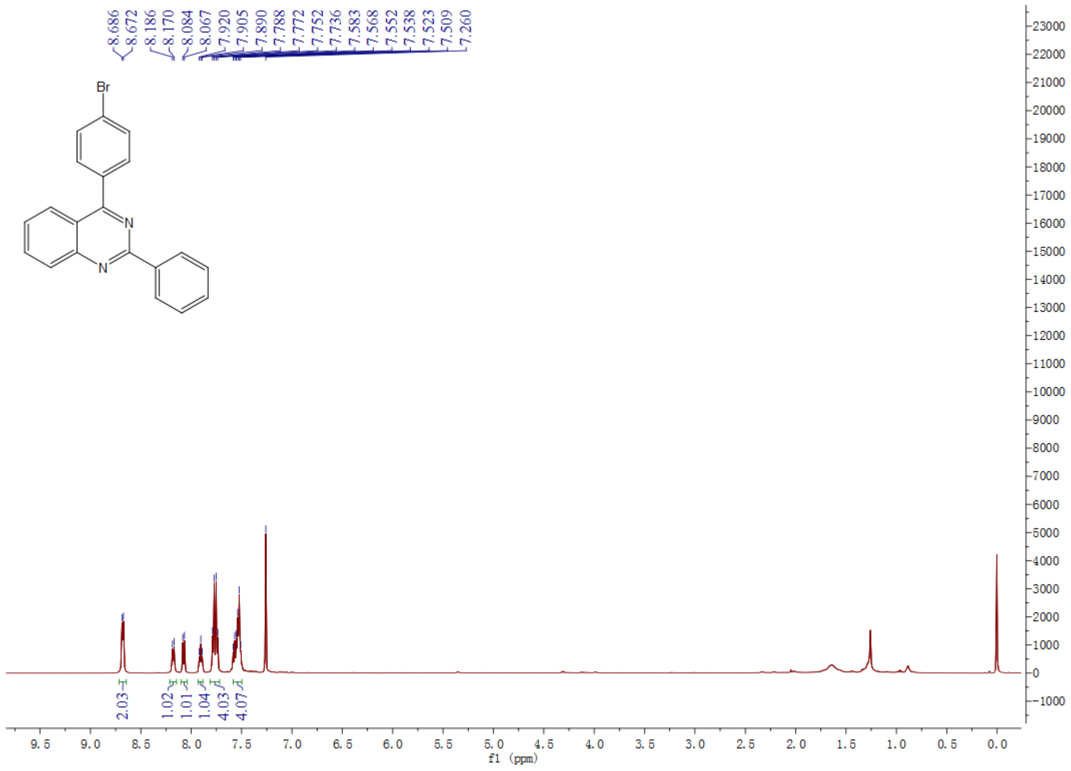

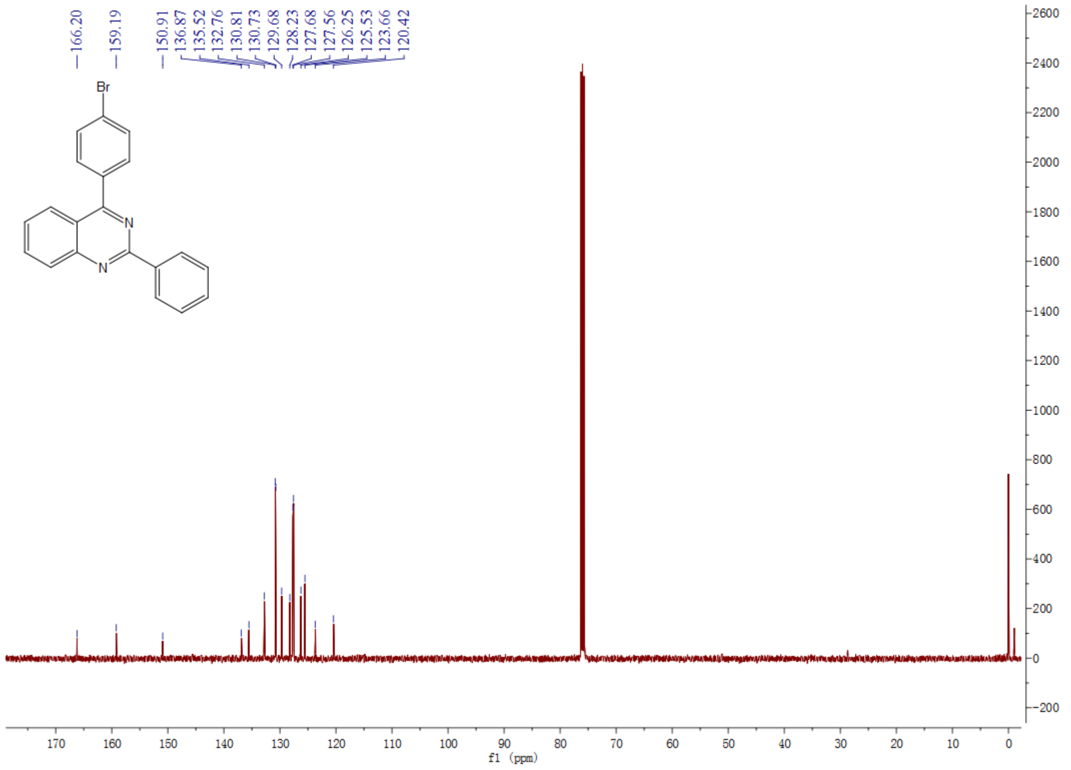

Figure S10. ^{1}H NMR of **3j** (500 MHz, $CDCl_3$) and ^{13}C NMR of **3j** (125 MHz, $CDCl_3$).

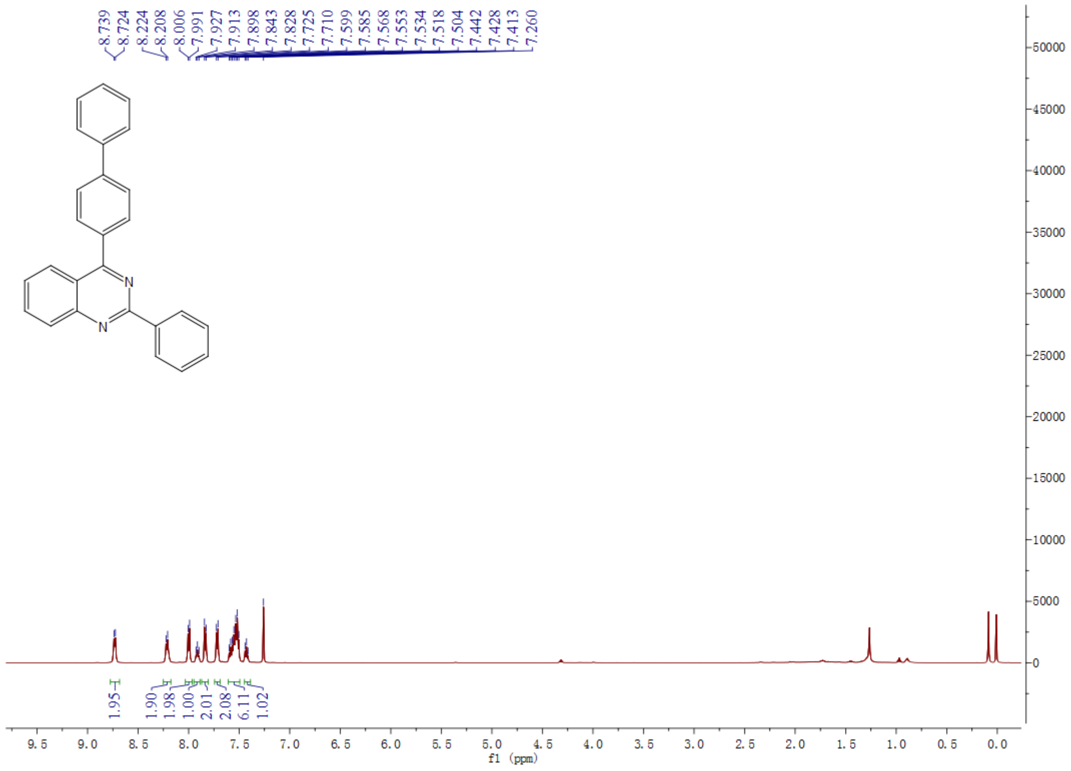

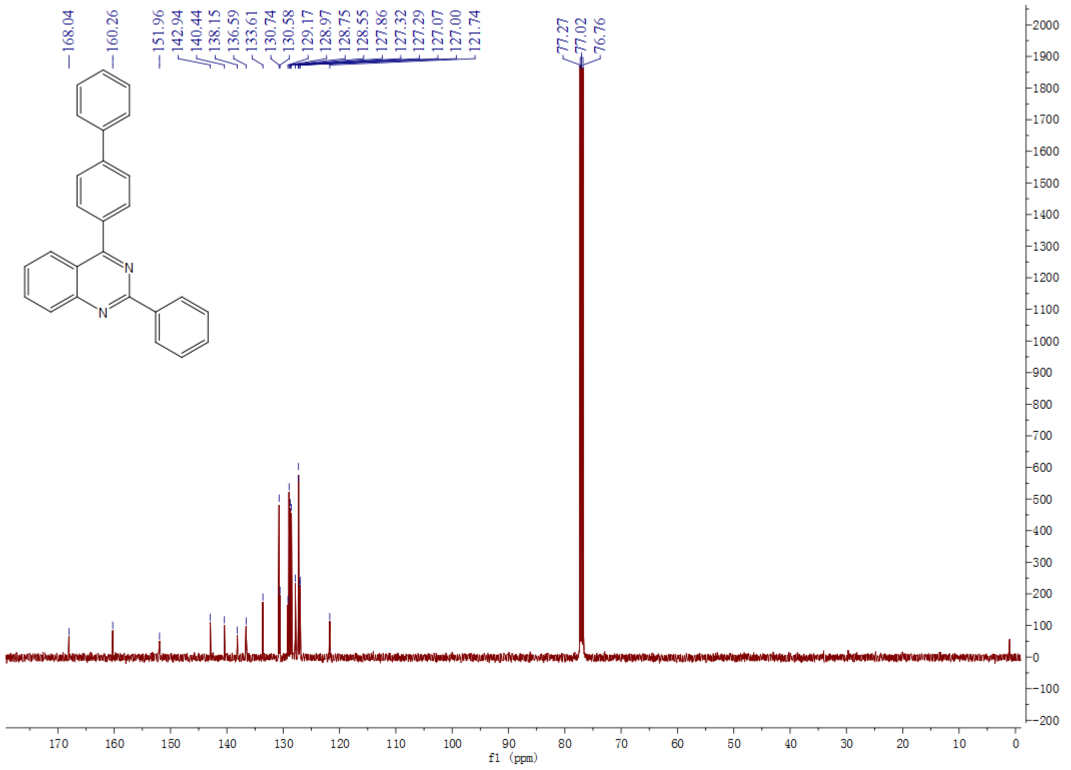

Figure S11. ^{1}H NMR of **3l** (500 MHz, $CDCl_3$) and ^{13}C NMR of **3l** (125 MHz, $CDCl_3$).

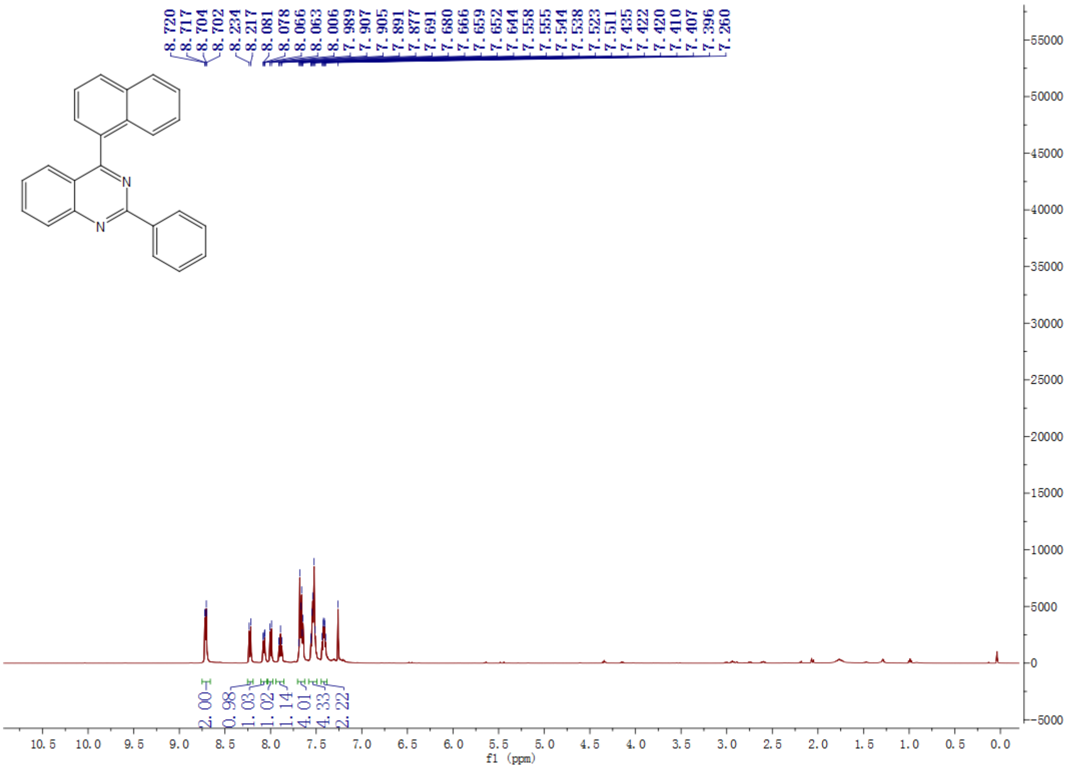

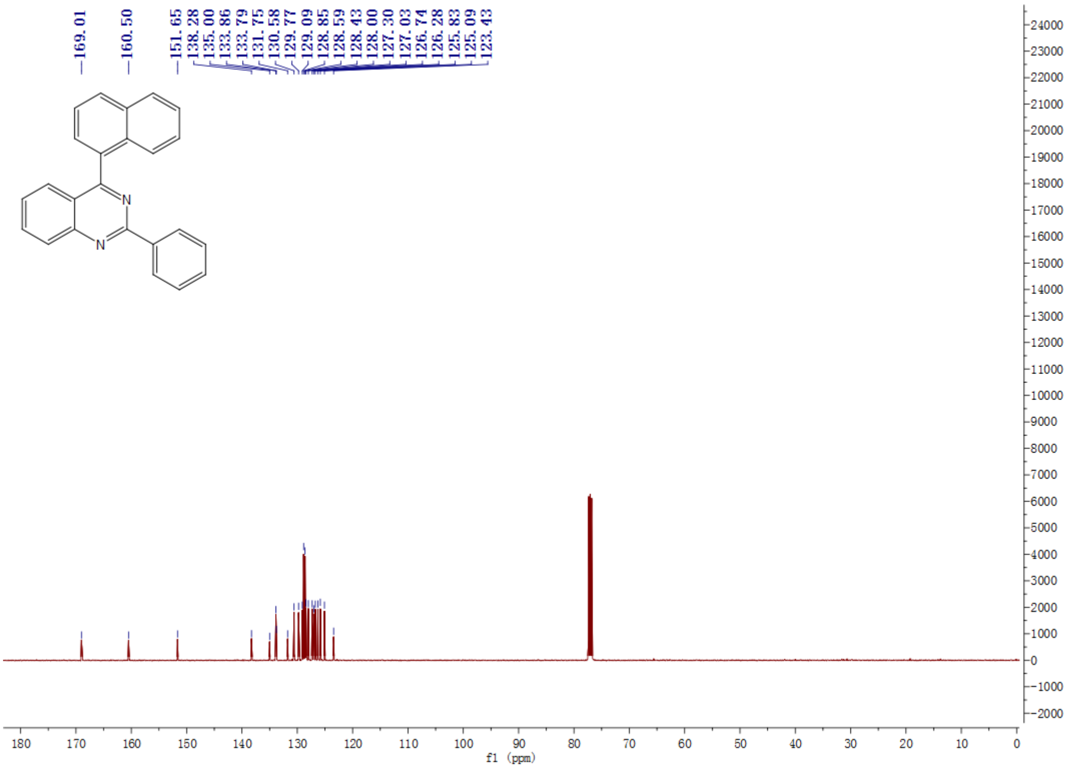

Figure S12. ^{1}H NMR of **3m** (500 MHz, $CDCl_3$) and ^{13}C NMR of **3m** (125 MHz, $CDCl_3$).

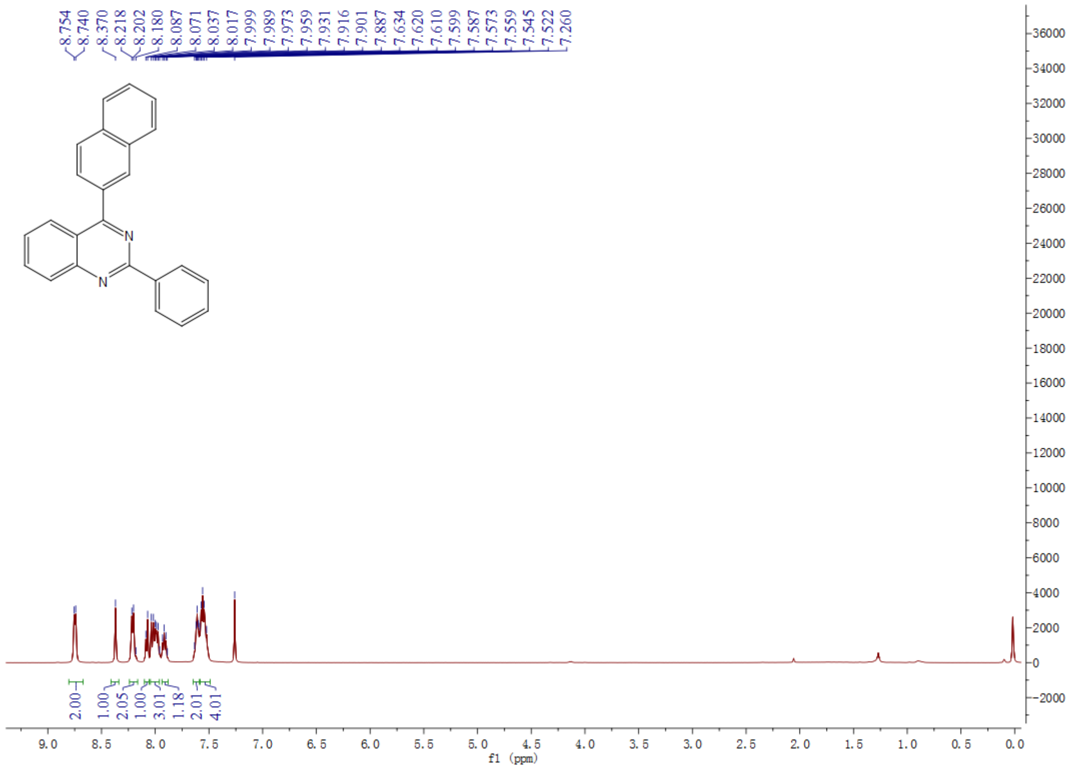

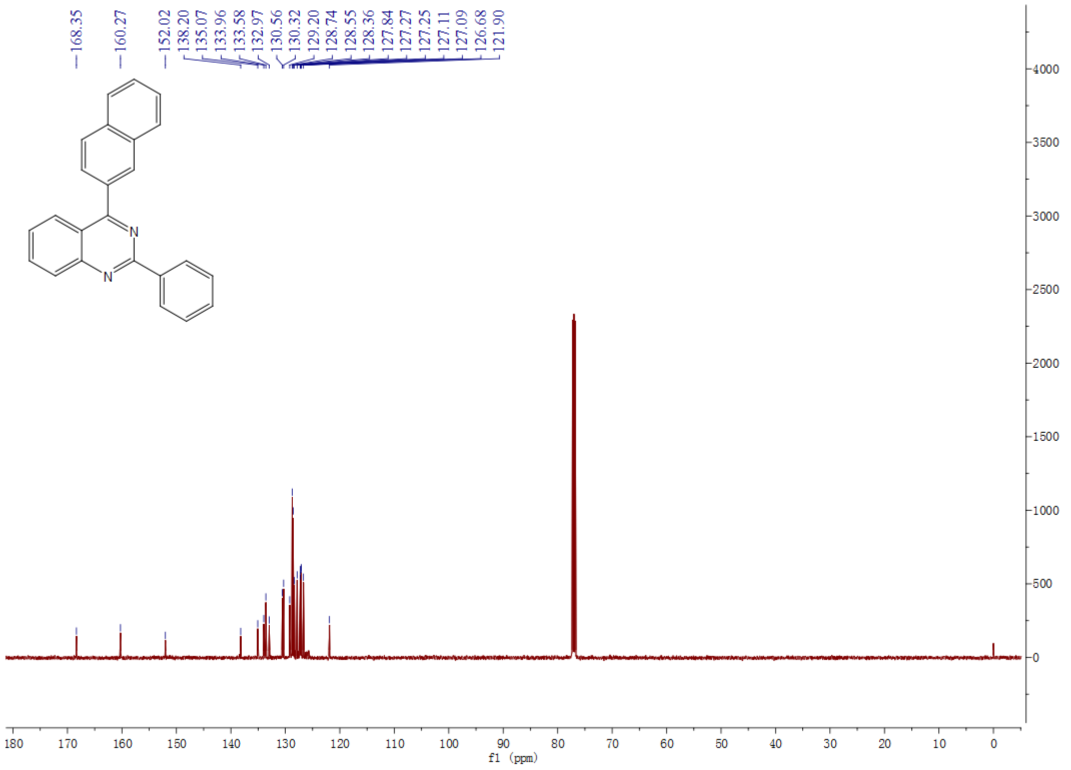

Figure S13. ^{1}H NMR of **3n** (500 MHz, $CDCl_3$) and ^{13}C NMR of **3n** (125 MHz, $CDCl_3$).

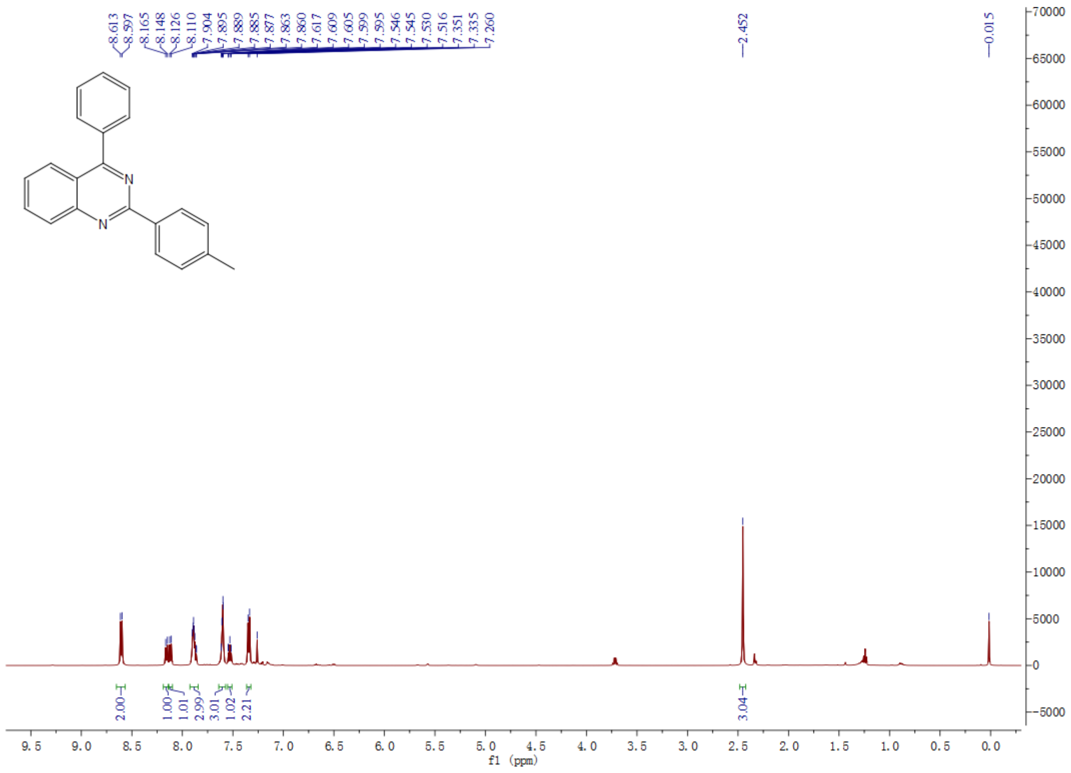

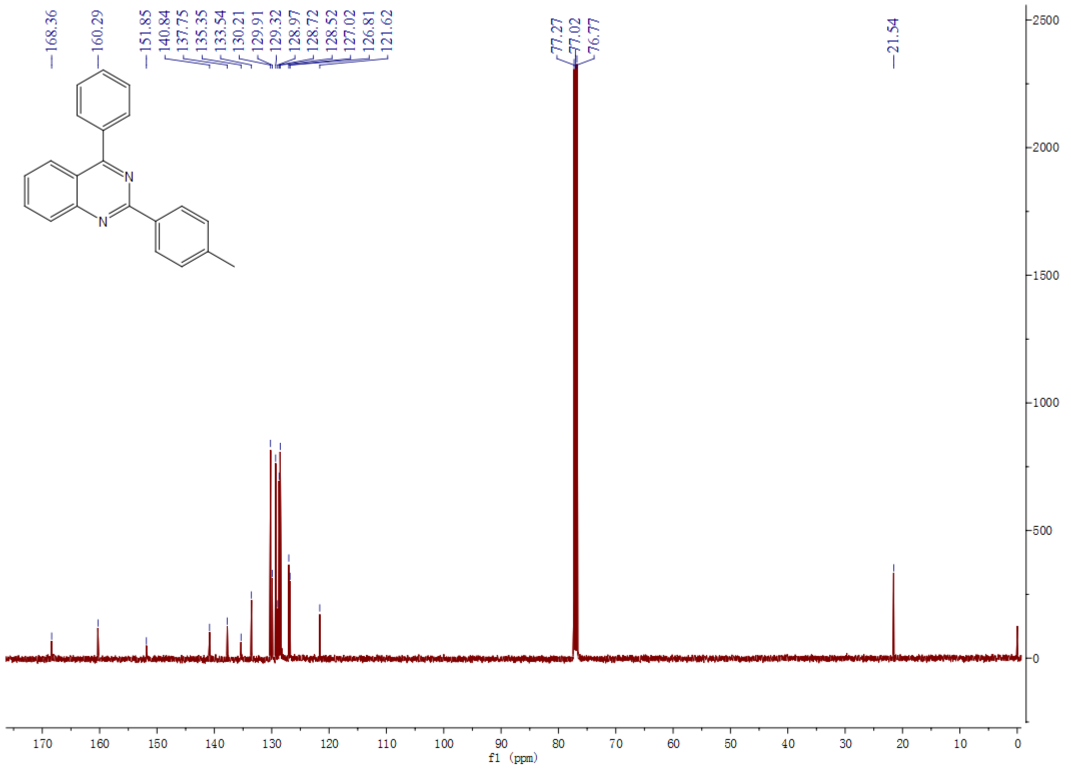

Figure S14. ^{1}H NMR of **3o** (500 MHz, $CDCl_3$) and ^{13}C NMR of **3o** (125 MHz, $CDCl_3$).

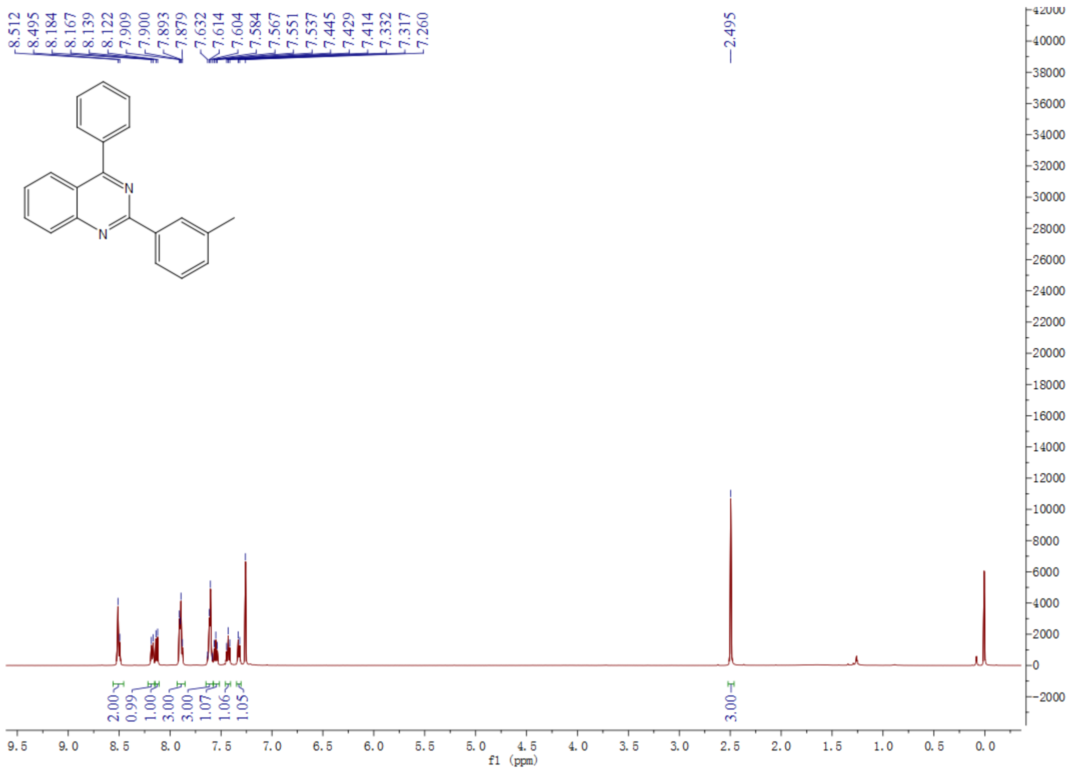

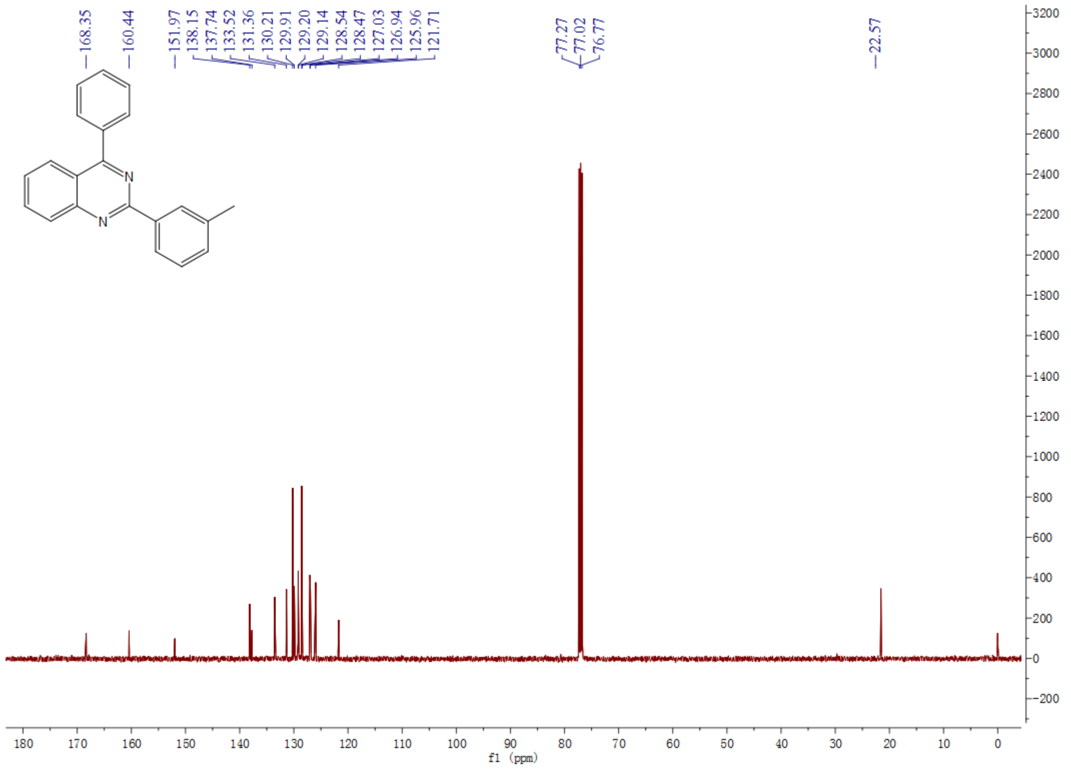

Figure S15. ^{1}H NMR of **3p** (500 MHz, $CDCl_3$) and ^{13}C NMR of **3p** (125 MHz, $CDCl_3$).

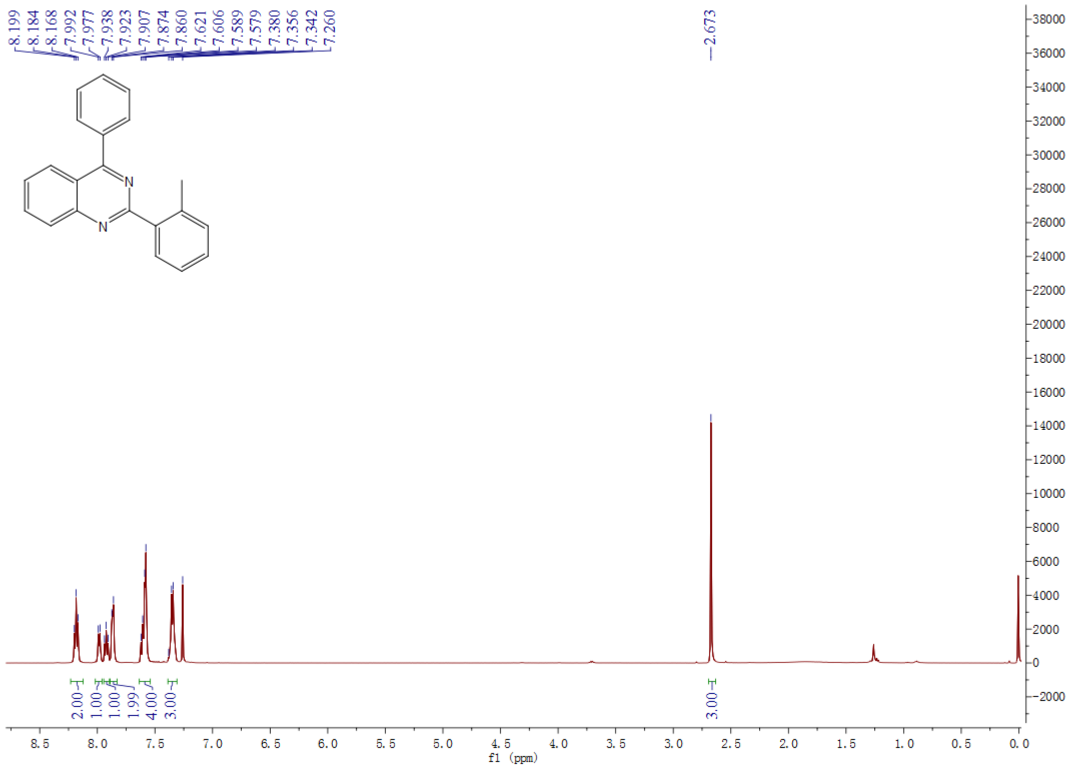

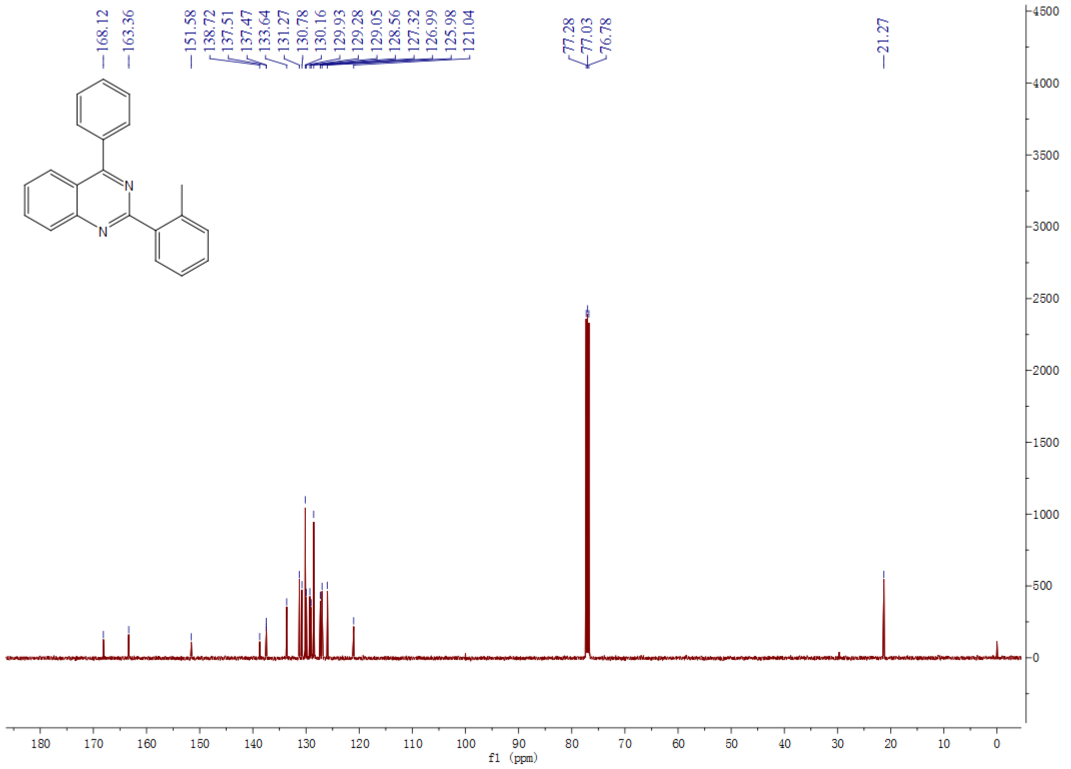

Figure S16. ^{1}H NMR of **3q** (500 MHz, $CDCl_3$) and ^{13}C NMR of **3q** (125 MHz, $CDCl_3$).

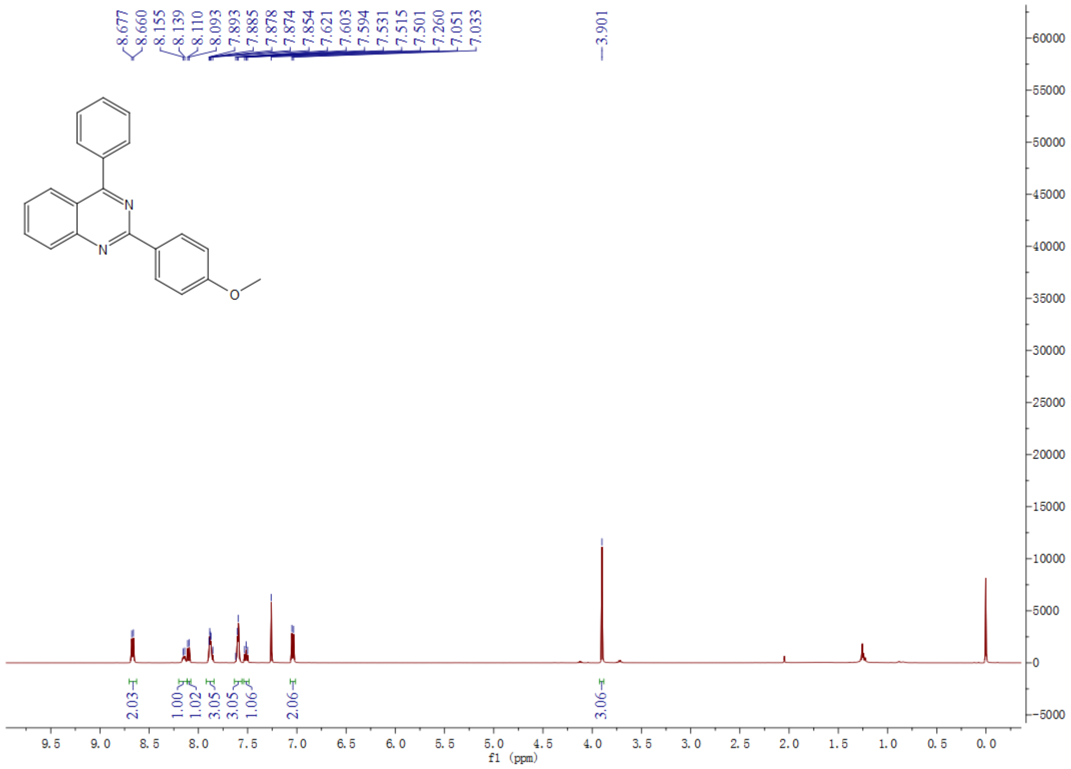

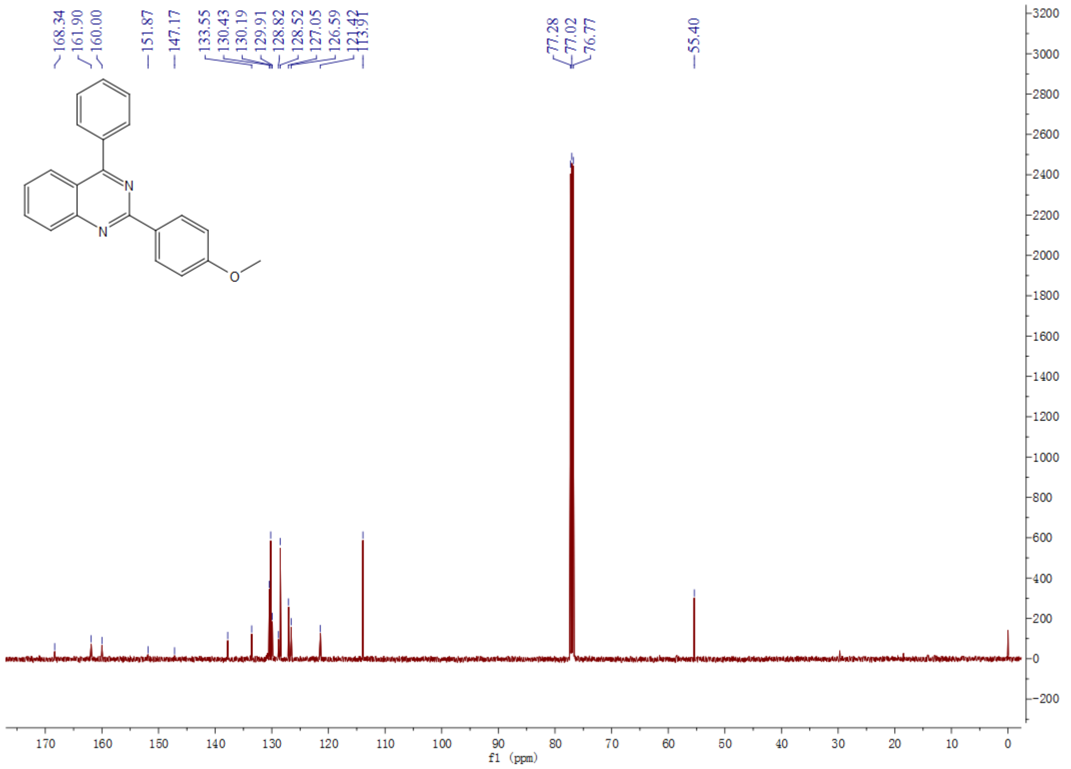

Figure S17. ^{1}H NMR of **3r** (500 MHz, $CDCl_3$) and ^{13}C NMR of **3r** (125 MHz, $CDCl_3$).

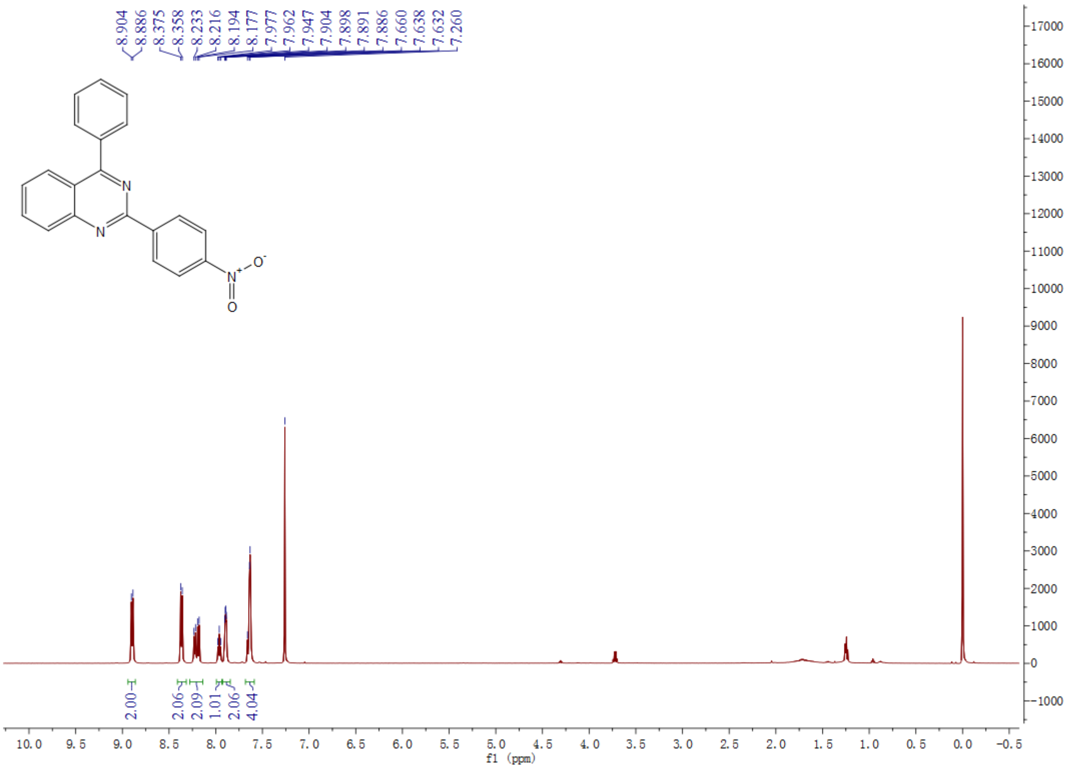

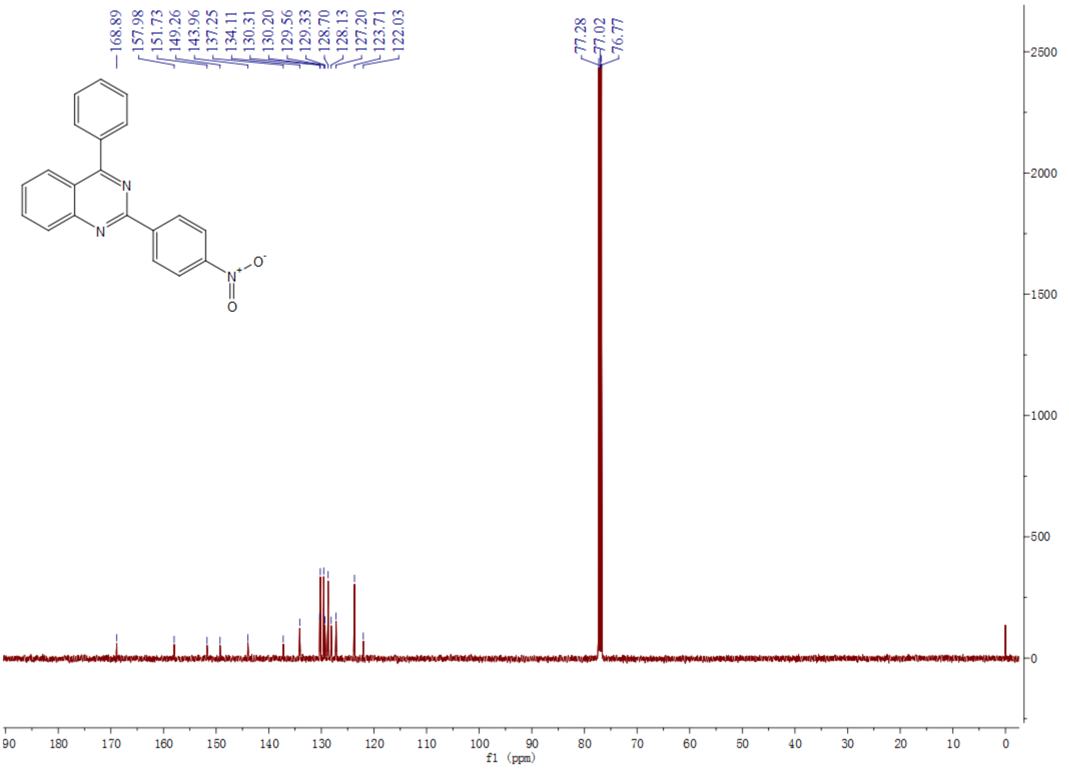

Figure S18. ^{1}H NMR of **3s** (500 MHz, $CDCl_3$) and ^{13}C NMR of **3s** (125 MHz, $CDCl_3$).

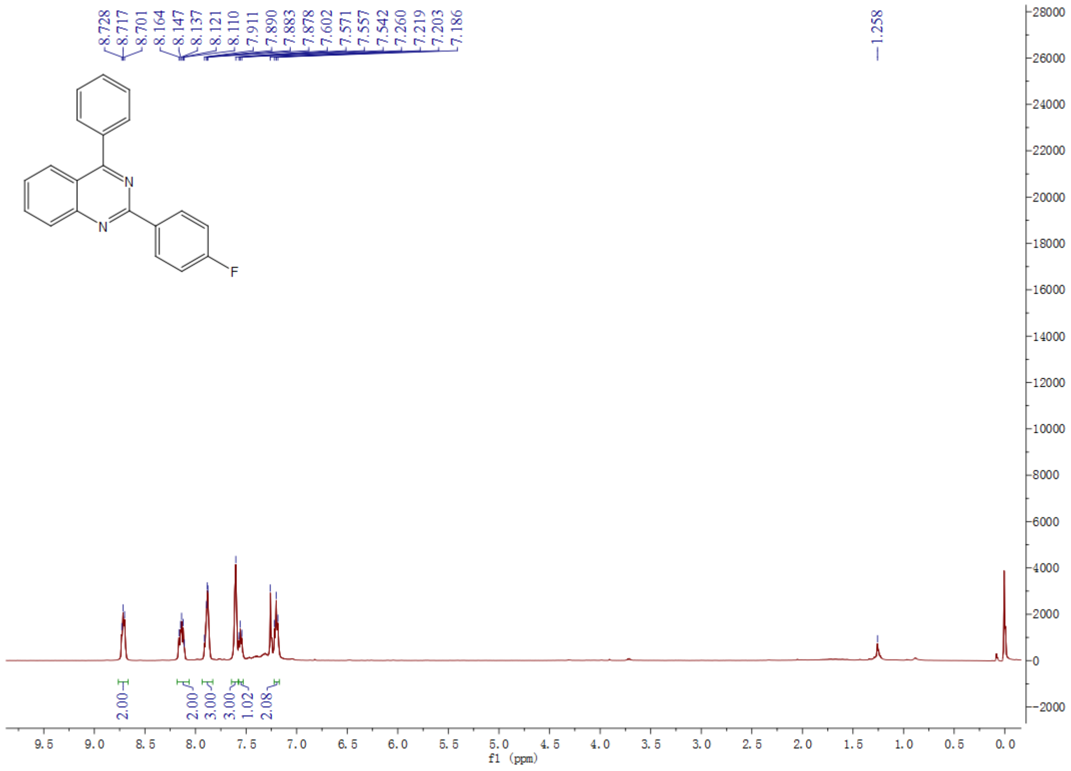

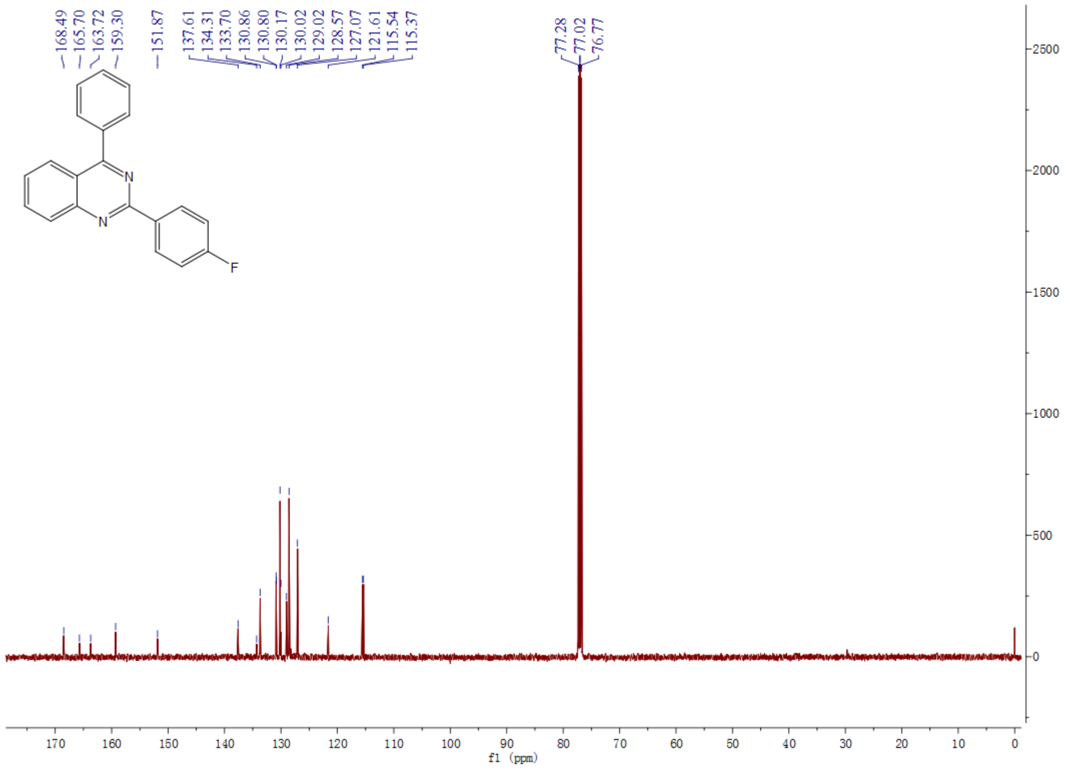

Figure S19. ^{1}H NMR of **3t** (500 MHz, $CDCl_3$) and ^{13}C NMR of **3t** (125 MHz, $CDCl_3$).

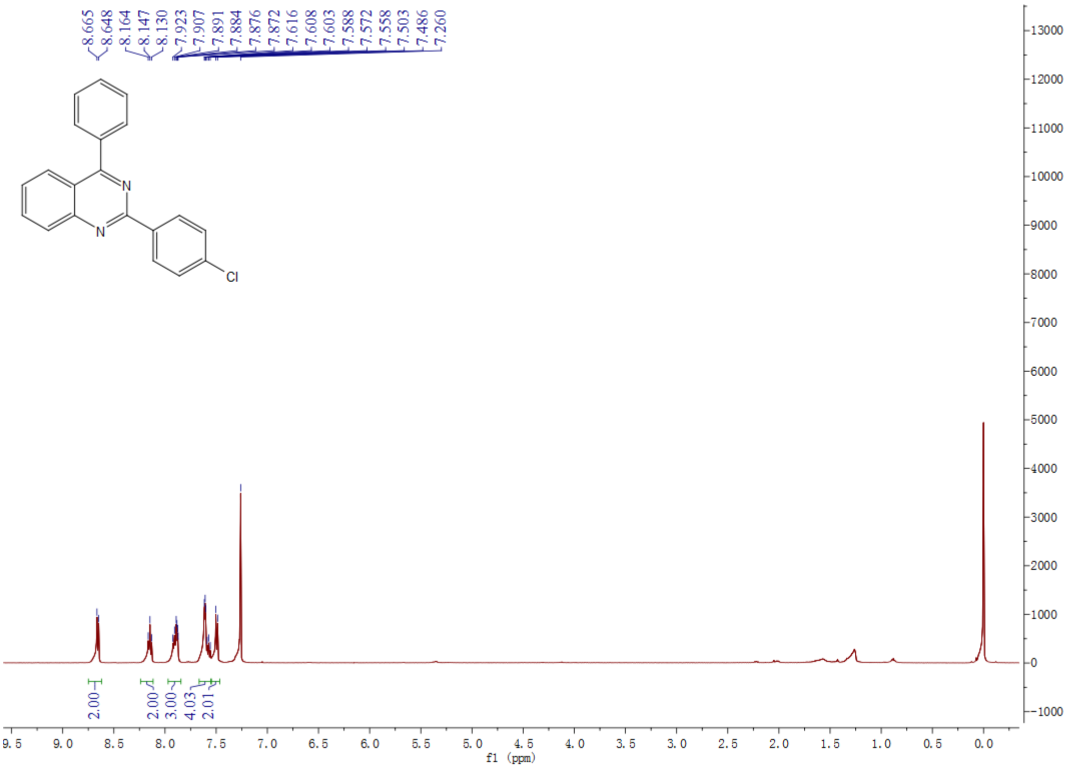

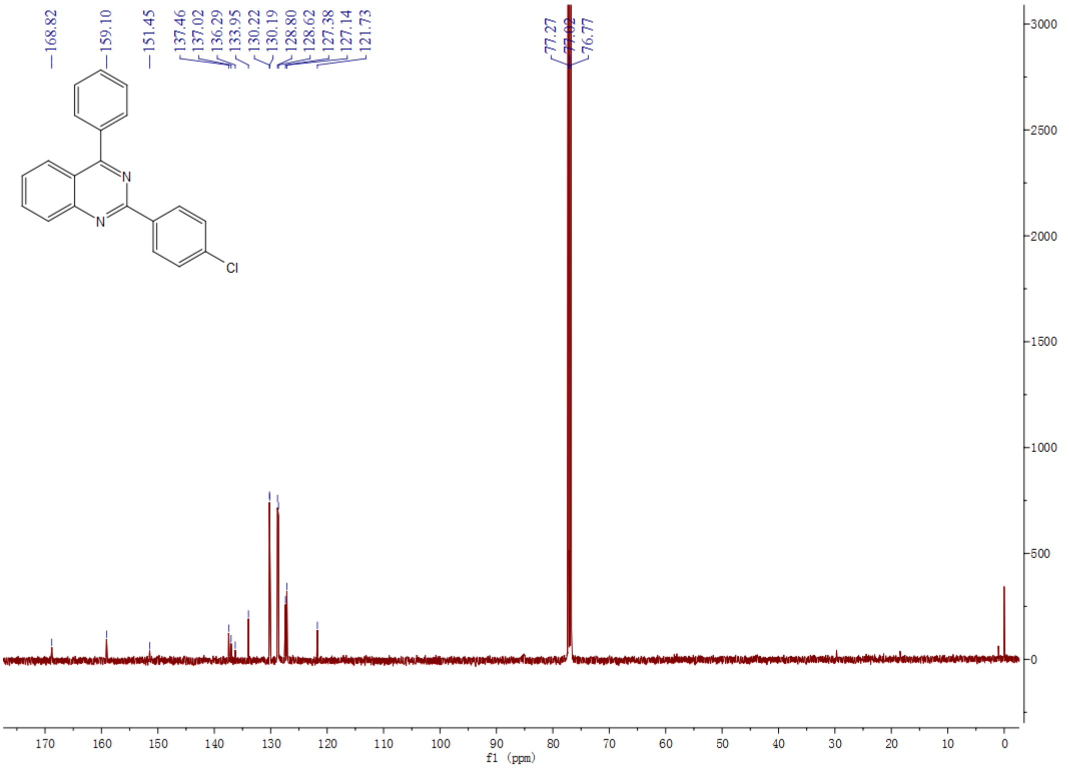

Figure S20. ^{1}H NMR of **3u** (500 MHz, $CDCl_3$) and ^{13}C NMR of **3u** (125 MHz, $CDCl_3$).

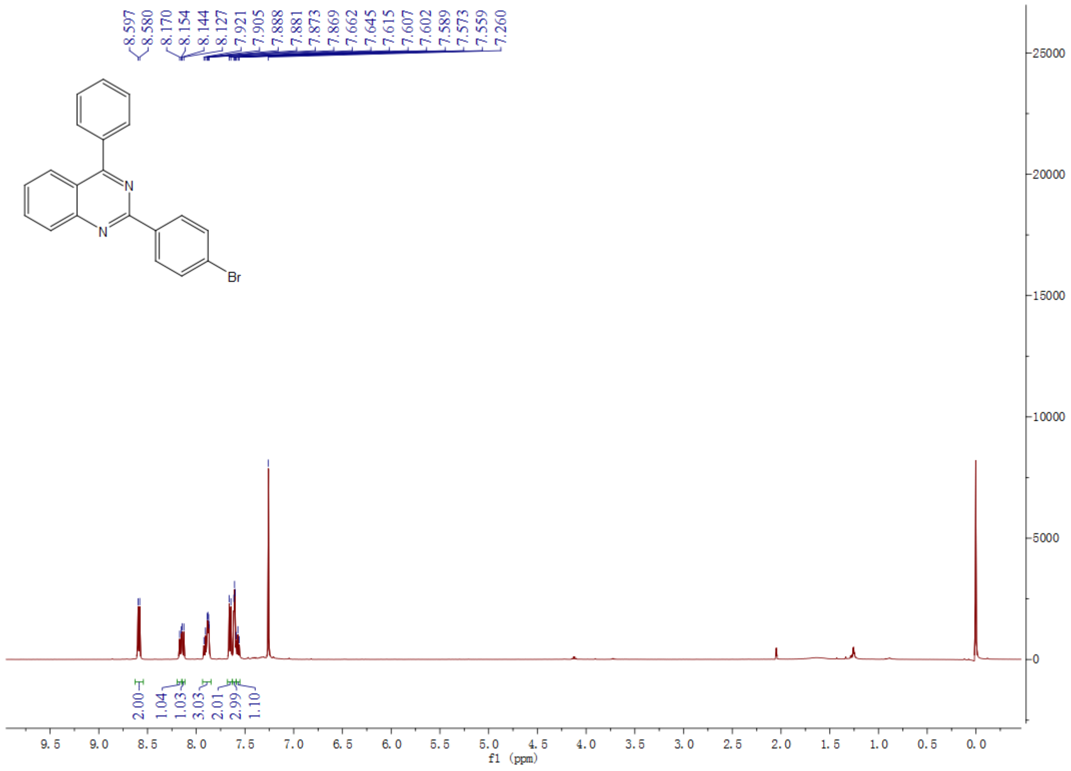

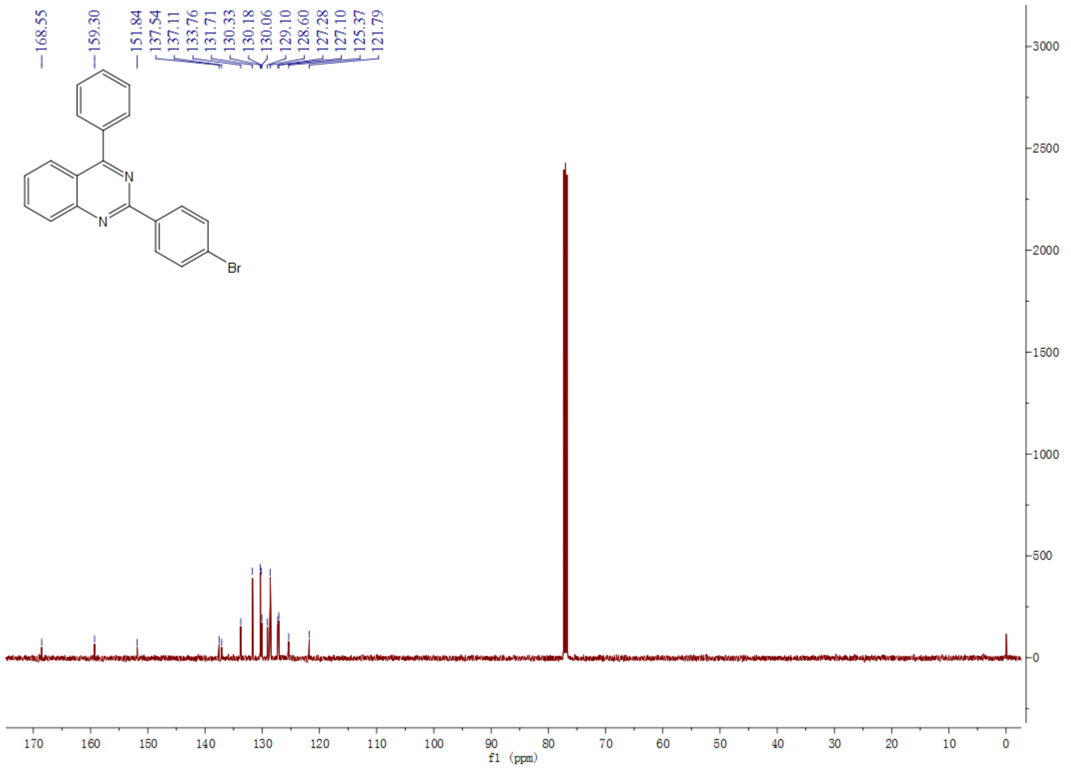

Figure S21. ^{1}H NMR of **3v** (500 MHz, $CDCl_3$) and ^{13}C NMR of **3v** (125 MHz, $CDCl_3$).

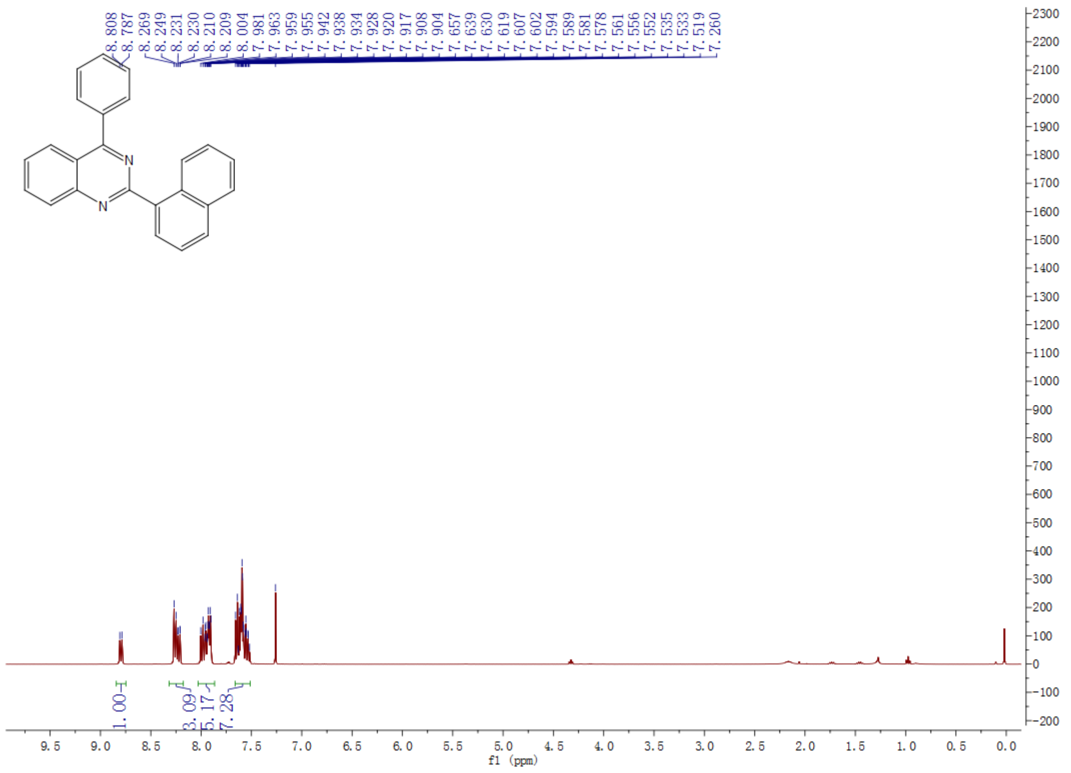

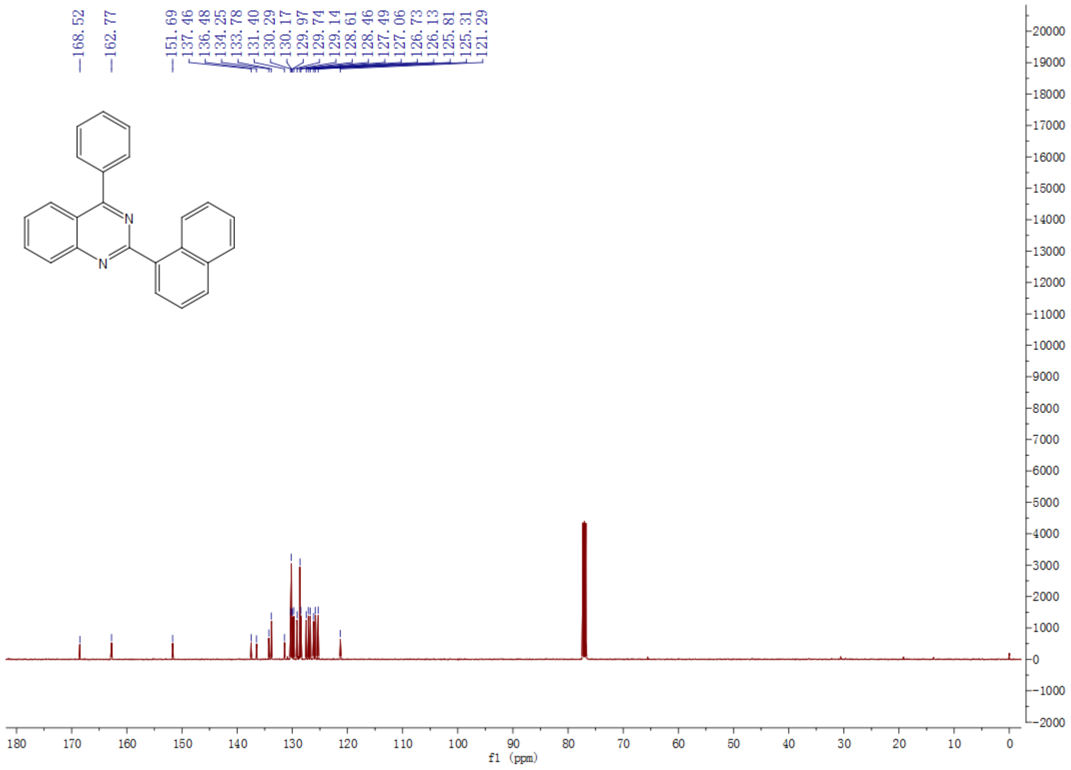

Figure S22. ^{1}H NMR of **3w** (500 MHz, $CDCl_3$) and ^{13}C NMR of **3w** (125 MHz, $CDCl_3$).

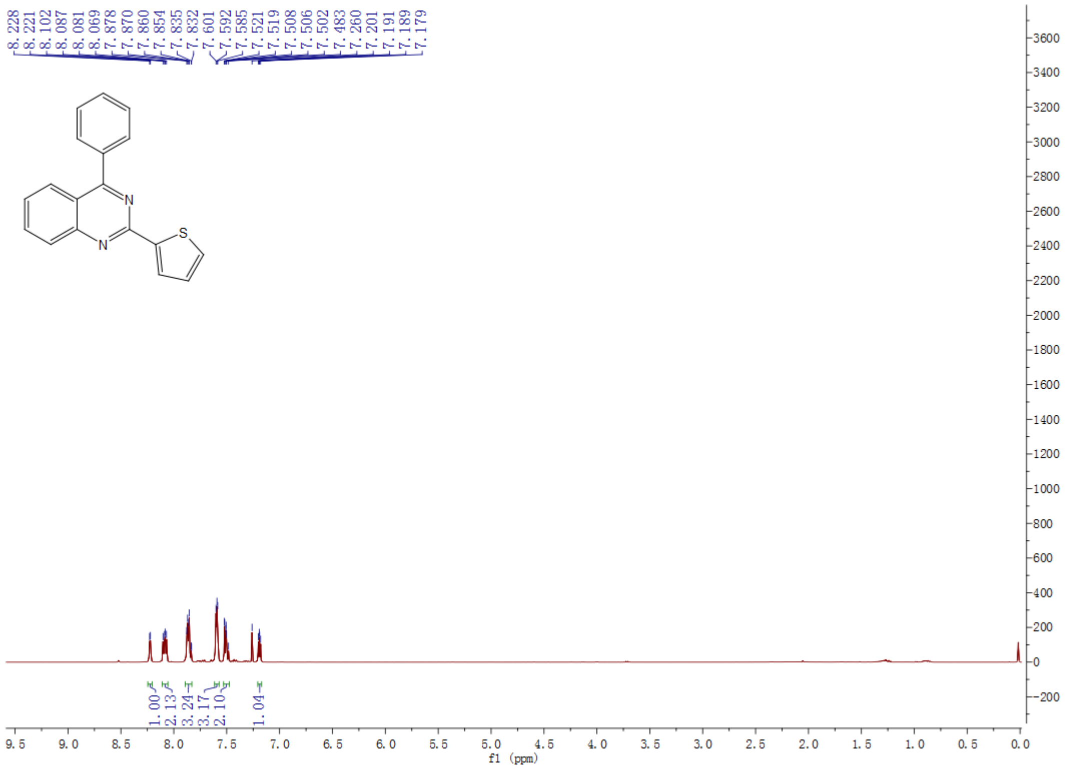

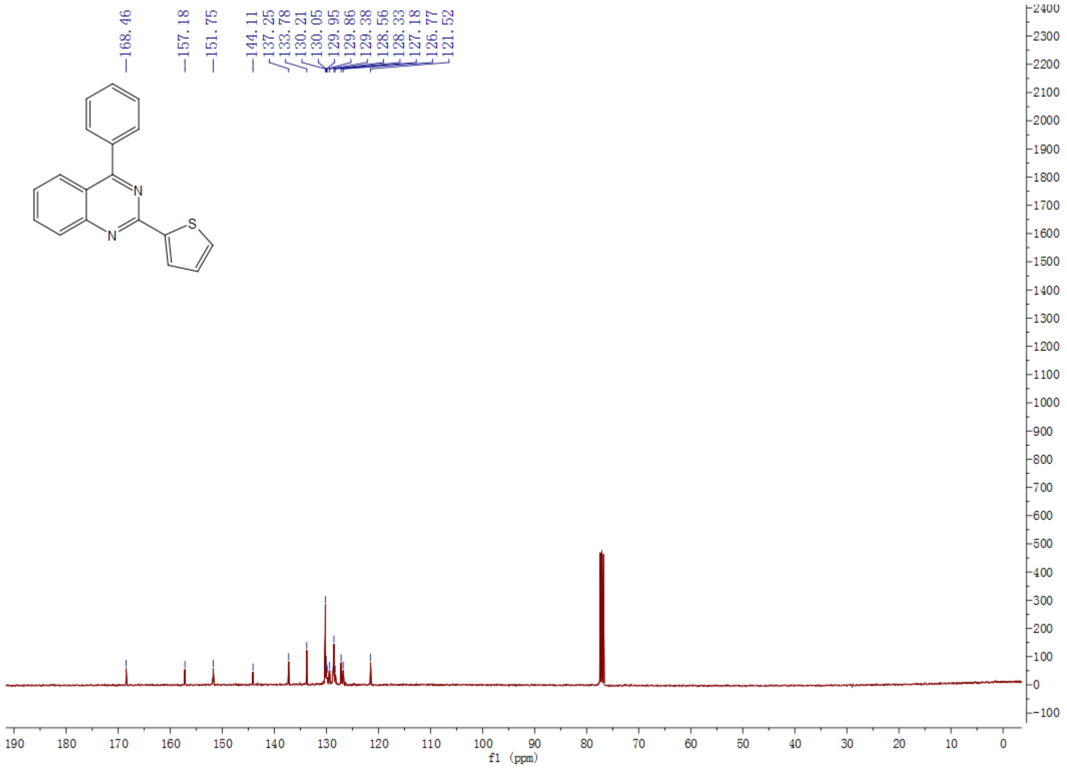

Figure S23. ^{1}H NMR of **3x** (500 MHz, $CDCl_3$) and ^{13}C NMR of **3x** (125 MHz, $CDCl_3$).

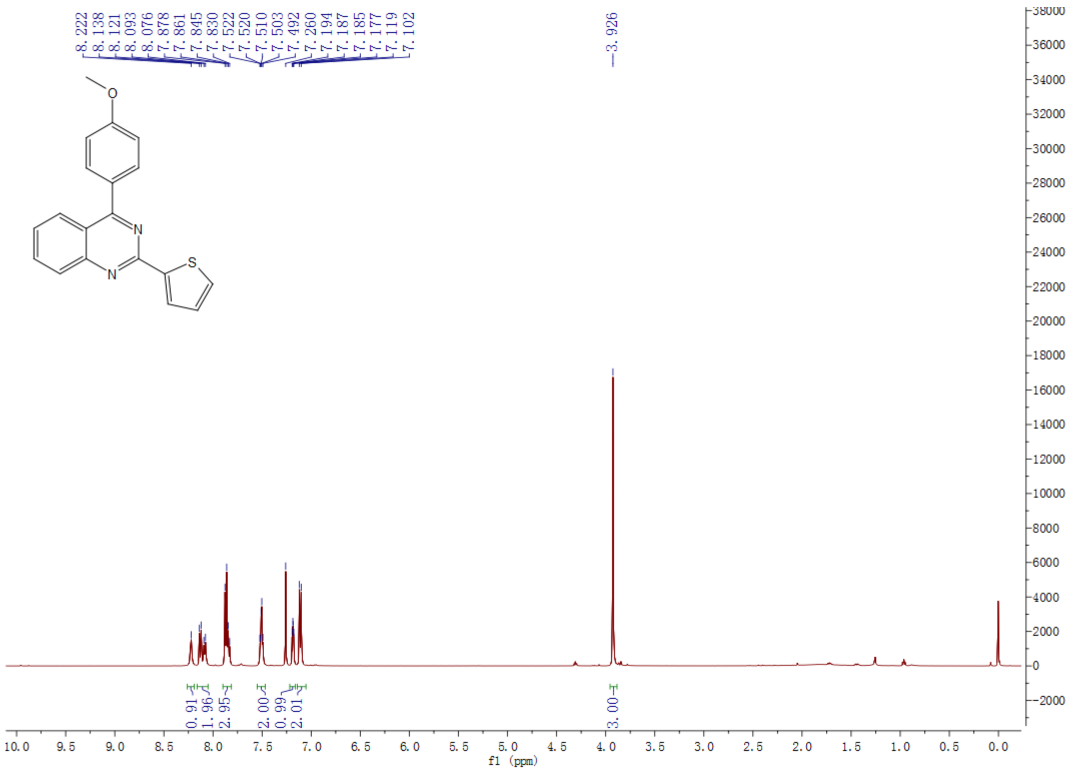

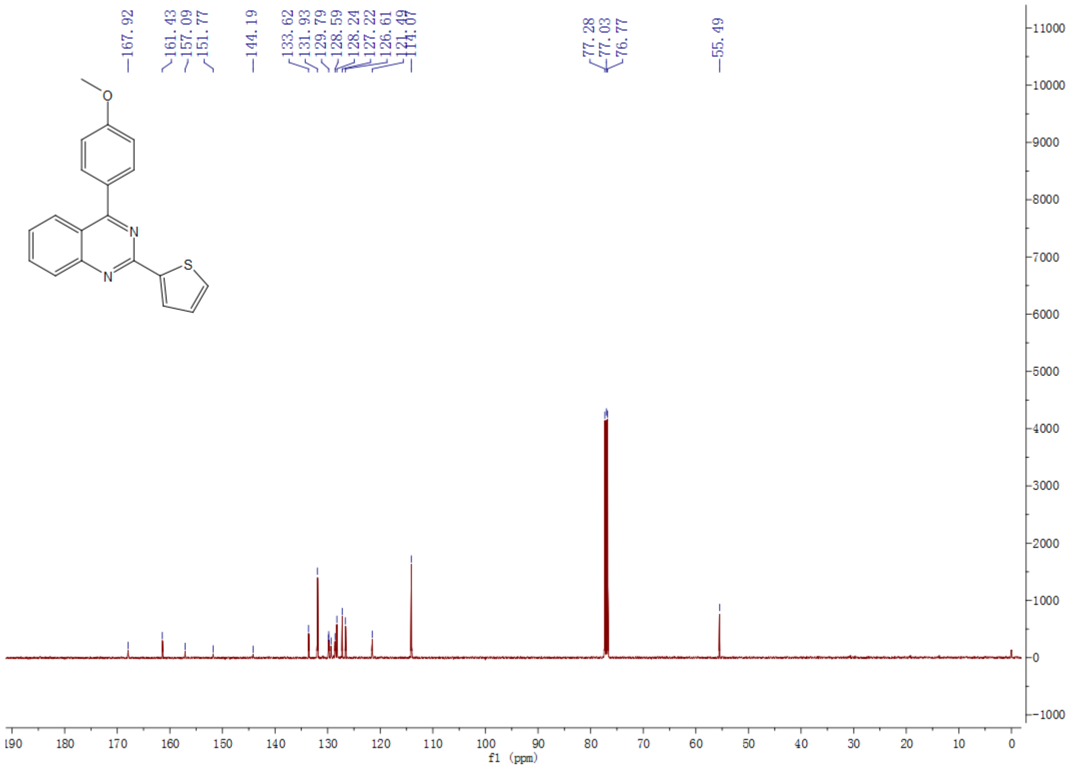

Figure S24. ^{1}H NMR of **3y** (500 MHz, $CDCl_3$) and ^{13}C NMR of **3y** (125 MHz, $CDCl_3$).

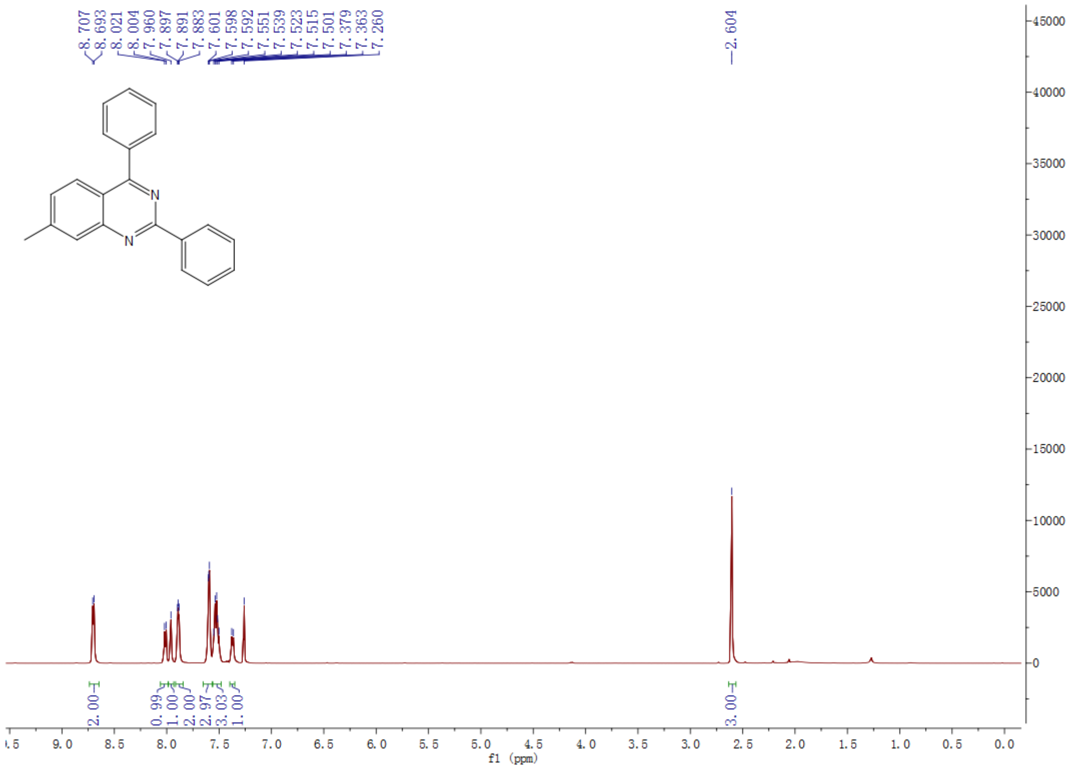

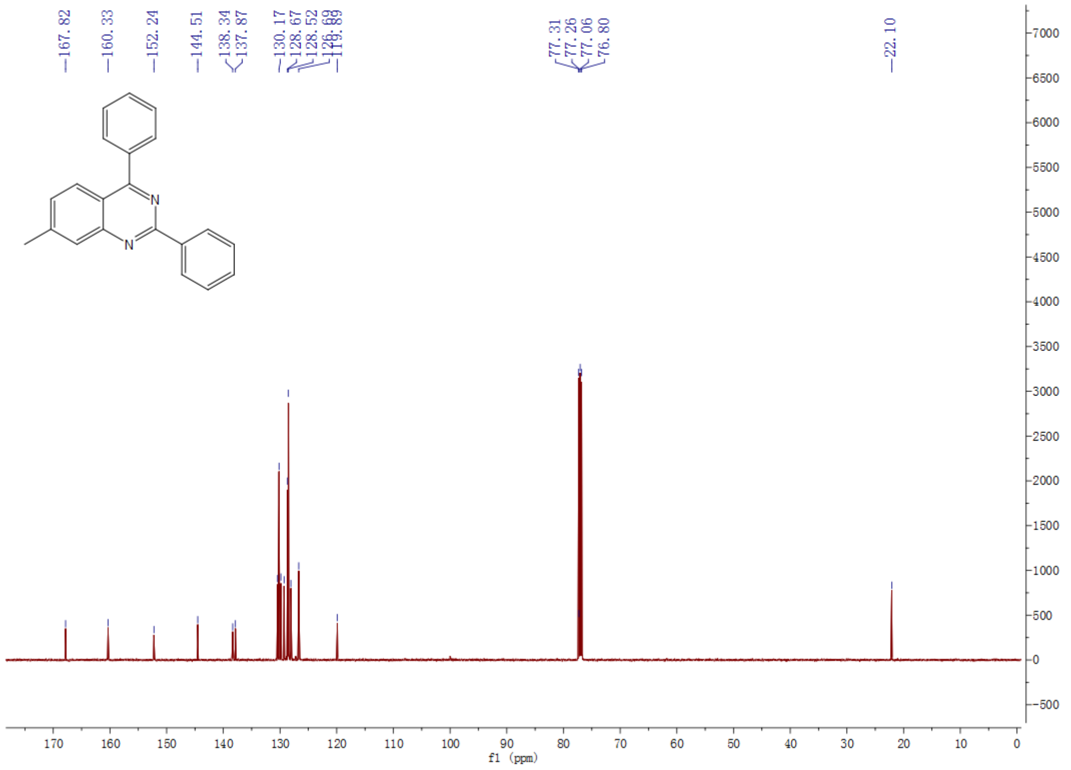

Figure S25. ^{1}H NMR of **3z** (500 MHz, $CDCl_3$) and ^{13}C NMR of **3z** (125 MHz, $CDCl_3$).

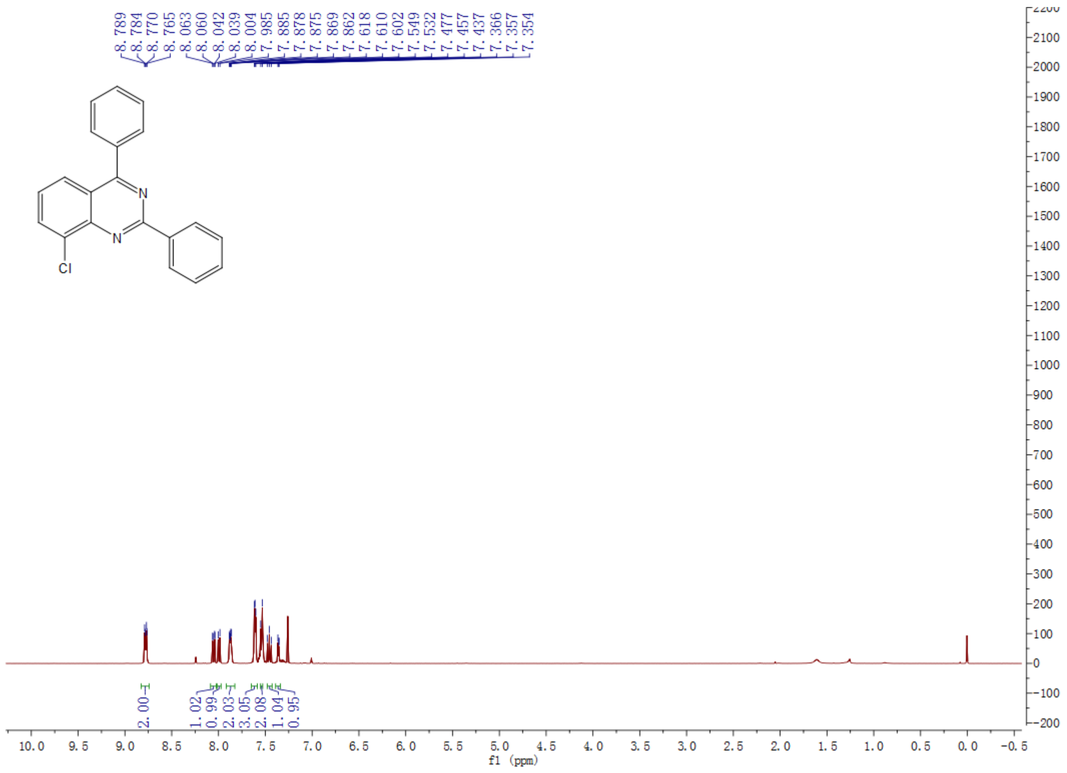

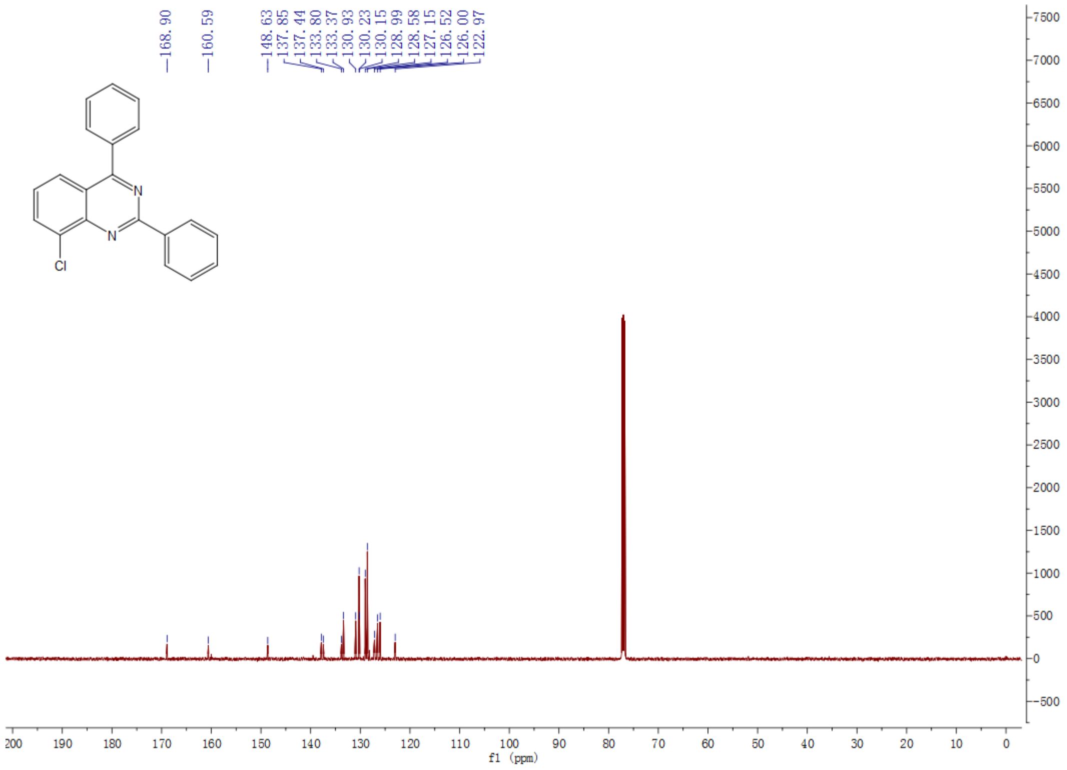

Figure S26. ^{1}H NMR of **4a** (500 MHz, $CDCl_3$) and ^{13}C NMR of **4a** (125 MHz, $CDCl_3$).

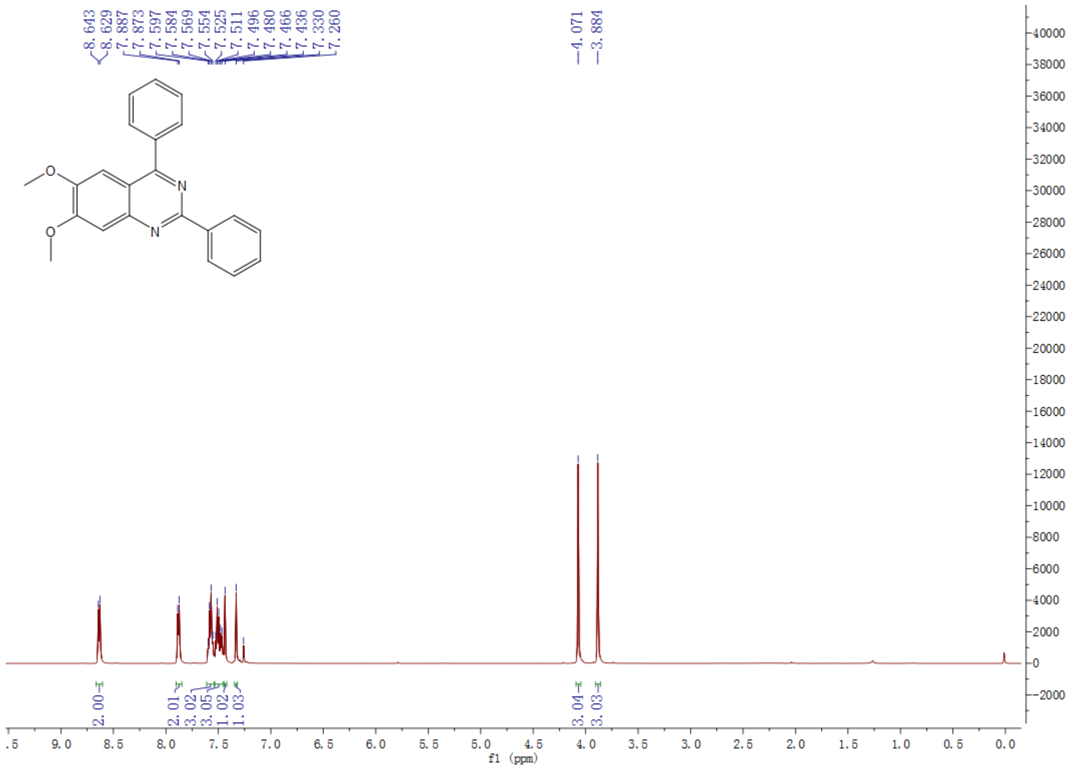

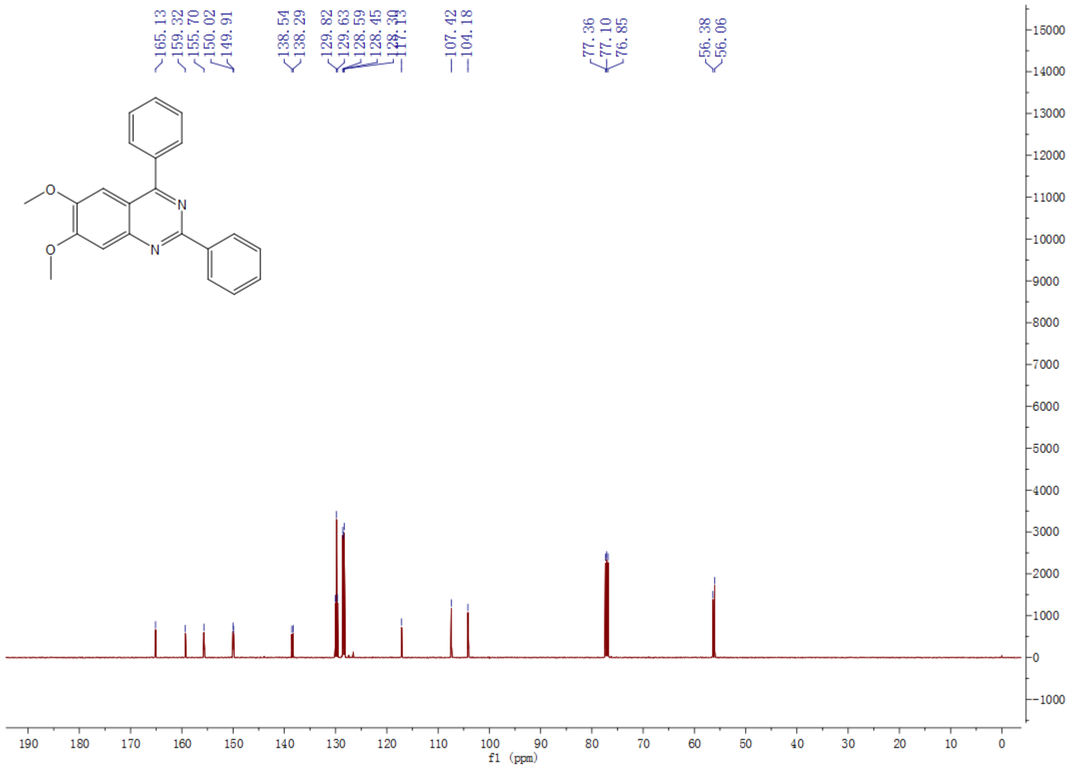

Figure S27. ^{1}H NMR of **4b** (500 MHz, $CDCl_3$) and ^{13}C NMR of **4b** (125 MHz, $CDCl_3$).

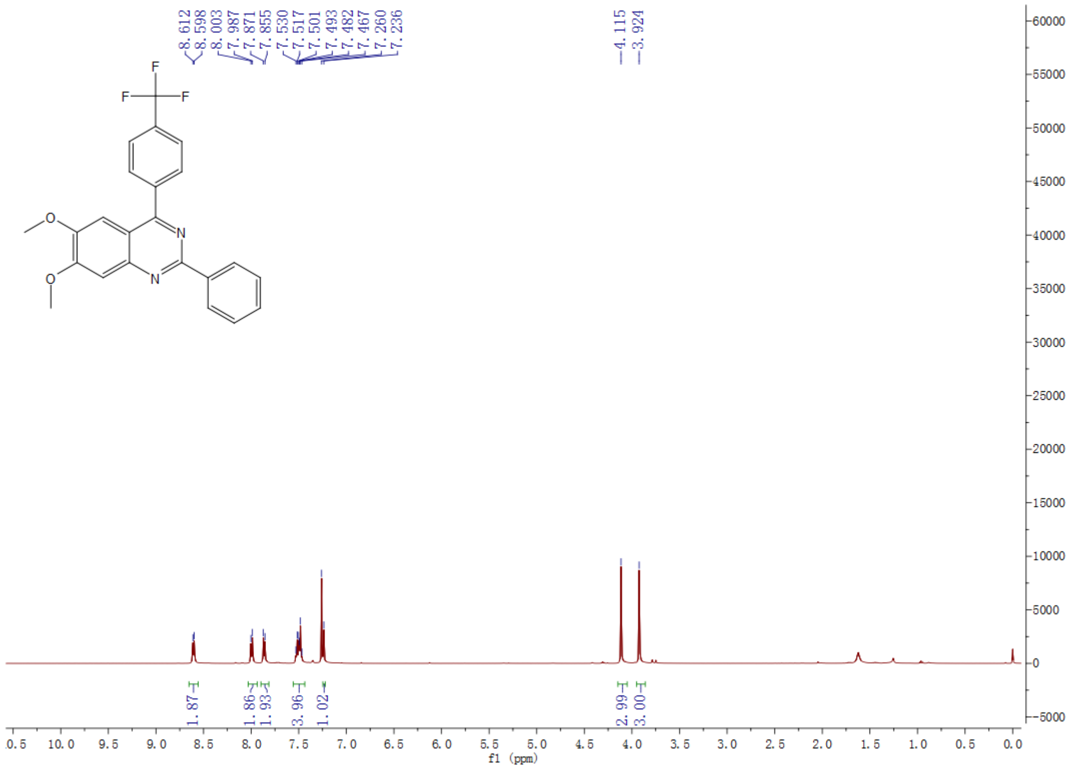

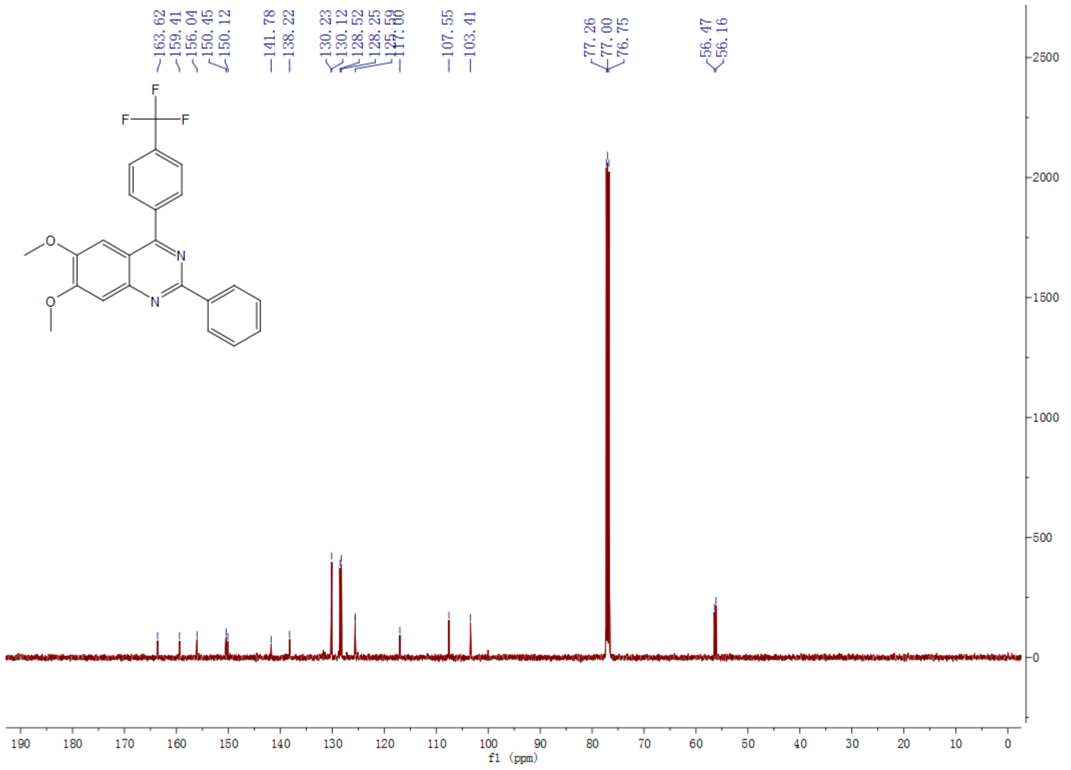

Figure S28. ^{1}H NMR of **4c** (500 MHz, $CDCl_3$) and ^{13}C NMR of **4c** (125 MHz, $CDCl_3$).

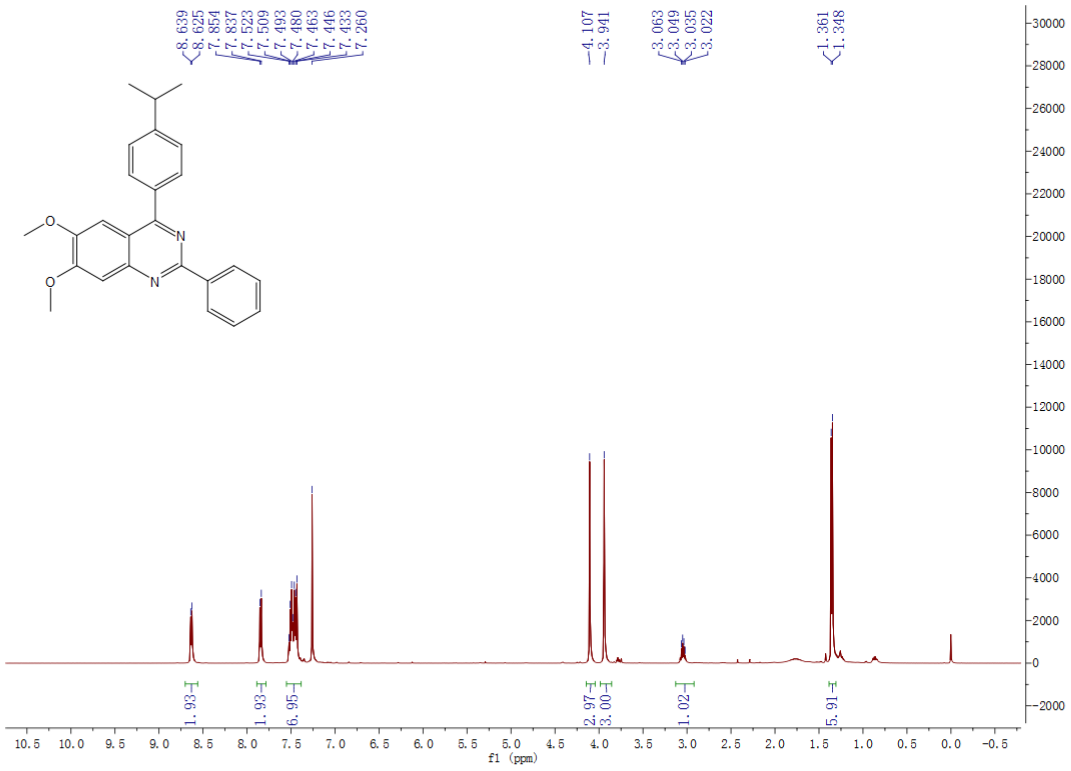

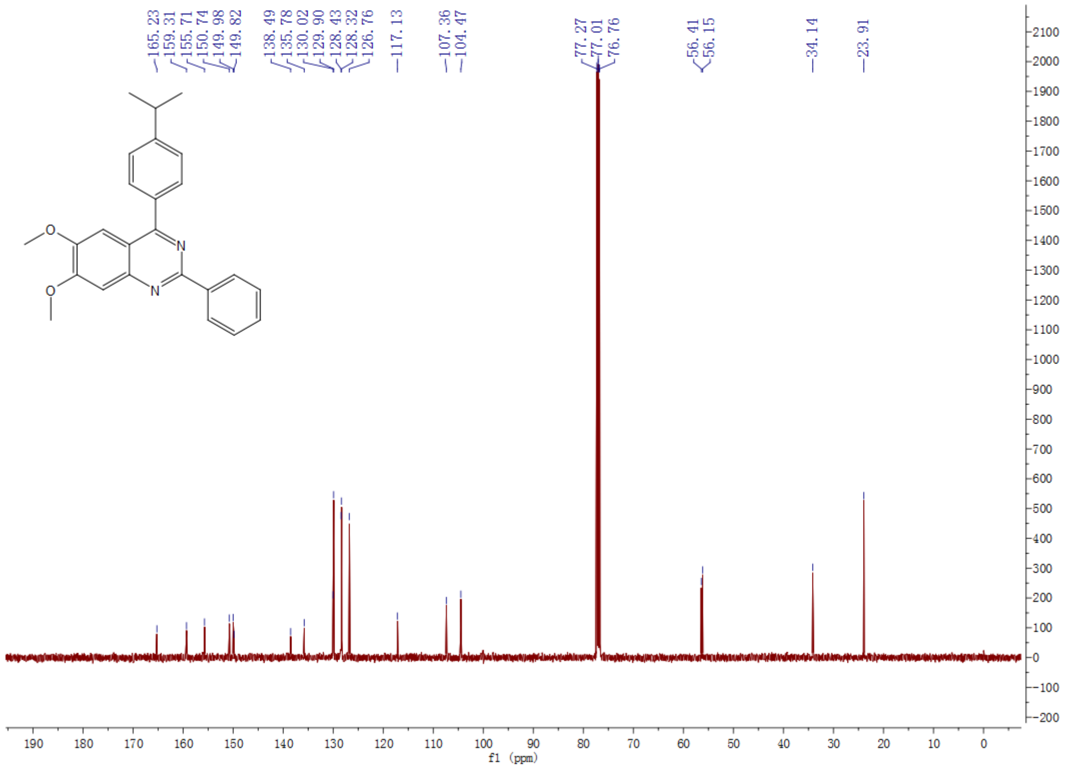

Figure S29. ^{1}H NMR of **4d** (500 MHz, $CDCl_3$) and ^{13}C NMR of **4d** (125 MHz, $CDCl_3$).

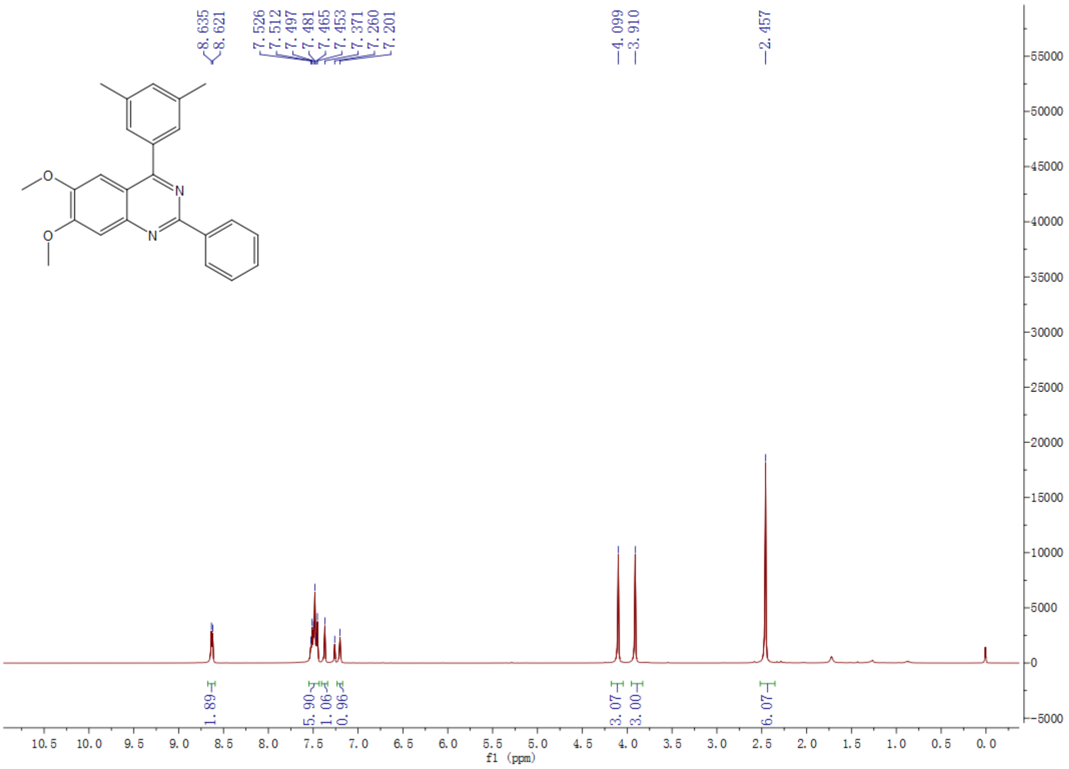

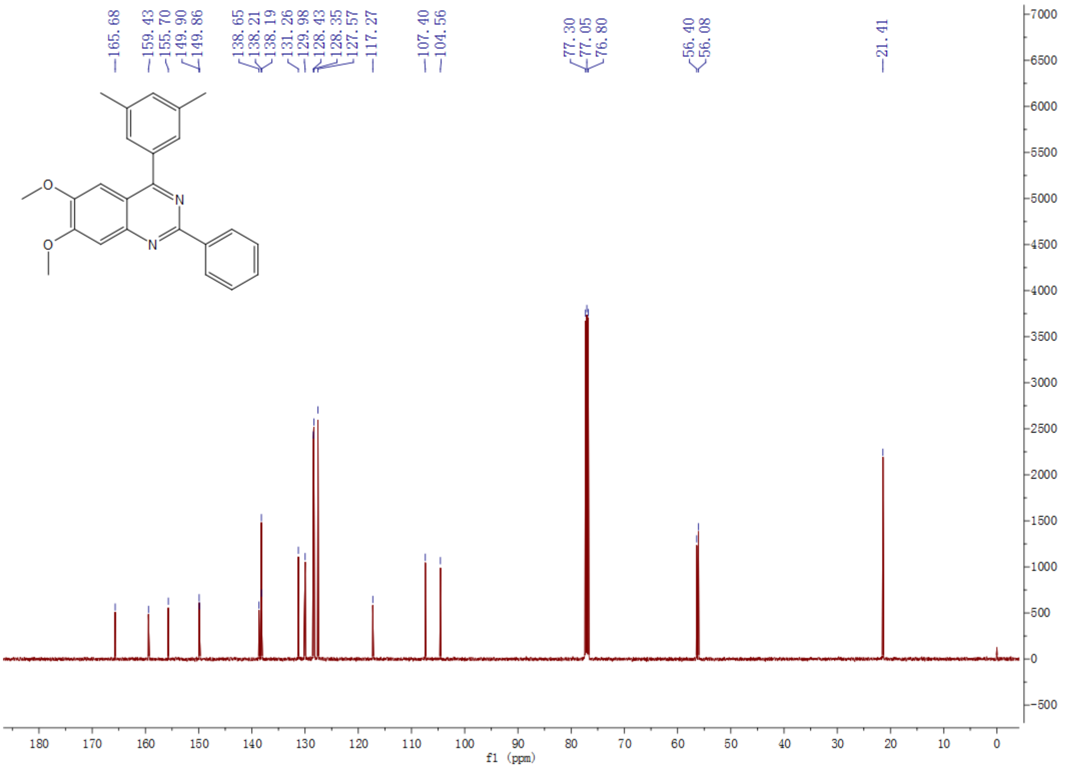

Figure S30. ^{1}H NMR of **4e** (500 MHz, $CDCl_3$) and ^{13}C NMR of **4e** (125 MHz, $CDCl_3$).

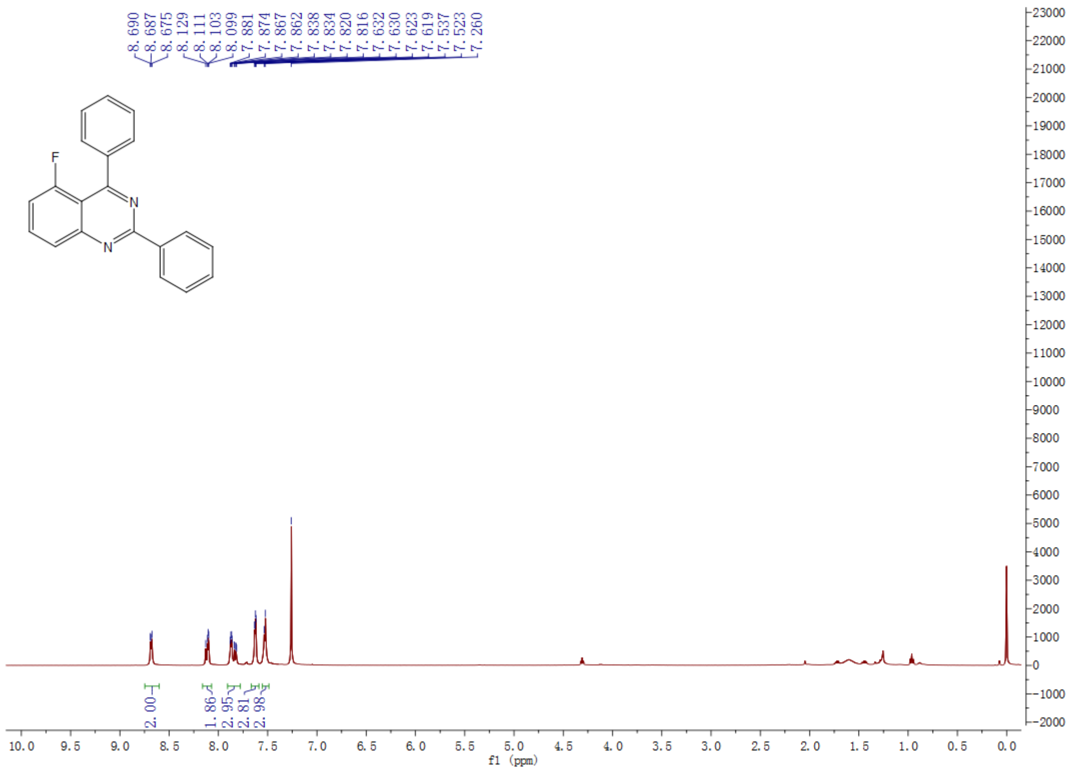

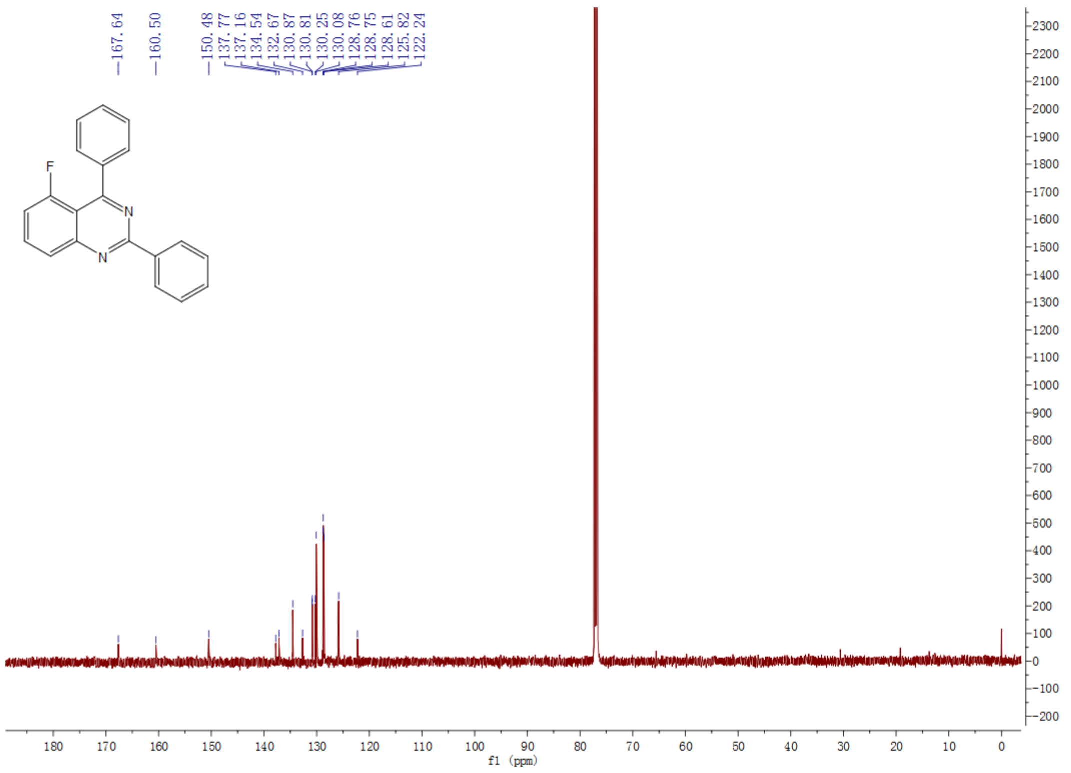

Figure S31. ^{1}H NMR of **4f** (500 MHz, $CDCl_3$) and ^{13}C NMR of **4f** (125 MHz, $CDCl_3$).

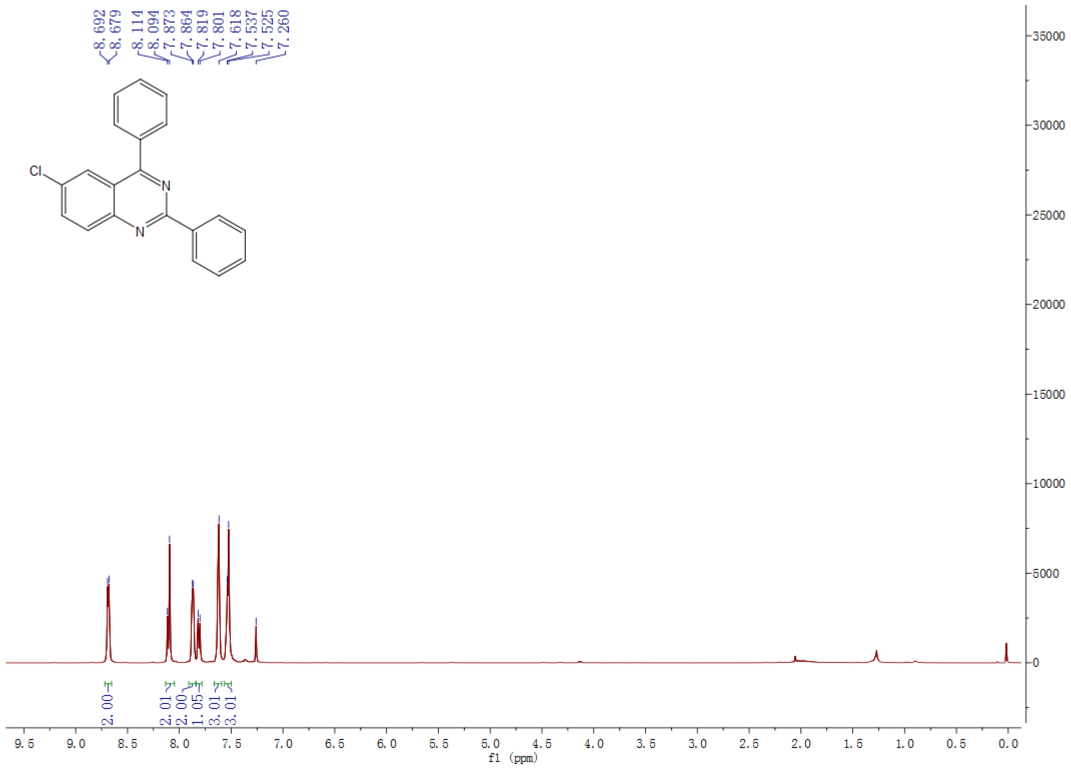

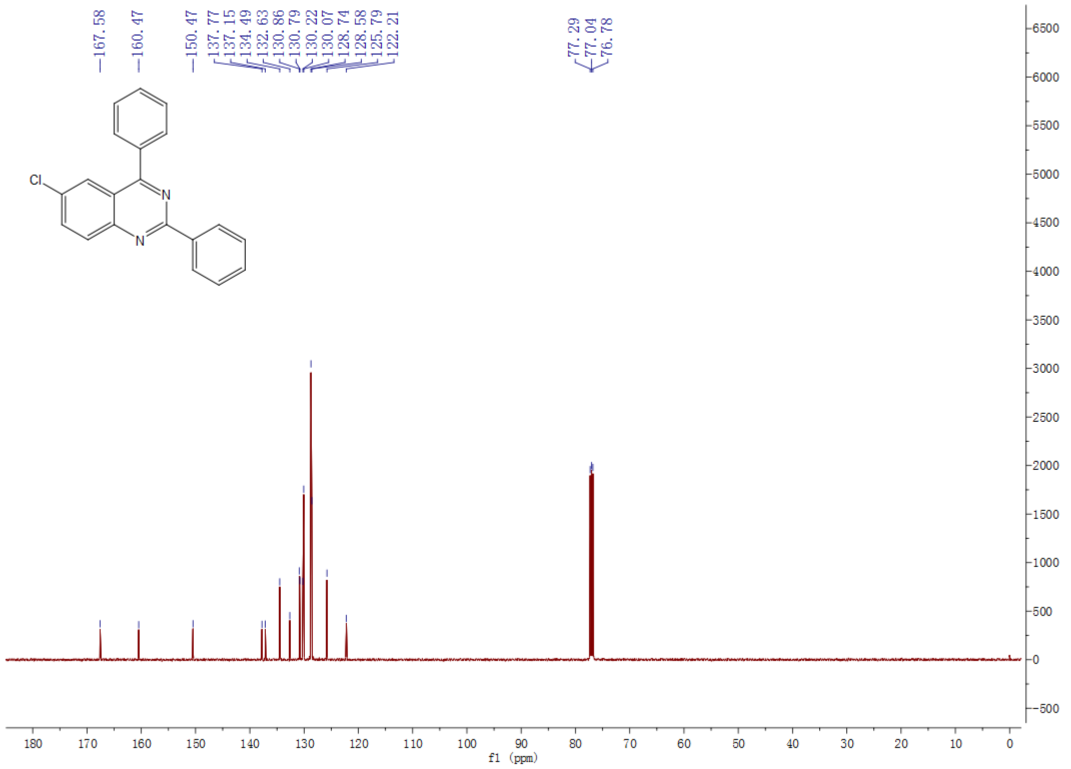

Figure S32. ^{1}H NMR of **4g** (500 MHz, $CDCl_3$) and ^{13}C NMR of **4g** (125 MHz, $CDCl_3$).

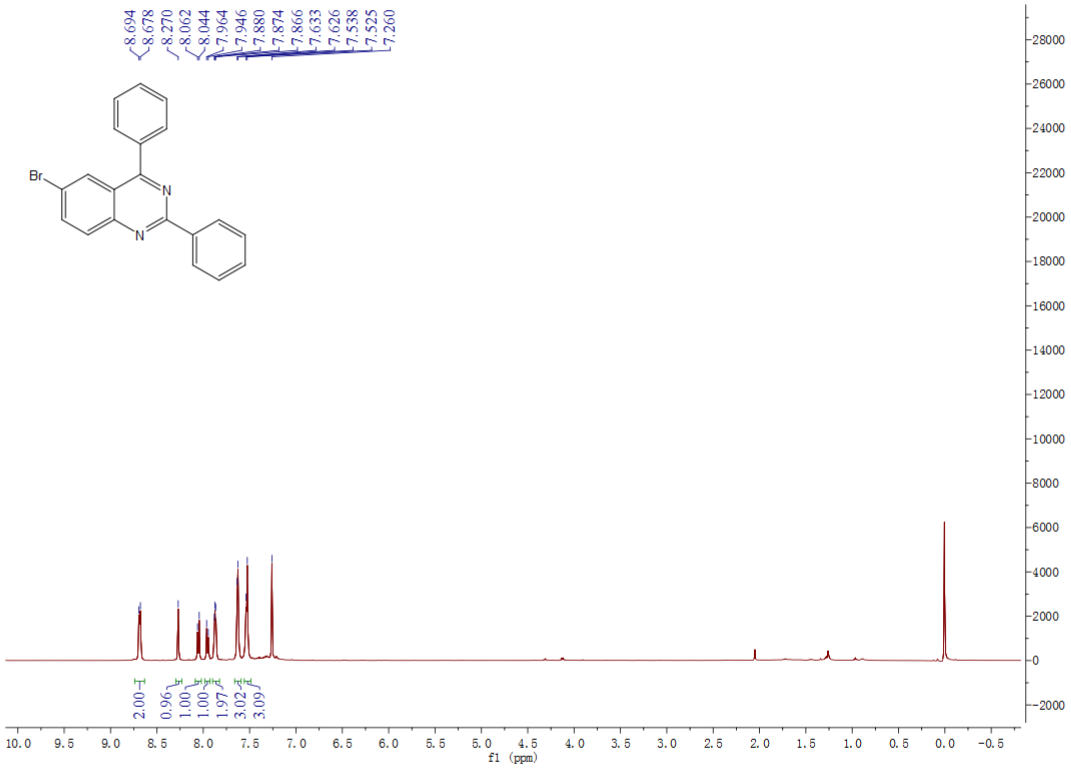

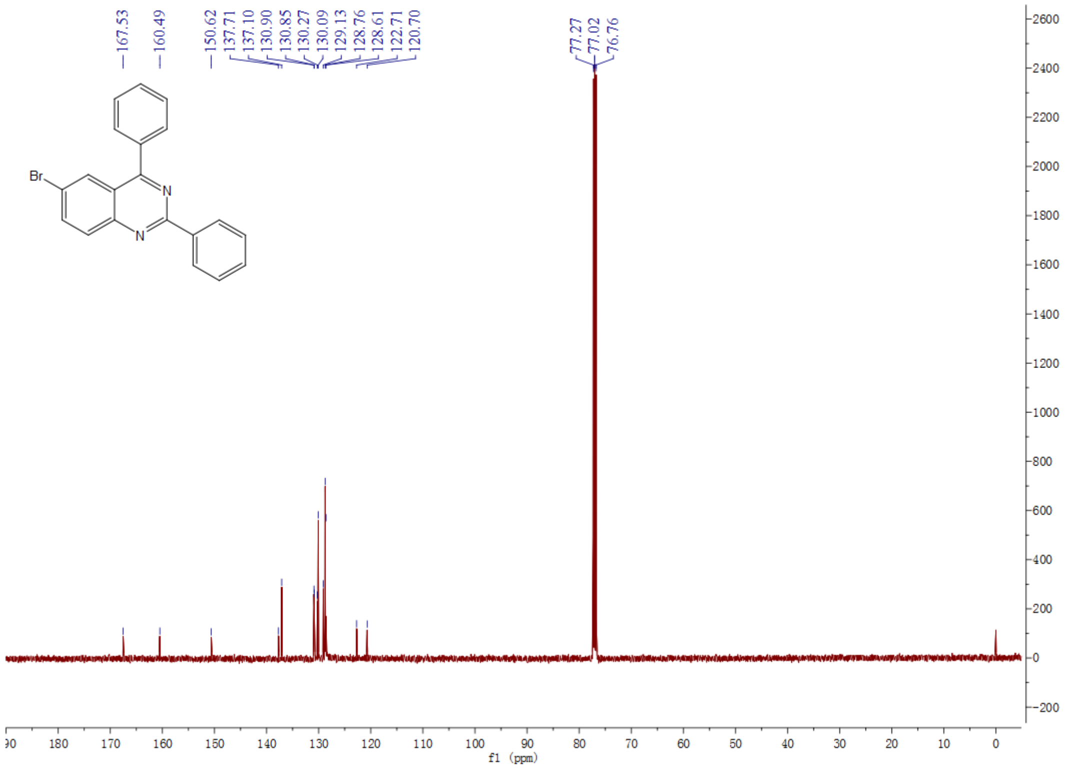

Figure S33. ^{1}H NMR of **4h** (500 MHz, $CDCl_3$) and ^{13}C NMR of **4h** (125 MHz, $CDCl_3$).

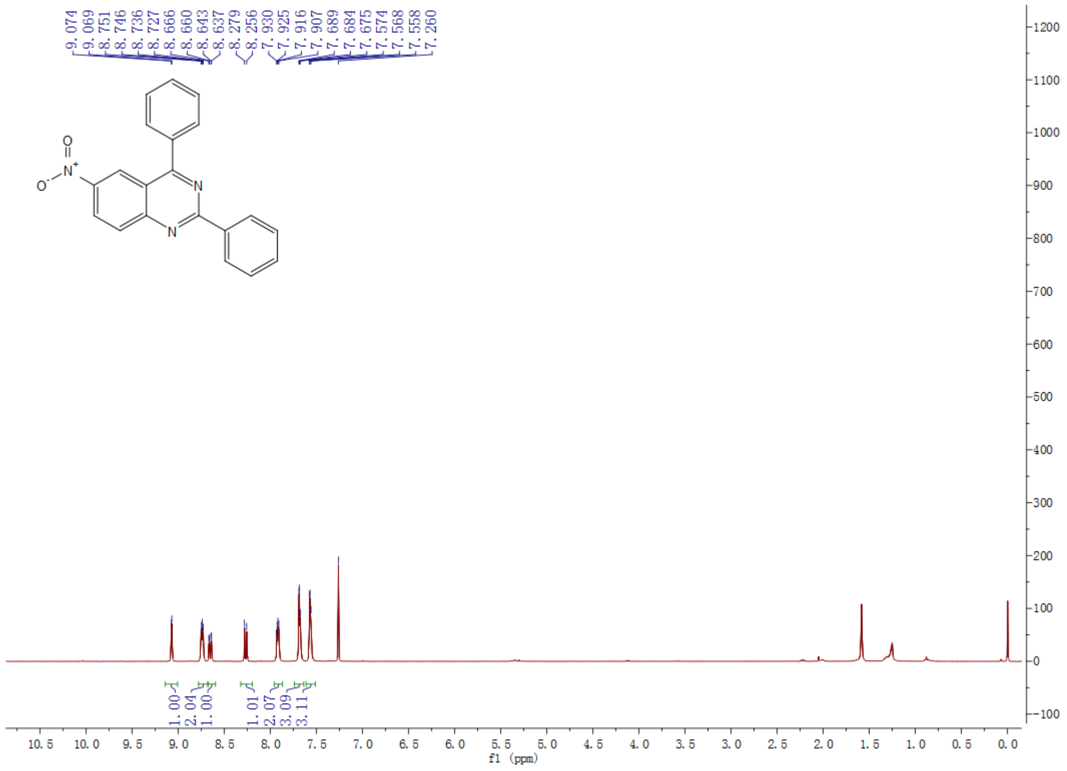

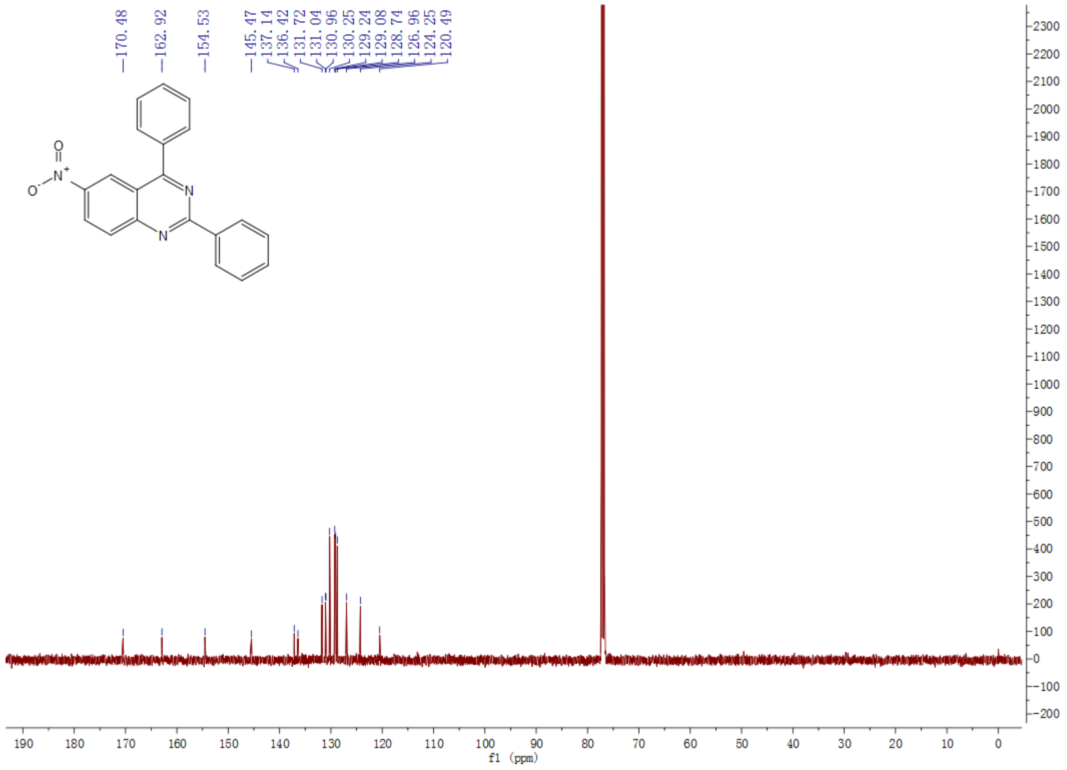

Figure S34. ^{1}H NMR of **4i** (500 MHz, $CDCl_3$) and ^{13}C NMR of **4i** (125 MHz, $CDCl_3$).

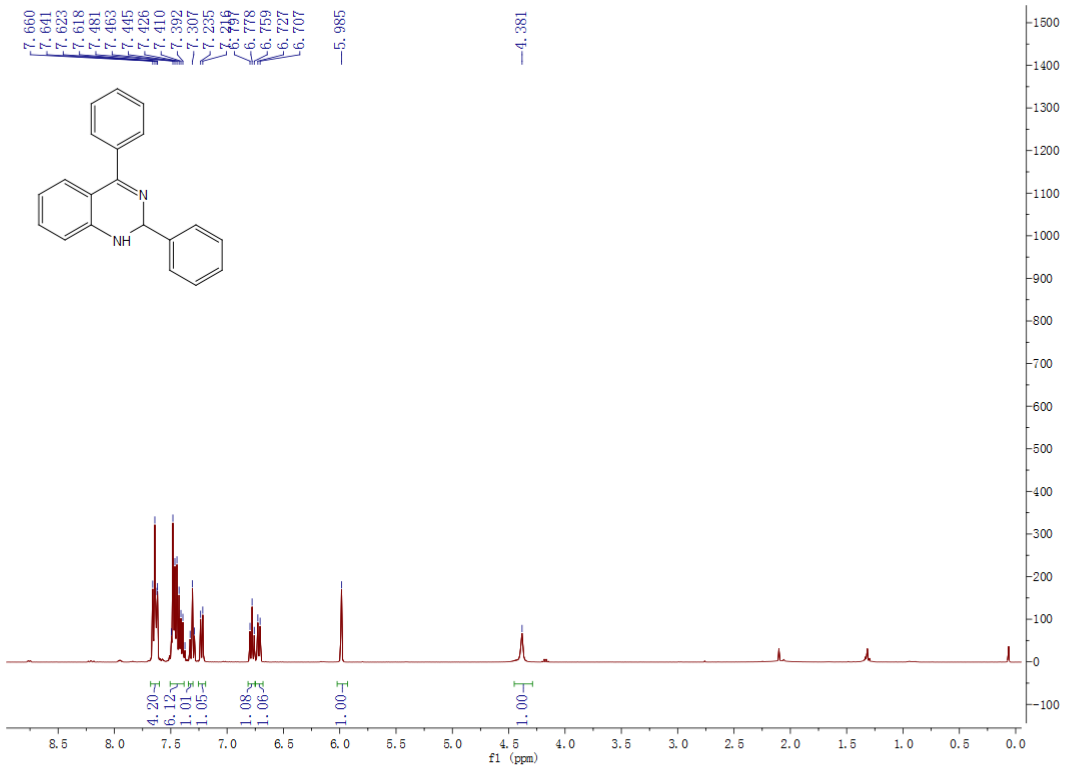

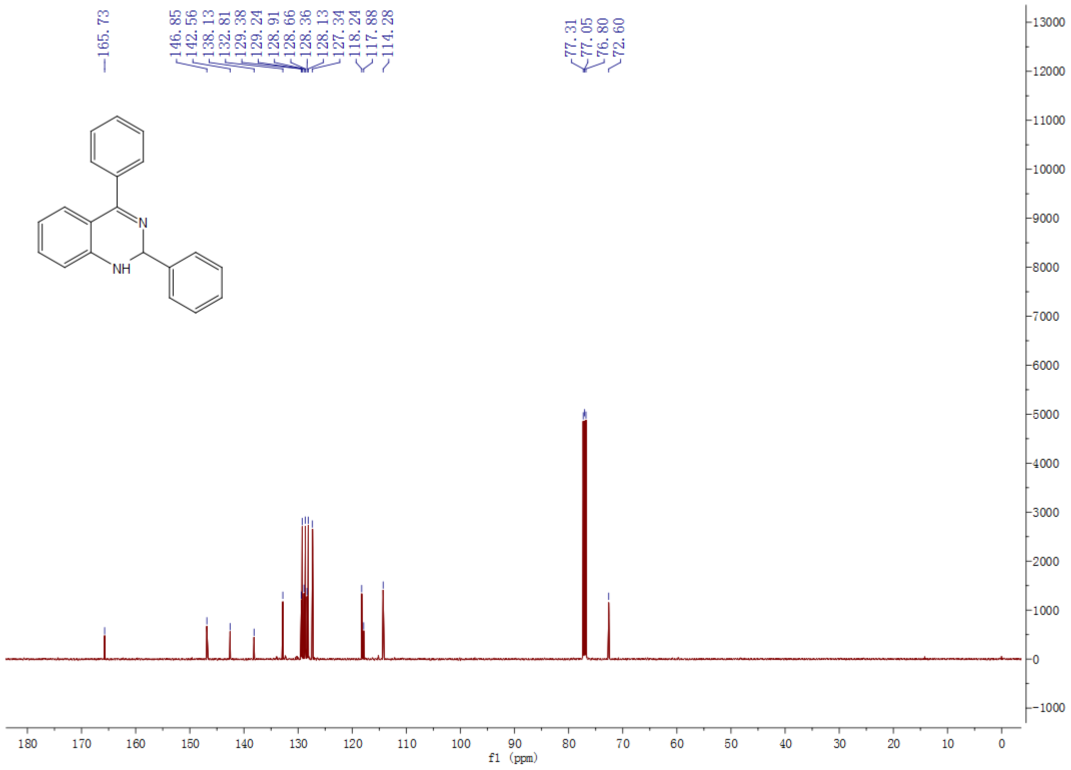

Figure S35. ^{1}H NMR of **5a** (500 MHz, $CDCl_3$) and ^{13}C NMR of **5a** (125 MHz, $CDCl_3$).
